# Dynamic changes in chromatin accessibility and gene expression involved in fetal myogenesis of Min pigs

**DOI:** 10.5713/ab.25.0034

**Published:** 2025-05-12

**Authors:** Xinhua Hou, Naiqi Niu, Xin Liu, Ligang Wang, Lixian Wang, Longchao Zhang

**Affiliations:** 1 Institute of Animal Science, Chinese Academy of Agricultural Sciences, Beijing, China

**Keywords:** Chromatin Accessibility, Fetal Period, Myogenesis, Pig, Transcriptome

## Abstract

**Objective:**

Chinese fat-type pig breeds possess good meat quality, but their growth rate and lean meat percentage are not dominant. Investigating the dynamic transcriptional regulation of skeletal muscle development could help improve meat yield in these breeds. However, little is known about chromatin accessibility and its association with gene expression during prenatal skeletal muscle development in these pigs.

**Methods:**

ATAC-seq and RNA-seq were performed to profile chromatin accessibility and transcriptome in skeletal muscle at 45, 70, and 100 days post-coitus (E45, E75, and E100) from two male and two female full-sib fetuses of Min pig.

**Results:**

This study demonstrated that the majority of ATAC-seq peak signals were located within 3 kb of transcription start sites. Notably, a greater number of genes associated with differential peaks were observed in the E100 vs. E45 comparison, relative to the E70 vs. E45 and E100 vs. E75 comparisons. This finding was consistent with the RNA-seq data, where the E100 vs. E45 comparison also exhibited the highest number of differentially expressed genes. Gene Ontology analysis of the RNA-seq data demonstrated that genes involved in skeletal muscle contraction, muscle fiber development, and energy metabolism were up-regulated during fetal development, while those associated with cell cycle regulation and proliferation were down-regulated. Integration of ATAC-seq and RNA-seq data identified a few differentially expressed genes associated with chromatin accessibility, with the overlapping genes primarily related to cell proliferation in the early fetal stage and metabolism in later stages.

**Conclusion:**

This study provides significant insights into the molecular mechanisms underlying fetal skeletal muscle development in Min pigs.

## INTRODUCTION

Porcine skeletal muscle is an important source of meat and protein for human consumption. The development and growth of skeletal muscle are crucial for meat yield. Skeletal muscle development is a multistep process involving the proliferation, differentiation, and fusion of myoblasts to form multi-nucleated myofibers [1]. In pigs, skeletal muscle fiber development occurs in two distinct growth waves: the formation of primary fibers between E35 and E55 days and the development of secondary fibers between E50 and E90 days [2]. It is commonly accepted that the total number of muscle fibers is established during the prenatal stage and remains fixed after birth. Postnatal muscle growth is characterized by hypertrophy and fiber-type conversion rather than an increase in fiber number [3]. Consequently, prenatal skeletal muscle development forms the foundation for porcine meat output and growth rate.

In eukaryotic cells, genomes are tightly compacted within the nucleus, which typically leads to transcriptional repression. In contrast, structurally loose chromatin is usually associated with active gene transcription [4]. Chromatin accessibility represents an important epigenetic mechanism regulating gene expression, as open chromatin regions facilitate the binding of transcription factors. Transposase-accessible chromatin assay with high-throughput sequencing (ATAC-seq) is widely employed to assess chromatin accessibility [5]. Several studies have investigated the dynamic changes in chromatin accessibility related to porcine skeletal muscle development. For instance, Yue et al [6] performed ATAC-seq analysis on Yorkshire pigs at E45, E70, and E100 days and found that chromatin accessibility was crucial for regulating the expression of essential genes involved in skeletal muscle development in a temporally and spatially specific manner. In addition, regulatory factors involved in postnatal skeletal muscle development in Landrace pigs were investigated using ATAC-seq [7]. Single-cell ATAC-seq analyses of Tibetan pigs and Duroc×Tibetan pigs further revealed dynamic changes in chromatin accessibility along the myogenic differentiation trajectory, identifying upstream regulatory networks relevant to early embryonic myogenesis [8]. ATAC-seq was also utilized to characterize the regulatory and expressed genomic regions during skeletal muscle development in both prenatal and postnatal Yorkshire×Landrace pigs [9].

In comparison to Western lean-type pig breeds, Chinese indigenous fat-type pig breeds are known for their superior meat quality, although they exhibit slower growth rates and lower lean meat percentages. A previous study has shown that skeletal muscle development in Western and Chinese indigenous pig breeds is influenced by distinct gene expression patterns [10]. However, limited information is available on chromatin accessibility and its association with gene expression in Chinese indigenous pig breeds during prenatal skeletal muscle development. In this study, the longissimus dorsi muscle samples from Min pigs—a representative Chinese indigenous breed—were collected at E45, E70, and E100 days, respectively. RNA-seq and ATAC-seq were subsequently performed to investigate the dynamic changes in gene expression and chromatin accessibility concerning fetal skeletal muscle development.

## MATERIALS AND METHODS

### Animal individuals and samples collection

The Min pigs used in this study were raised under the same conditions at an experimental pig farm of the Institute of Animal Science, CAAS in Beijing, China. Three sows were sacrificed at 45, 70, and 100 days post coitus. Longissimus dorsi muscle tissue was collected from two male and two female full-sib fetuses at each developmental stage. All tissue samples were immediately frozen in liquid nitrogen.

### Library for ATAC-seq

ATAC-seq libraries were constructed from all 12 samples. Approximately 50,000 nuclei were collected after homogenization of the muscle sample. The transposition reaction was performed to isolate transposed DNA fragments by incubating the samples with Nextera Tn5 transposase at 37°C for 30 minutes. Library construction was completed through eight cycles of PCR amplification, followed by purification using the MinElute PCR Purification kit (Qiagen). The quality of the library was assessed using an Agilent Bioanalyzer 2000 (Agilent). Paired-end sequencing was subsequently performed on a NovaSeq 6000 platform.

### Library for RNA-seq

Total RNA from skeletal muscle was extracted with a TruSeq Stranded Total RNA Ribo-Zero H/M/R Kit (Illumina). RNA quality was assessed with an Agilent 2100 bioanalyzer (Agilent). High-quality RNA was then reverse-transcribed into cDNA and ligated with sequencing adapters. Twelve libraries were generated through PCR amplification and subjected to paired-end sequencing on a HiSeq X platform.

### ATAC-seq analysis

Trimmomatic (0.30) was used to eliminate adapters and low-quality sequences to obtain clean reads. The trimmed reads were aligned to the porcine reference genome (Susscrofa 11.1) using BWA-MEM. Mapped reads were further filtered with SAMtools (1.14) to exclude duplicate alignments, fragments with low mapping quality (less than 30), and reads mapping to the Y chromosome and mitochondrial genomes. Accessible chromatin regions (ACRs) were identified using MACS2 (2.2.7) for peak detection, with a qvalue threshold of less than 0.05. Peak annotation was performed with the HOMER annotatePeaks function. ACRs within 3 kb of each gene’s transcription start site (TSS) were visualized using deepTools. Differential peak analysis was conducted with the R DiffBind package, applying criteria of fold-change>2 and p-value<0.05. Sequencing signals from bigWig files, generated by bamCoverage in deepTools, were visualized using Integrated Genomics Viewer (IGV) 2.16.2. Transcription factor binding sites were predicted by AnimalTFDB v4.0 (http://guolab.wchscu.cn/AnimalTFDB4/#/).

### RNA-seq analysis

The clean data were derived from the raw data by trimming sequencing adapters and low-quality reads with Trimmomatic (0.30). The filtered reads were then mapped to the porcine reference genome (Susscrofa 11.1) using HISAT2 (2.2.1). Sorted BAM files were generated with SAMtools (1.14). Gene read counts were quantified using FeatureCounts (2.0.5), and differential expression analysis was conducted with DESeq2 (1.30.1) in R. Genes with a fold-change>2 and an adjusted p-value<0.05 were considered differentially expressed. Time series expression analysis was performed using the Mfuzzy R package.

### Functional analysis

Gene Ontology (GO) and Kyoto Encyclopedia of Genes and Genomes (KEGG) pathway analyses were performed using the DAVID database (https://david.ncifcrf.gov/tools.jsp). A p-value threshold of 0.05 was applied to determine the significantly enriched terms.

## RESULTS

### Differences in chromatin accessibility during porcine skeletal muscle development

**Figure 1:**
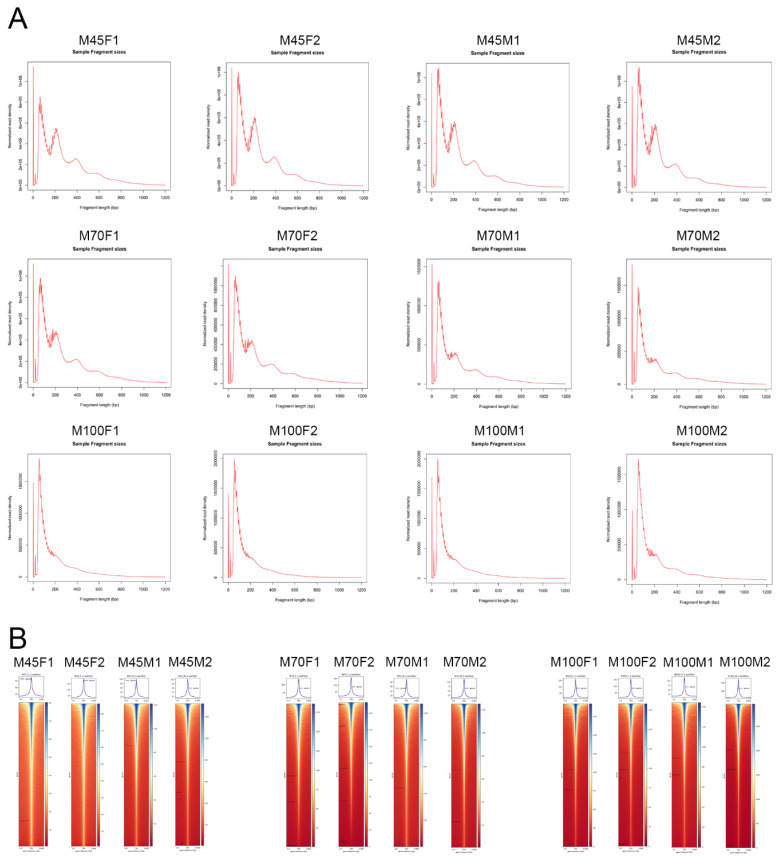
Basic features of the ATAC-seq data. (A) Distribution of fragment lengths. (B) Enrichment of ATAC-seq signals around the transcription start site.

**Figure 2:**
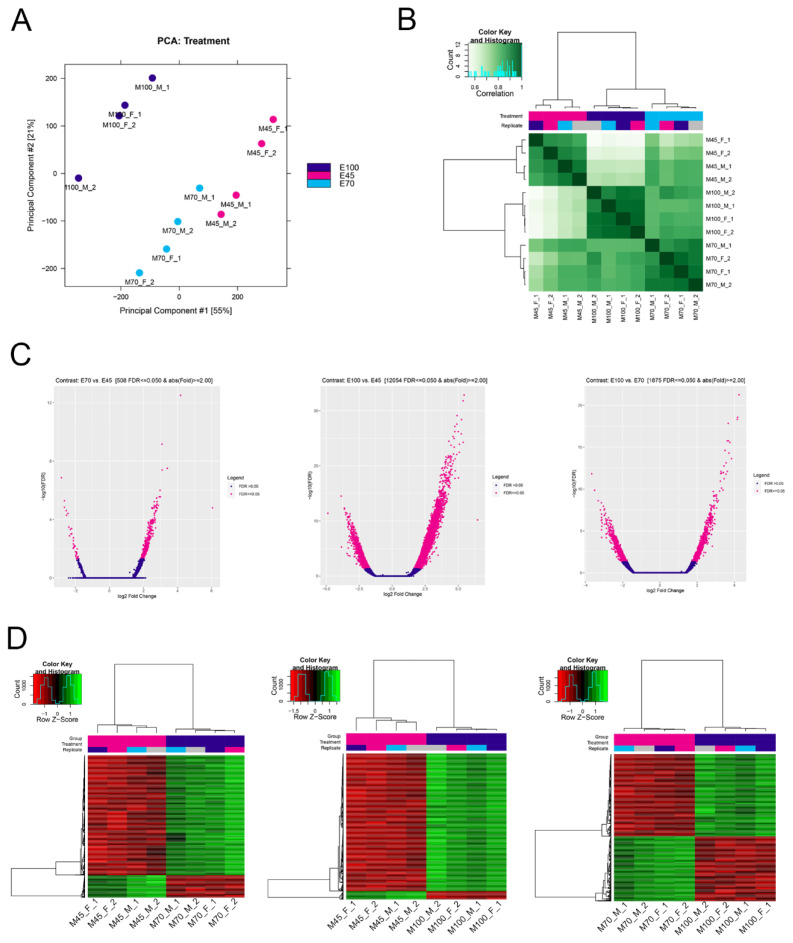
Differential analysis of chromatin accessibility. (A) Principal component analysis of all samples on peak signal. (B) Heatmap showing clustering of samples. (C) Volcano plots depicting differential peaks for E70 vs. E45, E100 vs. E45, and E100 vs. E70. (D) Heatmaps illustrating differential peaks for E70 vs. E45, E100 vs. E45, and E100 vs. E70.

A total of 183–193 million clean reads were generated for ATAC-seq, with approximately 149–173 million clean reads uniquely mapped to the reference genome (Supplement 1). The quality of the ATAC-seq libraries was assessed based on the distribution of insert fragment sizes and peak signals. As shown in Figure 1A, the nucleosome-free fragment (~100 bp) was the predominant peak in all samples, followed by mononucleosome fragment (~200 bp), dinucleosome fragment (~400 bp), and trinucleosome fragment (~600 bp), consistent with the expected distribution for open chromatin regions [6]. Given that open chromatin is associated with gene transcription, the majority of peak signals were located within 3 kb of the TSS (Figure 1B). To further evaluate the quality of the ATAC-seq data, principal component analysis (PCA) and a correlation heatmap based on the peak profiles were conducted. These analyses indicated that samples from the same developmental stage were similar, while samples from different stages exhibited distinct differences (Figures 2A, 2B). Differential peak analysis revealed the open chromatin sites associated with myogenesis during the fetal period. Specifically, the number of differential peaks was 393 for E70 vs. E45 (363 up-regulated and 30 down-regulated at E70), 1,172 for E100 vs. E70 (527 up-regulated and 645 down-regulated at E100), and 8,265 for E100 vs. E45 (6,339 up-regulated and 1,926 down-regulated at E100) (Figure 2C). Heatmaps demonstrated that these differential peaks effectively distinguish between the different fetal stages (Figure 2D; Supplement 2).

### Identification of functional genes related to porcine skeletal muscle development

**Figure 3:**
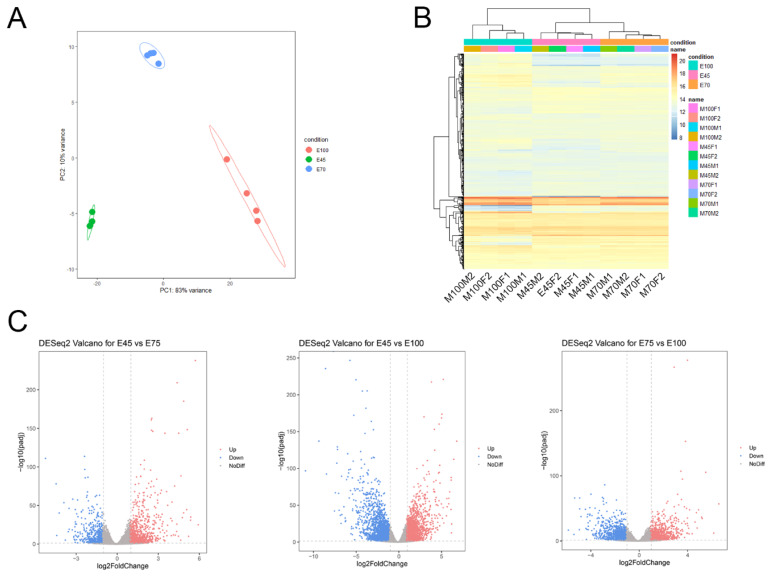
Differential analysis of the transcriptome. (A) Principal component analysis of samples based on read counts. (B) Clustering of samples according to read counts. (C) Volcano plots illustrating differential gene expression.

**Figure 4:**
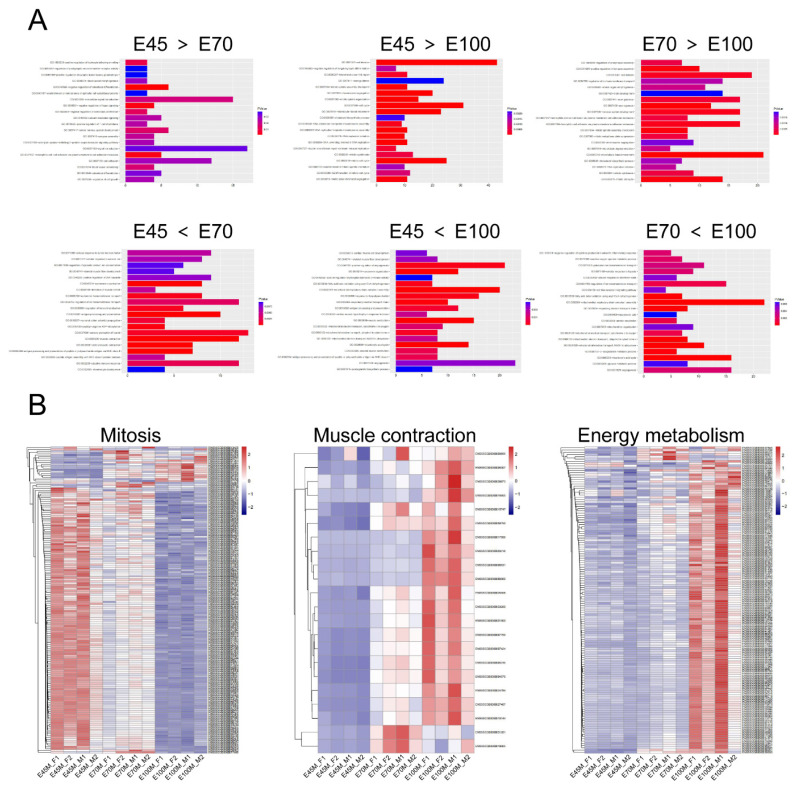
Functional enrichment analysis of differential genes from RNA-seq. (A) GO enrichment analysis of differentially expressed genes. (B) Heatmap depicting differentially expressed genes associated with mitosis, muscle contraction, and energy metabolism. GO, Gene Ontology.

RNA-seq generated between 22 and 35 million clean reads, with 19 to 30 million reads uniquely mapped to the reference genome (Supplement 3). PCA and hierarchical clustering analysis revealed that samples from different fetal periods clustered into three distinct groups, each with unique gene expression profiles, indicating high data quality for subsequent analysis (Figures 3A, 3B). Pairwise comparisons of the three fetal periods revealed the following: 963 up-regulated and 496 down-regulated genes at E70 compared to E45; 1,184 up-regulated and 1,047 down-regulated genes at E100 compared to E70; and 2,203 up-regulated and 1,802 down-regulated genes at E100 compared to E45 (Figure 3C). To elucidate the functions of differentially expressed genes across the fetal periods, GO and KEGG enrichment analyses were conducted with a significance threshold of p-value<0.05 (Supplement 4). The biological process category in the E70 vs. E45 comparison revealed that up-regulated genes at E45 were enriched in cell adhesion, while up-regulated genes at E70 were enriched in skeletal muscle fiber development and contraction. Genes with reduced expression at E100 were primarily associated with mitotic cell division and cell cycle processes (Figure 4A). Conversely, genes with elevated expression at E100, compared to E45 and E70, were significantly enriched in pathways related to skeletal muscle contraction, mitochondrial organization, mitochondrial electron transport, mitochondrial respiratory chain complex I assembly, and the tricarboxylic acid cycle (Figure 4A). These findings indicated that the formation of muscle fibers was nearly complete by E100, characterized by enhanced energy metabolism and decreased cell proliferation. Consequently, with advancing fetal age, the expression of genes related to mitosis decreased, whereas those associated with muscle contraction and energy metabolism increased (Figure 4B; Supplement 5).

**Figure 5:**
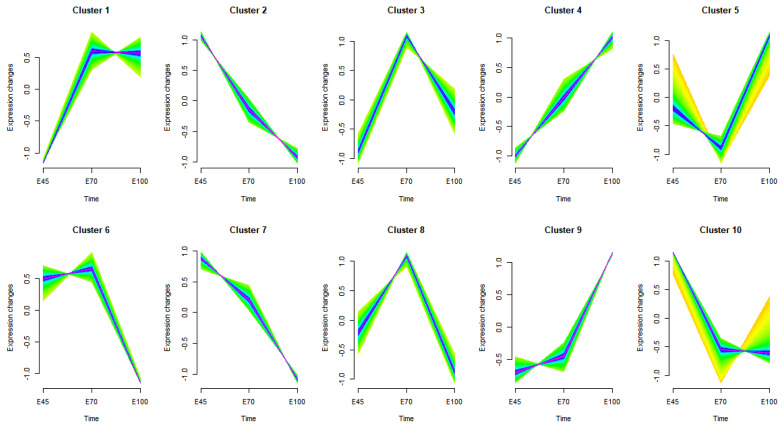
Trend analysis of RNA–seq data. Clustering of genes based on their expression patterns at E45, E70, and E100.

To further validate the developmental and physiological characteristics of muscles at various prenatal stages, time series expression analysis was employed to categorize genes based on their expression patterns (Figure 5). Additionally, the heatmaps corresponding to each cluster have been included for comprehensive reference (Supplement 6). Genes in cluster 4 exhibited a progressive increase in expression and were significantly enriched in biological processes related to skeletal muscle contraction and muscle tissue development. Conversely, genes in clusters 2 and 7 demonstrated a progressive decrease in expression and were predominantly associated with cell division and the mitotic cell cycle (Supplement 7). These findings align with the GO enrichment results for differently expressed genes. Genes in cluster 10, which showed peak expression at E45, were involved in cell growth, the Wnt signaling pathway, and embryonic development. Cluster 3 and 8 exhibited the highest expression levels at E70, with genes in these clusters participating in the regulation of transcription by RNA polymerase II, reflecting the activation of many genes to support muscle differentiation. At E100, gene expression in cluster 9 was significantly elevated, with these genes implicated in the regulation of mitochondrial respiratory chain complex I assembly, tricarboxylic acid cycle, fatty acid beta-oxidation, and mitochondrial electron transport (Supplement 7). This increase in expression at E100 is consistent with the up-regulation of genes involved in energy metabolism.

### Integration of chromatin accessibility and gene expression in skeletal muscle

**Figure 6:**
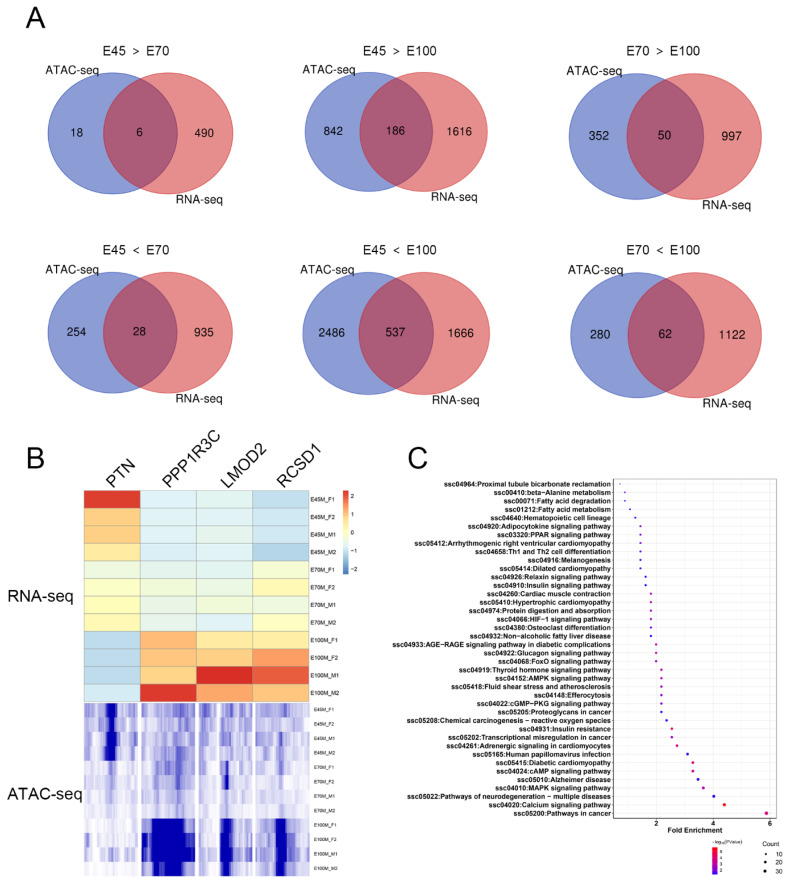
Integrative analysis of ATAC-seq and RNA-seq. (A) Intersection of differentially expressed genes and genes with differential peaks between E45 vs. E70, E45 vs. E100, and E70 vs. E100. (B) Expression heatmap and ATAC-seq peak signals for PTN, PPP1R3C, LMOD2, and RCSD1. (C) KEGG pathway enrichment analysis of overlapping genes in E45<E100 comparison. KEGG, Kyoto Encyclopedia of Genes and Genomes.

**Figure 7:**
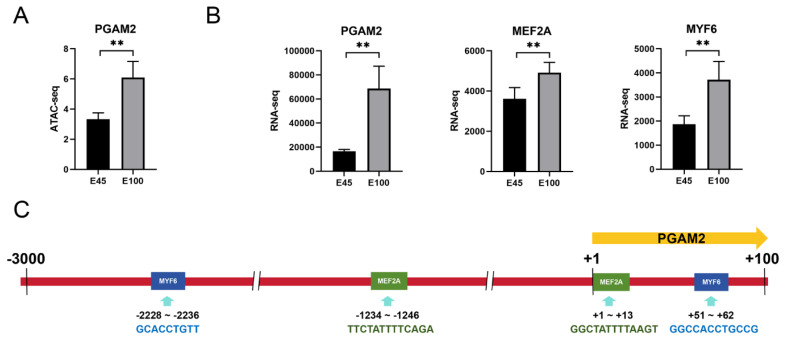
Regulatory mechanism of chromatin accessibility on gene expression. (A) Differential ATAC-seq peak signals of PGAM2 between E45 and E100. (B) Expression levels of PGAM2, MEF2A, and MYF6 at E45 and E100. (C) Predicted binding sites of MEF2A and MYF6 in the promoter region of PGAM2. ** Green boxes: MEF2A binding sites. Blue boxes: MYF6 binding sites. “+1”: PGAM2 transcription start site. Yellow arrow: transcriptional direction of PGAM2.

Chromatin accessibility reflects the capacity of chromatin DNA to interact with other molecules, such as transcription factors, and is directly correlated with gene expression levels [11]. To identify candidate genes whose expression is associated with open chromatin regions, we screened for genes that were up-regulated in both expression and peak signal [7]. The comparison between E70 and E45 yielded the fewest genes, with 6 down-regulated and 28 up-regulated at E70. In the E100 vs. E70 comparison, 62 up-regulated and 50 down-regulated genes were identified at E100. In contrast, the E100 vs. E45 comparison revealed 537 up-regulated and 186 down-regulated genes at E100 (Figure 6A). Four genes selected for validation of the integrative analysis results included those related to mitosis (PTN), energy metabolism (PPP1R3C), and muscle contraction (LMOD2 and RCSD1) (Figure 6B). GO and KEGG pathway enrichment analyses were conducted on these overlapping genes with consistent expression profiles (Supplement 8). The analysis identified 40 significant KEGG pathways, including those involved in fatty acid degradation, fatty acid metabolism, AMPK signaling pathway, and PPAR signaling pathway, which were associated with the up-regulated genes at E100 compared to E45 (Figure 6C). These pathways were pertinent to adipogenesis and metabolism of fat and energy. Taking PGAM2 as a representative case, this study shed light on the potential mechanisms by which chromatin accessibility modulated gene expression. PGAM2 plays a crucial role in myoblast fusion and terminal differentiation [12]. The analysis revealed a greater abundance of ATAC-seq peaks upstream of PGAM2 at E100 relative to E45, alongside a marked increase in its expression level at E100 compared to E45 (Figures 7A, 7B). As myogenic transcription factors, MYF6 and MEF2A could bind to the promoter region to initiate the expression of myogenesis-related genes. The expression levels of these two genes were significantly higher at E100 than at E45 (Figure 7B). Additionally, potential binding sites for MYF6 and MEF2A were predicted within the PGAM2 promoter region (Figure 7C). Consequently, the upregulation of PGAM2 expression at E100 may be attributed to increased chromatin accessibility, which facilitates the binding of MYF6 and MEF2A to the promoter region.

## DISCUSSION

To mitigate the effects of genetic background and environmental factors, full-sibs at each developmental stage were utilized for both ATAC-seq and RNA-seq analyses within the same individuals, aiming to investigate the functional roles of ACRs on gene expression during the skeletal muscle development in Min pigs. Consistent with previous studies, ATAC-seq peaks were predominantly concentrated around the TSS region [6,13]. Among the comparisons, the largest span, E100 vs. E45, exhibited the highest number of genes corresponding to differential peaks. This observation indicates that dynamic changes in chromatin accessibility are integral to the transcriptional regulatory processes that drive gene expression necessary for skeletal muscle development at various stages. In alignment with the ATAC-seq results, the E100 vs. E45 comparison revealed the greatest number of differential genes. To further elucidate the roles of these differential genes, GO and KEGG enrichment analyses were conducted, identifying key muscle-related biological processes and pathways. Overall, genes associated with skeletal muscle contraction, skeletal muscle fiber development, and energy metabolism were up-regulated, while genes related to the cell cycle and proliferation were down-regulated as fetal development progressed.

During the embryonic and fetal periods, skeletal muscle development follows a complex, multistep process that includes myogenic lineage commitment, myogenic progenitor cell migration, myoblast proliferation, fusion, terminal differentiation into myotubes, and specialization into various muscle fiber types [14]. Genes associated with the mitotic cell cycle, cell division, and proliferation were up-regulated at E45 and E70 compared to E100. Specifically, *TUBB* and *STMN1* were up-regulated at E45 and E70 relative to E100, while *PLK1* was more highly expressed at E45 compared to E70. These genes were involved in cell cycle regulation, the arrest of which was the initial event toward differentiation. The high expression of these genes indicated that E45 and E70 exhibited a robust proliferative capacity, which contributed to an increased number of myoblasts available for subsequent muscle differentiation. The cell cycle encompasses DNA synthesis, mitosis, G1, and G2 phases. Genes associated with DNA synthesis were up-regulated at E45 (e.g., *MCM2*, *MCM5*, *MCM4*, *CDC6*, *CCNE2*, *MCM3*, *MCM6*, *ORC1*, *MCM10*, and *CDC45*) and E70 (e.g., *MCM2*, *PRIM2*, *MCM4*, *POLE*, *MCM3*, *DNA2*, and *POLE2*) compared to E100. The WNT/β-catenin pathway is crucial for myogenic proliferation during both development and regeneration [15]. Ras and Rap1 are members of the Ras family of GTPases [16]. Previous study shows that prenylated RAP1 GTPase promotes myoblast proliferation and inhibits differentiation [17]. Genes related to the Wnt, Ras, and Rap1 signaling pathways were up-regulated at E45 compared to E100, supporting enhanced proliferation at E45. Enrichment analyses of up-regulated genes at E70 vs. E45, E100 vs. E45, and E100 vs. E70 highlighted biological processes related to skeletal muscle fiber development, skeletal muscle cell differentiation, and myoblast fusion, suggesting an increased capacity of muscle formation during fetal development. Type II muscle fiber-related genes (*MYH1*, *MYH2*, and *MYH4*) were more abundant at E70 and E100 than at E45, indicating the emergence of type II muscle fibers during these periods. Correspondingly, muscle contraction-related genes, such as *MYBPC2*, *TNNT3*, and *TMOD4*, were up-regulated in comparisons between E70 and E45, E100 and E45, and E100 and E70. Energy metabolism is essential for myogenic differentiation, muscle maintenance, and contraction. GO analysis revealed that the tricarboxylic acid cycle was enriched with up-regulated genes (*SDHB*, *SDHC*, *OGDH*, *SDHA*, *SDHD*, *MDH2*, *IDH2*, *ACO2*, *PDHA1*, and *SUCLG1*) at E100 compared to E70 and E45, reflecting significant energy demands of the skeletal muscle during this period. ATP was generated in the mitochondria through oxidative phosphorylation [18]. Genes associated with mitochondrial electron transport, mitochondrial respiratory chain complex I assembly, oxidative phosphorylation, and ATP synthesis coupled electron transport were also up-regulated at E100. The up-regulation of genes related to the PPAR and AMPK signaling pathways at E100 indicates their roles in regulating fatty acid oxidation and glucose utilization [19,20], as well as in muscle fiber type conversion [21,22]. Overall, these differential expression results demonstrated a decrease in myoblast proliferation and an increase in muscle fiber formation and energy metabolism as fetal development progresses.

The hierarchical regulation of myogenesis is critically dependent on myogenic transcription factors. Gene expression data were clustered to elucidate the dynamic expression patterns of these regulators across different fetal stages. *PAX7*, *KI67*, and *MYF5* were grouped into cluster 2, demonstrating a consistent decline in expression from E45 to E100. *PAX7* plays a pivotal role in modulating the myoblast cell cycle and proliferation [23], *MYF5*, a key myogenic regulatory factor (MRF), supports myoblast proliferation and maintains their myogenic specification [14], and *KI67* serves as a marker for cell cycle entry and myoblast proliferative capacity [24]. The observed decline in these factors suggests a reduced proliferative capacity in myoblasts as development progresses. In contrast, *MYOD*, *MYOG*, and *MEF2C* were classified into cluster 3, characterized by peak expression at E70, whereas *MYF6*, *MEF2A*, and *MEF2B* were placed in cluster 1, showing high expression levels at both E70 and E100. *MYOD*, *MYOG*, and *MYF6*, also belong to MRFs and are involved in myogenic differentiation [25]. In addition to MRFs, the myocyte enhancer factor (*MEF2*) family members are crucial for activating muscle-specific genes during the initial stage of differentiation [26]. The upregulation of MRFs and *MEF2* factors at E70 underscores this period as critical for myoblast differentiation. Time series expression analysis further delineated muscle fiber formation. *MYH3* is primarily expressed in the embryonic period [25]. Primary muscle fibers are initially formed during fetal muscle development and are mainly composed of *MYH7* (type I oxidative slow twitch fibers). Then secondary muscle fibers, expressing type II glycolytic fast-twitch fibers (*MYH1*, *MYH2*, and *MYH4*), emerge around primary muscle fibers [27]. In this study, *MYH3*, belonging to cluster 6, was highly expressed at E45 and E70 but sharply declined at E100; *MYH7*, from cluster 1, was minimally expressed at E45 but maintained high levels from E70; *MYH4* (IIb) showed a continuous increase in cluster 4, while *MYH2* (IIa) and *MYH1* (IIx) reached peak expression at E100 in cluster 9. These results suggest that in Min pigs, *MYH3* expression peaks early in fetal development, type I fibers predominate and remain stable from E70, and type II fibers reach their highest content at E100, reflecting the progressive development pattern of muscle fibers.

An integrated analysis of ATAC-seq and RNA-seq data was conducted to evaluate the impact of chromatin accessibility on gene expression. Only a subset of genes demonstrated increased expression coinciding with open chromatin regions, suggesting that transcriptional regulation is a complex process potentially influenced by additional mechanisms such as histone modification, DNA methylation, and post-transcriptional modifications [28]. Furthermore, differences in library construction methodologies between ATAC-seq and RNA-seq may introduce varying biases in reads detection. GO enrichment analysis was performed to elucidate the biological functions of genes intersecting across the three data sets. Genes with elevated ACRs at E100 compared to E70 were predominantly associated with energy metabolisms, including the glucagon signaling pathway, positive regulation of ATP biosynthetic process, and insulin signaling pathway. In the E45 vs. E100 comparison, genes with up-regulated ACRs at E45 were linked to the Ras and Rap1 signaling pathways. Conversely, genes with up-regulated ACRs at E100 were primarily involved in sarcomere organization, muscle contraction, fatty acid beta-oxidation, glucagon signaling pathway, PPAR signaling pathway, AMPK signaling pathway, insulin signaling pathway, and thyroid hormone signaling pathway. These findings suggest that chromatin accessibility significantly influences myoblast proliferation in the early stage and their subsequent differentiation during the fetal development of Min pigs.

## CONCLUSION

In summary, the molecular mechanisms underlying the development of fetal skeletal muscle in Min pigs—a Chinese indigenous breed—were explored using ATAC-seq and RNA-seq. RNA-seq analysis revealed a reduction in myoblast proliferation alongside an increase in muscle fiber formation and energy metabolism as the fetus progressed from E45 to E100. Furthermore, type I muscle fibers emerged first, followed by type II fibers. The differential analysis from ATAC-seq exhibited a parallel pattern to RNA-seq, with an increasing number of differential genes over time. Integration of ATAC-seq and RNA-seq data indicated that chromatin accessibility influenced both early myoblast proliferation and later-stage muscle metabolism. These insights contribute to a deeper understanding of dynamic transcriptional regulation throughout fetal skeletal muscle development.

## SUPPLEMENTARY MATERIAL

**Supplement 1**. Statistics of ATAC-seq data.

**Supplement 2**. Heatmaps for differential peaks at E45, E70, and E100.

**Supplement 3**. Statistics of RNA-seq data.

**Supplement 4**. GO and KEGG enrichment analysis of differently expressed genes.

**Supplement 5**. Gene list for Figure 4B.

**Supplement 6**. Heatmap for gene clustering.

**Supplement 7**. GO and KEGG enrichment analysis of clustering genes.

**Supplement 8**. GO and KEGG enrichment analysis of overlapping genes.

## References

[1] Le Grand F, Rudnicki MA (2007). Skeletal muscle satellite cells and adult myogenesis. Curr Opin Cell Biol.

[2] Picard B, Berri C, Lefaucheur L, Molette C, Sayd T, Terlouw C (2010). Skeletal muscle proteomics in livestock production. Brief Funct Genomics.

[3] Rudar M, Fiorotto ML, Davis TA (2019). Regulation of muscle growth in early postnatal life in a swine model. Annu Rev Anim Biosci.

[4] Morrison O, Thakur J (2021). Molecular complexes at euchromatin, heterochromatin and centromeric chromatin. Int J Mol Sci.

[5] Grandi FC, Modi H, Kampman L, Corces MR (2022). Chromatin accessibility profiling by ATAC-seq. Nat Protoc.

[6] Yue J, Hou X, Liu X (2021). The landscape of chromatin accessibility in skeletal muscle during embryonic development in pigs. J Anim Sci Biotechnol.

[7] Feng L, Si J, Yue J (2023). The landscape of accessible chromatin and developmental transcriptome maps reveal a genetic mechanism of skeletal muscle development in pigs. Int J Mol Sci.

[8] Cai S, Hu B, Wang X (2023). Integrative single-cell RNA-seq and ATAC-seq analysis of myogenic differentiation in pig. BMC Biol.

[9] Salavati M, Woolley SA, Cortés Araya Y (2022). Profiling of open chromatin in developing pig (Sus scrofa) muscle to identify regulatory regions. G3 Genes Genomes Genet.

[10] Zhao X, Mo D, Li A (2011). Comparative analyses by sequencing of transcriptomes during skeletal muscle development between pig breeds differing in muscle growth rate and fatness. PLOS ONE.

[11] Vrljicak P, Lucas ES, Lansdowne L (2018). Analysis of chromatin accessibility in decidualizing human endometrial stromal cells. FASEB J.

[12] Zhang Y, Beketaev I, Ma Y, Wang J (2022). Sumoylation-deficient phosphoglycerate mutase 2 impairs myogenic differentiation. Front Cell Dev Biol.

[13] Su Y, He S, Chen Q (2024). Integrative ATAC-seq and RNA-seq analysis of myogenic differentiation of ovine skeletal muscle satellite cell. Genomics.

[14] Parker MH, Seale P, Rudnicki MA (2003). Looking back to the embryo: defining transcriptional networks in adult myogenesis. Nat Rev Genet.

[15] Suzuki A, Scruggs A, Iwata J (2015). The temporal specific role of WNT/β-catenin signaling during myogenesis. J Nat Sci.

[16] Cantrell DA (2003). GTPases and T cell activation. Immunol Rev.

[17] Jaśkiewicz A, Pająk B, Litwiniuk A, Urbańska K, Orzechowski A (2018). Geranylgeraniol prevents statin-dependent myotoxicity in C2C12 muscle cells through RAP1 GTPase prenylation and cytoprotective autophagy. Oxid Med Cell Longev.

[18] Chen MM, Li Y, Deng SL, Zhao Y, Lian ZX, Yu K (2022). Mitochondrial function and reactive oxygen/nitrogen species in skeletal muscle. Front Cell Dev Biol.

[19] Barger PM, Kelly DP (2000). PPAR signaling in the control of cardiac energy metabolism. Trends Cardiovasc Med.

[20] Smith AC, Bruce CR, Dyck DJ (2005). AMP kinase activation with AICAR simultaneously increases fatty acid and glucose oxidation in resting rat soleus muscle. J Physiol.

[21] Luquet S, Lopez-Soriano J, Holst D (2003). Peroxisome proliferator-activated receptor δ controls muscle development and oxidative capability. FASEB J.

[22] Li P, Zhang S, Song H (2021). Naringin promotes skeletal muscle fiber remodeling by the AdipoR1-APPL1-AMPK signaling pathway. J Agric Food Chem.

[23] Hernández-Hernández O, Ávila-Avilés RD, Hernández-Hernández JM (2020). Chromatin landscape during skeletal muscle differentiation. Front Genet.

[24] Rozance PJ, Wesolowski SR, Jonker SS, Brown LD (2021). Anemic hypoxemia reduces myoblast proliferation and muscle growth in late-gestation fetal sheep. Am J Physiol Regul Integr Comp Physiol.

[25] Chal J, Pourquié O (2017). Making muscle: skeletal myogenesis in vivo and in vitro. Development.

[26] Molkentin JD, Olson EN (1996). Defining the regulatory networks for muscle development. Curr Opin Genet Dev.

[27] Biressi S, Molinaro M, Cossu G (2007). Cellular heterogeneity during vertebrate skeletal muscle development. Dev Biol.

[28] Bai J, Lin Y, Zhang J (2023). Profiling of chromatin accessibility in pigs across multiple tissues and developmental stages. Int J Mol Sci.

