# Supplementary material for: Dynamic changes in chromatin accessibility and gene expression involved in fetal myogenesis of Min pigs
Source: Anim Biosci. 2025 May 12;38(11):2525–36. doi: 10.5713/ab.25.0034 (PMC12580940; doi:10.5713/ab.25.0034)
Supplement: Supplementary file 1 [file ab-25-0034-supplementary-1.pdf]

Supplement 1. Statistics of ATAC-seq data.

Sample ID	Clean reads	Total mapped reads	Mapped rate %
M45_F_1	186605822	167754473	89.90
M45_F_2	188702834	169276128	89.71
M45_M_1	192895365	173511109	89.95
M45_M_2	185365029	164509273	88.75
M70_F_1	186514178	166306836	89.17
M70_F_2	187824062	166365899	88.58
M70_M_1	183279408	149116008	81.36
M70_M_2	187742276	150001579	79.90
M100_F_1	184662423	154546511	83.69
M100_F_2	186349474	158916832	85.28
M100_M_1	182837539	154149811	84.31
M100_M_2	187592727	159269054	84.90
