# Supplementary figures and images for: Dynamic changes in chromatin accessibility and gene expression involved in fetal myogenesis of Min pigs
Source: Anim Biosci. 2025 May 12;38(11):2525–36. doi: 10.5713/ab.25.0034 (PMC12580940; doi:10.5713/ab.25.0034)

Supplement 2. Heatmaps for differential peaks at E45, E70, and E100.

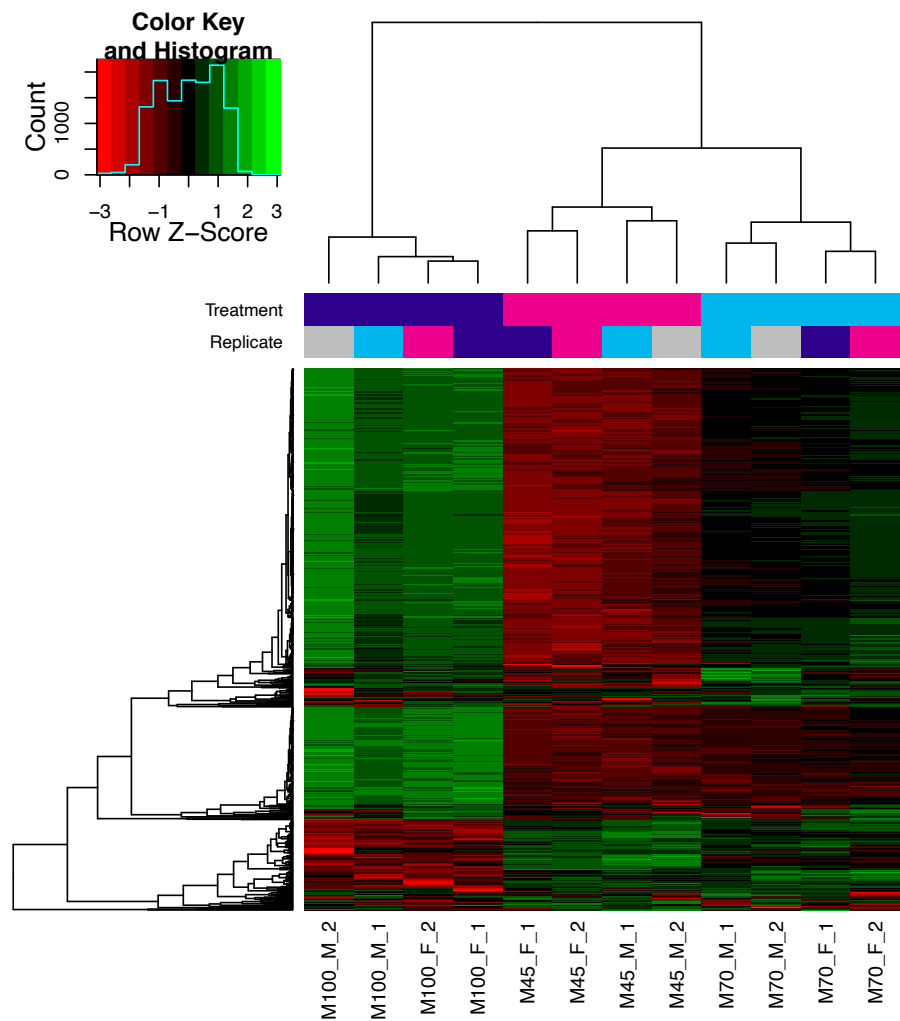

Supplement: Supplementary file 2 [file ab-25-0034-supplementary-2.pdf]

## Supplement 6. Heatmap for gene clustering.

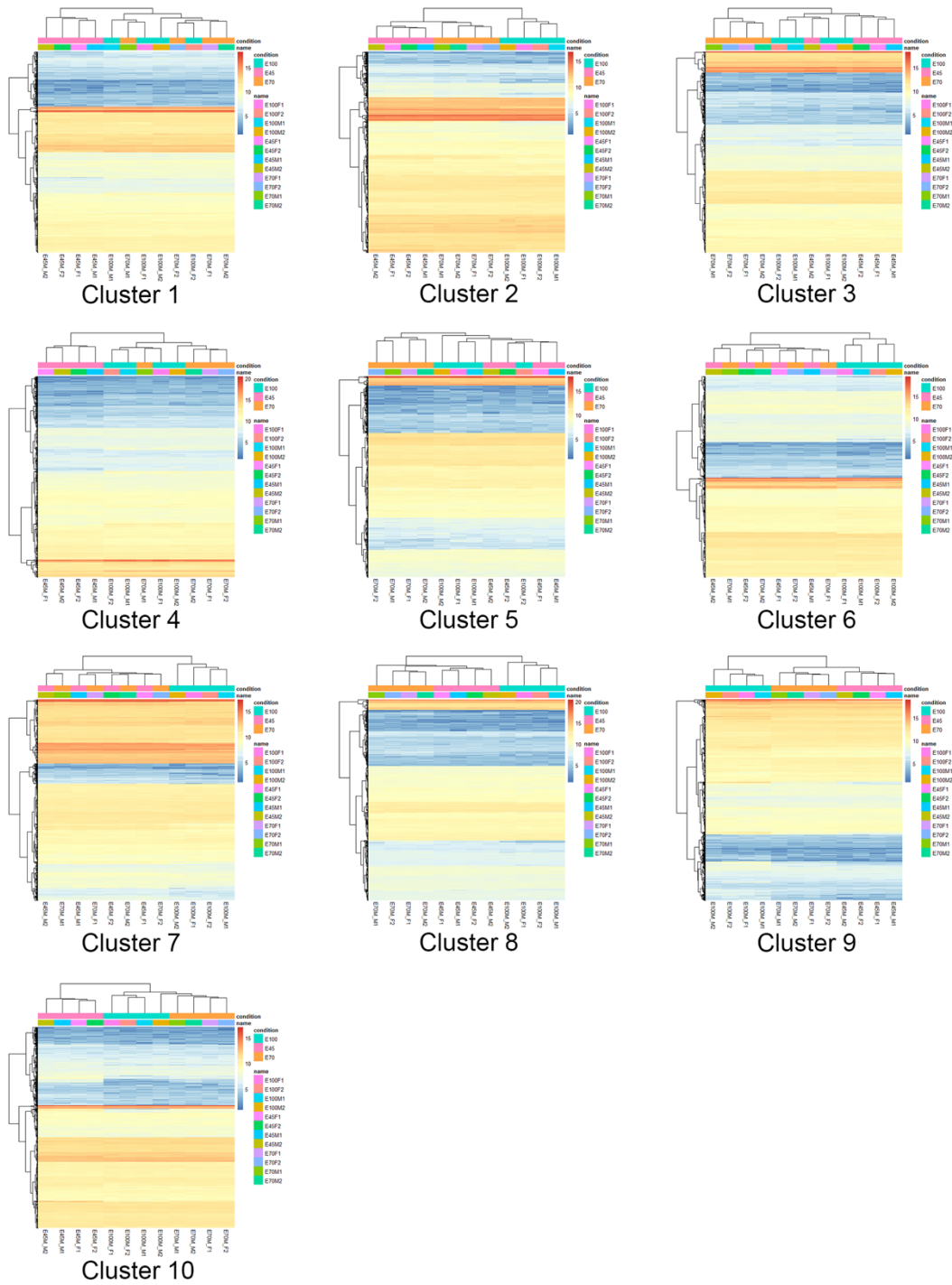

Supplement: Supplementary file 6 [file ab-25-0034-supplementary-6.pdf]
