# Supplementary material for: Dynamic changes in chromatin accessibility and gene expression involved in fetal myogenesis of Min pigs
Source: Anim Biosci. 2025 May 12;38(11):2525–36. doi: 10.5713/ab.25.0034 (PMC12580940; doi:10.5713/ab.25.0034)
Supplement: Supplementary file 3 [file ab-25-0034-supplementary-3.pdf]

Supplement 3. Statistics of RNA-seq data.

Sample ID	Clean reads	Total mapped reads	Mapped rate %
M45_F_1	30276072	26320364	94.08
M45_F_2	28194507	24448658	94.02
M45_M_1	28731135	25203026	94.52
M45_M_2	21599709	18911914	94.50
M70_F_1	25429858	22079512	94.27
M70_F_2	31553179	27521646	94.55
M70_M_1	33388830	28867844	93.97
M70_M_2	29270682	25390440	94.22
M100_F_1	32096896	27642314	94.11
M100_F_2	27373950	23707344	94.15
M100_M_1	35267659	30464319	94.16
M100_M_2	28095315	24478801	94.33
