# Supplementary material for: Dynamic changes in chromatin accessibility and gene expression involved in fetal myogenesis of Min pigs
Source: Anim Biosci. 2025 May 12;38(11):2525–36. doi: 10.5713/ab.25.0034 (PMC12580940; doi:10.5713/ab.25.0034)
Supplement: Supplementary file 4 [file ab-25-0034-supplementary-4.pdf]

Supplement 4. GO and KEGG enrichment analysis of differently expressed genes.

E45＞E70

Category	Term	PValue
GOTERM_BP_DIRECT	GO:0045668~negative regulation of osteoblast differentiation	0.001656387
GOTERM_BP_DIRECT	GO:0035331~negative regulation of hippo signaling	0.002665621
GOTERM_BP_DIRECT	GO:0007157~heterophilic cell-cell adhesion via plasma membrane cell adhesion molecules	0.003262977
GOTERM_BP_DIRECT	GO:1903238~positive regulation of leukocyte tethering or rolling	0.009677665
GOTERM_BP_DIRECT	GO:0033689~negative regulation of osteoblast proliferation	0.012714522
GOTERM_BP_DIRECT	GO:0007417~central nervous system development	0.013975064
GOTERM_BP_DIRECT	GO:0001974~blood vessel remodeling	0.015743058
GOTERM_BP_DIRECT	GO:0035556~intracellular signal transduction	0.01766468
GOTERM_BP_DIRECT	GO:0007416~synapse assembly	0.021664921
GOTERM_BP_DIRECT	GO:0019722~calcium-mediated signaling	0.023461078
GOTERM_BP_DIRECT	GO:0010820~positive regulation of T cell chemotaxis	0.023897338
GOTERM_BP_DIRECT	GO:0007193~adenylate cyclase-inhibiting G-protein coupled receptor signaling pathway	0.026198579
GOTERM_BP_DIRECT	GO:0001558~regulation of cell growth	0.026198579
GOTERM_BP_DIRECT	GO:0007155~cell adhesion	0.027287319
GOTERM_BP_DIRECT	GO:0048514~blood vessel morphogenesis	0.028259863
GOTERM_BP_DIRECT	GO:0001649~osteoblast differentiation	0.032141225
GOTERM_BP_DIRECT	GO:0007165~signal transduction	0.034098744
GOTERM_BP_DIRECT	GO:0098962~regulation of postsynaptic neurotransmitter receptor activity	0.037842762
GOTERM_BP_DIRECT	GO:0045197~establishment or maintenance of epithelial cell apical/basal polarity	0.037842762
GOTERM_BP_DIRECT	GO:0051968~positive regulation of synaptic transmission, glutamatergic	0.037842762
GOTERM_BP_DIRECT	GO:0007269~neurotransmitter secretion	0.037842762
GOTERM_BP_DIRECT	GO:0000086~G2/M transition of mitotic cell cycle	0.039568323
GOTERM_BP_DIRECT	GO:0050905~neuromuscular process	0.043033516
GOTERM_BP_DIRECT	GO:0098609~cell-cell adhesion	0.043182304
GOTERM_BP_DIRECT	GO:0035705~T-helper 17 cell chemotaxis	0.04412813
GOTERM_BP_DIRECT	GO:2000451~positive regulation of CD8-positive, alpha-beta T cell extravasation	0.04412813
GOTERM_BP_DIRECT	GO:2000464~positive regulation of astrocyte chemotaxis	0.04412813
GOTERM_BP_DIRECT	GO:0061756~leukocyte adhesion to vascular endothelial cell	0.04412813
GOTERM_BP_DIRECT	GO:2000473~positive regulation of hematopoietic stem cell migration	0.04412813

GOTERM_BP_DIRECT	GO:0007600~sensory perception	0.04412813
GOTERM_BP_DIRECT	GO:0051388~positive regulation of neurotrophin TRK receptor signaling pathway	0.04412813
GOTERM_BP_DIRECT	GO:0043310~negative regulation of eosinophil degranulation	0.04412813
GOTERM_BP_DIRECT	GO:0035696~monocyte extravasation	0.04412813
GOTERM_BP_DIRECT	GO:0042474~middle ear morphogenesis	0.048471584
GOTERM_CC_DIRECT	GO:0098978~glutamatergic synapse	3.15E-05
GOTERM_CC_DIRECT	GO:0005887~integral component of plasma membrane	1.09E-04
GOTERM_CC_DIRECT	GO:0098982~GABA-ergic synapse	1.93E-04
GOTERM_CC_DIRECT	GO:0099060~integral component of postsynaptic specialization membrane	2.35E-04
GOTERM_CC_DIRECT	GO:0014069~postsynaptic density	0.001669048
GOTERM_CC_DIRECT	GO:0045202~synapse	0.002980884
GOTERM_CC_DIRECT	GO:0005576~extracellular region	0.003606877
GOTERM_CC_DIRECT	GO:0043235~receptor complex	0.003890722
GOTERM_CC_DIRECT	GO:0009986~cell surface	0.004939519
GOTERM_CC_DIRECT	GO:0005886~plasma membrane	0.008852336
GOTERM_CC_DIRECT	GO:0043197~dendritic spine	0.009796433
GOTERM_CC_DIRECT	GO:0043005~neuron projection	0.013296605
GOTERM_CC_DIRECT	GO:0045211~postsynaptic membrane	0.014156748
GOTERM_CC_DIRECT	GO:0016323~basolateral plasma membrane	0.020366231
GOTERM_CC_DIRECT	GO:0005911~cell-cell junction	0.022426034
GOTERM_CC_DIRECT	GO:0009897~external side of plasma membrane	0.023126331
GOTERM_CC_DIRECT	GO:0098793~presynapse	0.032790712
GOTERM_CC_DIRECT	GO:0030424~axon	0.043196727
GOTERM_CC_DIRECT	GO:0043025~neuronal cell body	0.047503203
GOTERM_CC_DIRECT	GO:0005912~adherens junction	0.049537432
GOTERM_MF_DIRECT	GO:0005509~calcium ion binding	4.40E-05
GOTERM_MF_DIRECT	GO:0022857~transmembrane transporter activity	0.003137462
GOTERM_MF_DIRECT	GO:0005102~receptor binding	0.009110111
GOTERM_MF_DIRECT	GO:0019957~C-C chemokine binding	0.00945273
GOTERM_MF_DIRECT	GO:0004888~transmembrane signaling receptor activity	0.010753539
GOTERM_MF_DIRECT	GO:0016493~C-C chemokine receptor activity	0.010776087
GOTERM_MF_DIRECT	GO:0019900~kinase binding	0.022675309
GOTERM_MF_DIRECT	GO:0038023~signaling receptor activity	0.025257601
GOTERM_MF_DIRECT	GO:0034632~retinol transporter activity	0.041937964
GOTERM_MF_DIRECT	GO:0035715~chemokine (C-C motif) ligand 2 binding	0.041937964
GOTERM_MF_DIRECT	GO:0045569~TRAIL binding	0.041937964
GOTERM_MF_DIRECT	GO:0035716~chemokine (C-C motif) ligand 12 binding	0.041937964
GOTERM_MF_DIRECT	GO:0008013~beta-catenin binding	0.048946917
KEGG_PATHWAY	ssc04080:Neuroactive ligand-receptor interaction	4.81E-05
KEGG_PATHWAY	ssc04814:Motor proteins	0.001299494
KEGG_PATHWAY	ssc04020:Calcium signaling pathway	0.005493285

KEGG_PATHWAY	ssc04060:Cytokine-cytokine receptor interaction	0.009002655
KEGG_PATHWAY	ssc04015:Rap1 signaling pathway	0.010797458
KEGG_PATHWAY	ssc05214:Glioma	0.013923255
KEGG_PATHWAY	ssc04061:Viral protein interaction with cytokine and cytokine receptor	0.028073414
KEGG_PATHWAY	ssc04514:Cell adhesion molecules	0.036789226
KEGG_PATHWAY	ssc04924:Renin secretion	0.049865902

E45＞E100

Category	Term	PValue
GOTERM_BP_DIRECT	GO:0051301~cell division	1.23E-13
GOTERM_BP_DIRECT	GO:0007059~chromosome segregation	2.39E-09
GOTERM_BP_DIRECT	GO:0000278~mitotic cell cycle	3.40E-09
GOTERM_BP_DIRECT	GO:0006335~DNA replication-dependent nucleosome assembly	5.09E-09
GOTERM_BP_DIRECT	GO:0006268~DNA unwinding involved in DNA replication	1.12E-07
GOTERM_BP_DIRECT	GO:0007049~cell cycle	1.96E-07
GOTERM_BP_DIRECT	GO:0007018~microtubule-based movement	4.31E-07
GOTERM_BP_DIRECT	GO:0007052~mitotic spindle organization	1.11E-06
GOTERM_BP_DIRECT	GO:0007094~mitotic spindle assembly checkpoint	3.29E-06
GOTERM_BP_DIRECT	GO:0006270~DNA replication initiation	3.38E-06
GOTERM_BP_DIRECT	GO:0000070~mitotic sister chromatid segregation	5.56E-06
GOTERM_BP_DIRECT	GO:0006336~DNA replication-independent nucleosome assembly	1.13E-05
GOTERM_BP_DIRECT	GO:0036297~interstrand cross-link repair	2.21E-05
GOTERM_BP_DIRECT	GO:0000727~double-strand break repair via break-induced replication	2.77E-05
GOTERM_BP_DIRECT	GO:0000281~mitotic cytokinesis	3.99E-05
GOTERM_BP_DIRECT	GO:0000086~G2/M transition of mitotic cell cycle	8.58E-05
GOTERM_BP_DIRECT	GO:0045653~negative regulation of megakaryocyte differentiation	1.31E-04
GOTERM_BP_DIRECT	GO:0000132~establishment of mitotic spindle orientation	1.50E-04
GOTERM_BP_DIRECT	GO:0006695~cholesterol biosynthetic process	2.12E-04
GOTERM_BP_DIRECT	GO:0007411~axon guidance	2.20E-04
GOTERM_BP_DIRECT	GO:0051382~kinetochore assembly	2.42E-04
GOTERM_BP_DIRECT	GO:0045143~homologous chromosome segregation	2.60E-04
GOTERM_BP_DIRECT	GO:0051256~mitotic spindle midzone assembly	2.60E-04
GOTERM_BP_DIRECT	GO:0099560~synaptic membrane adhesion	3.19E-04
GOTERM_BP_DIRECT	GO:0030199~collagen fibril organization	5.39E-04
GOTERM_BP_DIRECT	GO:0051965~positive regulation of synapse assembly	7.11E-04
GOTERM_BP_DIRECT	GO:0050804~modulation of synaptic transmission	7.52E-04
GOTERM_BP_DIRECT	GO:0000226~microtubule cytoskeleton organization	7.97E-04

GOTERM_BP_DIRECT	GO:1904146~positive regulation of meiotic cell cycle process involved in oocyte maturation	8.36E-04
GOTERM_BP_DIRECT	GO:0007019~microtubule depolymerization	8.36E-04
GOTERM_BP_DIRECT	GO:0044772~mitotic cell cycle phase transition	0.001008246
GOTERM_BP_DIRECT	GO:0007051~spindle organization	0.001424065
GOTERM_BP_DIRECT	GO:0060371~regulation of atrial cardiac muscle cell membrane depolarization	0.001575575
GOTERM_BP_DIRECT	GO:0006281~DNA repair	0.001667223
GOTERM_BP_DIRECT	GO:0007157~heterophilic cell-cell adhesion via plasma membrane cell adhesion molecules	0.001978802
GOTERM_BP_DIRECT	GO:1900264~positive regulation of DNA-directed DNA polymerase activity	0.002268269
GOTERM_BP_DIRECT	GO:1903934~positive regulation of DNA primase activity	0.002416238
GOTERM_BP_DIRECT	GO:0007399~nervous system development	0.002983676
GOTERM_BP_DIRECT	GO:0035556~intracellular signal transduction	0.003053326
GOTERM_BP_DIRECT	GO:0007409~axonogenesis	0.003077613
GOTERM_BP_DIRECT	GO:0001578~microtubule bundle formation	0.003312436
GOTERM_BP_DIRECT	GO:0034501~protein localization to kinetochore	0.003425381
GOTERM_BP_DIRECT	GO:0006334~nucleosome assembly	0.004714588
GOTERM_BP_DIRECT	GO:0021987~cerebral cortex development	0.004911241
GOTERM_BP_DIRECT	GO:0060348~bone development	0.004911241
GOTERM_BP_DIRECT	GO:0016126~sterol biosynthetic process	0.00492421
GOTERM_BP_DIRECT	GO:0007520~myoblast fusion	0.004952631
GOTERM_BP_DIRECT	GO:0007224~smoothened signaling pathway	0.004988879
GOTERM_BP_DIRECT	GO:0048477~oogenesis	0.005557968
GOTERM_BP_DIRECT	GO:0010389~regulation of G2/M transition of mitotic cell cycle	0.005651334
GOTERM_BP_DIRECT	GO:0000082~G1/S transition of mitotic cell cycle	0.006000934
GOTERM_BP_DIRECT	GO:0090307~mitotic spindle assembly	0.006000934
GOTERM_BP_DIRECT	GO:0090090~negative regulation of canonical Wnt signaling pathway	0.006342074
GOTERM_BP_DIRECT	GO:0007017~microtubule-based process	0.006761355
GOTERM_BP_DIRECT	GO:0060070~canonical Wnt signaling pathway	0.007074379
GOTERM_BP_DIRECT	GO:0008284~positive regulation of cell proliferation	0.007289825
GOTERM_BP_DIRECT	GO:0007080~mitotic metaphase plate congression	0.008355539
GOTERM_BP_DIRECT	GO:0001707~mesoderm formation	0.009215469
GOTERM_BP_DIRECT	GO:0031297~replication fork processing	0.009215469
GOTERM_BP_DIRECT	GO:0014032~neural crest cell development	0.009340804
GOTERM_BP_DIRECT	GO:0010976~positive regulation of neuron projection development	0.0094982
GOTERM_BP_DIRECT	GO:0007165~signal transduction	0.009963067
GOTERM_BP_DIRECT	GO:0051988~regulation of attachment of spindle microtubules to kinetochore	0.010577704
GOTERM_BP_DIRECT	GO:0060174~limb bud formation	0.011207022

GOTERM_BP_DIRECT	GO:0007043~cell-cell junction assembly	0.011574044
GOTERM_BP_DIRECT	GO:0007156~homophilic cell adhesion via plasma membrane adhesion molecules	0.013303876
GOTERM_BP_DIRECT	GO:0006352~DNA-templated transcription, initiation	0.014327913
GOTERM_BP_DIRECT	GO:0001654~eye development	0.014327913
GOTERM_BP_DIRECT	GO:0098609~cell-cell adhesion	0.015276722
GOTERM_BP_DIRECT	GO:0007267~cell-cell signaling	0.015591122
GOTERM_BP_DIRECT	GO:0071773~cellular response to BMP stimulus	0.015676398
GOTERM_BP_DIRECT	GO:0048514~blood vessel morphogenesis	0.015676398
GOTERM_BP_DIRECT	GO:0031110~regulation of microtubule polymerization or depolymerization	0.015676398
GOTERM_BP_DIRECT	GO:0016266~O-glycan processing	0.017329301
GOTERM_BP_DIRECT	GO:0018105~peptidyl-serine phosphorylation	0.017374157
GOTERM_BP_DIRECT	GO:0030900~forebrain development	0.017506517
GOTERM_BP_DIRECT	GO:0010634~positive regulation of epithelial cell migration	0.017506517
GOTERM_BP_DIRECT	GO:0048146~positive regulation of fibroblast proliferation	0.017550987
GOTERM_BP_DIRECT	GO:0030198~extracellular matrix organization	0.020238051
GOTERM_BP_DIRECT	GO:0006302~double-strand break repair	0.020333792
GOTERM_BP_DIRECT	GO:0050801~ion homeostasis	0.021121294
GOTERM_BP_DIRECT	GO:0086010~membrane depolarization during action potential	0.021121294
GOTERM_BP_DIRECT	GO:0019240~citrulline biosynthetic process	0.021140194
GOTERM_BP_DIRECT	GO:0016477~cell migration	0.022691439
GOTERM_BP_DIRECT	GO:0019228~neuronal action potential	0.024832836
GOTERM_BP_DIRECT	GO:0016339~calcium-dependent cell-cell adhesion via plasma membrane cell adhesion molecules	0.024832836
GOTERM_BP_DIRECT	GO:0045880~positive regulation of smoothened signaling pathway	0.025244485
GOTERM_BP_DIRECT	GO:0007416~synapse assembly	0.025244485
GOTERM_BP_DIRECT	GO:0007169~transmembrane receptor protein tyrosine kinase signaling pathway	0.025452707
GOTERM_BP_DIRECT	GO:0051298~centrosome duplication	0.025965445
GOTERM_BP_DIRECT	GO:0048678~response to axon injury	0.025965445
GOTERM_BP_DIRECT	GO:0032331~negative regulation of chondrocyte differentiation	0.027588759
GOTERM_BP_DIRECT	GO:0098962~regulation of postsynaptic neurotransmitter receptor activity	0.027588759
GOTERM_BP_DIRECT	GO:0030336~negative regulation of cell migration	0.027875044
GOTERM_BP_DIRECT	GO:0048013~ephrin receptor signaling pathway	0.030336546
GOTERM_BP_DIRECT	GO:1905606~regulation of presynapse assembly	0.030336546
GOTERM_BP_DIRECT	GO:0007601~visual perception	0.033534798
GOTERM_BP_DIRECT	GO:0001558~regulation of cell growth	0.034974283

GOTERM_BP_DIRECT	GO:0090263~positive regulation of canonical Wnt signaling pathway	0.035094124
GOTERM_BP_DIRECT	GO:0001502~cartilage condensation	0.035109624
GOTERM_BP_DIRECT	GO:0006541~glutamine metabolic process	0.035109624
GOTERM_BP_DIRECT	GO:0071625~vocalization behavior	0.036486063
GOTERM_BP_DIRECT	GO:0008299~isoprenoid biosynthetic process	0.036486063
GOTERM_BP_DIRECT	GO:0031115~negative regulation of microtubule polymerization	0.036486063
GOTERM_BP_DIRECT	GO:0010971~positive regulation of G2/M transition of mitotic cell cycle	0.036548628
GOTERM_BP_DIRECT	GO:0050679~positive regulation of epithelial cell proliferation	0.037214565
GOTERM_BP_DIRECT	GO:0043401~steroid hormone mediated signaling pathway	0.03986415
GOTERM_BP_DIRECT	GO:0048702~embryonic neurocranium morphogenesis	0.03986415
GOTERM_BP_DIRECT	GO:0048630~skeletal muscle tissue growth	0.03986415
GOTERM_BP_DIRECT	GO:0000076~DNA replication checkpoint	0.03986415
GOTERM_BP_DIRECT	GO:0007171~activation of transmembrane receptor protein tyrosine kinase activity	0.03986415
GOTERM_BP_DIRECT	GO:0034080~CENP-A containing nucleosome assembly	0.03986415
GOTERM_BP_DIRECT	GO:0001649~osteoblast differentiation	0.040655098
GOTERM_BP_DIRECT	GO:0009887~animal organ morphogenesis	0.042945803
GOTERM_BP_DIRECT	GO:0048813~dendrite morphogenesis	0.043486012
GOTERM_BP_DIRECT	GO:0035082~axoneme assembly	0.04369923
GOTERM_BP_DIRECT	GO:0030534~adult behavior	0.04369923
GOTERM_BP_DIRECT	GO:0007140~male meiosis	0.04369923
GOTERM_BP_DIRECT	GO:0007417~central nervous system development	0.047119538
GOTERM_BP_DIRECT	GO:0048666~neuron development	0.047493884
GOTERM_BP_DIRECT	GO:0006228~UTP biosynthetic process	0.048844554
GOTERM_BP_DIRECT	GO:0042246~tissue regeneration	0.048844554
GOTERM_CC_DIRECT	GO:0000776~kinetochore	1.26E-09
GOTERM_CC_DIRECT	GO:0005874~microtubule	7.60E-09
GOTERM_CC_DIRECT	GO:0071162~CMG complex	6.48E-08
GOTERM_CC_DIRECT	GO:0043505~CENP-A containing nucleosome	1.56E-07
GOTERM_CC_DIRECT	GO:0000922~spindle pole	2.41E-07
GOTERM_CC_DIRECT	GO:0098978~glutamatergic synapse	1.64E-06
GOTERM_CC_DIRECT	GO:0005737~cytoplasm	4.87E-06
GOTERM_CC_DIRECT	GO:0000940~condensed chromosome outer kinetochore	2.33E-05
GOTERM_CC_DIRECT	GO:0009986~cell surface	3.40E-05
GOTERM_CC_DIRECT	GO:0005871~kinesin complex	5.78E-05
GOTERM_CC_DIRECT	GO:0045202~synapse	5.79E-05
GOTERM_CC_DIRECT	GO:0000775~chromosome, centromeric region	9.80E-05
GOTERM_CC_DIRECT	GO:0014069~postsynaptic density	1.71E-04
GOTERM_CC_DIRECT	GO:0072686~mitotic spindle	2.07E-04
GOTERM_CC_DIRECT	GO:0000228~nuclear chromosome	2.74E-04

GOTERM_CC_DIRECT	GO:0005813~centrosome	3.74E-04
GOTERM_CC_DIRECT	GO:0005654~nucleoplasm	3.89E-04
GOTERM_CC_DIRECT	GO:0001518~voltage-gated sodium channel complex	4.45E-04
GOTERM_CC_DIRECT	GO:0016323~basolateral plasma membrane	5.08E-04
GOTERM_CC_DIRECT	GO:0005876~spindle microtubule	5.29E-04
GOTERM_CC_DIRECT	GO:0005819~spindle	5.76E-04
GOTERM_CC_DIRECT	GO:0099060~integral component of postsynaptic specialization membrane	6.96E-04
GOTERM_CC_DIRECT	GO:0043005~neuron projection	7.38E-04
GOTERM_CC_DIRECT	GO:0031012~extracellular matrix	8.75E-04
GOTERM_CC_DIRECT	GO:0051233~spindle midzone	8.90E-04
GOTERM_CC_DIRECT	GO:0043240~Fanconi anaemia nuclear complex	0.001004515
GOTERM_CC_DIRECT	GO:0005814~centriole	0.00111789
GOTERM_CC_DIRECT	GO:0098685~Schaffer collateral - CA1 synapse	0.001698355
GOTERM_CC_DIRECT	GO:0031262~Ndc80 complex	0.001929941
GOTERM_CC_DIRECT	GO:0000811~GINS complex	0.001929941
GOTERM_CC_DIRECT	GO:0042555~MCM complex	0.003723777
GOTERM_CC_DIRECT	GO:0043025~neuronal cell body	0.0038032
GOTERM_CC_DIRECT	GO:0035371~microtubule plus-end	0.004979399
GOTERM_CC_DIRECT	GO:0045171~intercellular bridge	0.005011309
GOTERM_CC_DIRECT	GO:0030424~axon	0.005591202
GOTERM_CC_DIRECT	GO:0030425~dendrite	0.005932766
GOTERM_CC_DIRECT	GO:0000781~chromosome, telomeric region	0.008694487
GOTERM_CC_DIRECT	GO:0005856~cytoskeleton	0.009659272
GOTERM_CC_DIRECT	GO:0000785~chromatin	0.010515801
GOTERM_CC_DIRECT	GO:0005887~integral component of plasma membrane	0.011129058
GOTERM_CC_DIRECT	GO:0098982~GABA-ergic synapse	0.012694276
GOTERM_CC_DIRECT	GO:0031594~neuromuscular junction	0.012694276
GOTERM_CC_DIRECT	GO:0005815~microtubule organizing center	0.013090353
GOTERM_CC_DIRECT	GO:0000793~condensed chromosome	0.014741871
GOTERM_CC_DIRECT	GO:0005604~basement membrane	0.015113177
GOTERM_CC_DIRECT	GO:0030496~midbody	0.016216781
GOTERM_CC_DIRECT	GO:0031225~anchored component of membrane	0.0192309
GOTERM_CC_DIRECT	GO:0001917~photoreceptor inner segment	0.0192309
GOTERM_CC_DIRECT	GO:0005576~extracellular region	0.026733699
GOTERM_CC_DIRECT	GO:0005721~pericentric heterochromatin	0.02734676
GOTERM_CC_DIRECT	GO:0000307~cyclin-dependent protein kinase holoenzyme complex	0.029354312
GOTERM_CC_DIRECT	GO:0045211~postsynaptic membrane	0.031432974
GOTERM_CC_DIRECT	GO:0000794~condensed nuclear chromosome	0.032706
GOTERM_CC_DIRECT	GO:0030864~cortical actin cytoskeleton	0.033341814
GOTERM_CC_DIRECT	GO:0005912~adherens junction	0.033965273
GOTERM_CC_DIRECT	GO:0005588~collagen type V trimer	0.034505411
GOTERM_CC_DIRECT	GO:0097134~cyclin E1-CDK2 complex	0.034505411

GOTERM_CC_DIRECT	GO:0099061~integral component of postsynaptic density membrane	0.038674899
GOTERM_CC_DIRECT	GO:0005581~collagen trimer	0.039521944
GOTERM_CC_DIRECT	GO:1990023~mitotic spindle midzone	0.040180669
GOTERM_CC_DIRECT	GO:0033162~melanosome membrane	0.040180669
GOTERM_CC_DIRECT	GO:0015629~actin cytoskeleton	0.04168189
GOTERM_CC_DIRECT	GO:0032587~ruffle membrane	0.043877979
GOTERM_MF_DIRECT	GO:0008017~microtubule binding	4.90E-11
GOTERM_MF_DIRECT	GO:0005524~ATP binding	1.62E-09
GOTERM_MF_DIRECT	GO:0003777~microtubule motor activity	4.68E-09
GOTERM_MF_DIRECT	GO:0005509~calcium ion binding	7.26E-06
GOTERM_MF_DIRECT	GO:0004674~protein serine/threonine kinase activity	2.13E-05
GOTERM_MF_DIRECT	GO:0019901~protein kinase binding	2.32E-05
GOTERM_MF_DIRECT	GO:0005201~extracellular matrix structural constituent	6.94E-05
GOTERM_MF_DIRECT	GO:0008574~ATP-dependent microtubule motor activity, plus-end-directed	1.32E-04
GOTERM_MF_DIRECT	GO:0004712~protein serine/threonine/tyrosine kinase activity	1.58E-04
GOTERM_MF_DIRECT	GO:0005248~voltage-gated sodium channel activity	5.22E-04
GOTERM_MF_DIRECT	GO:0097110~scaffold protein binding	6.18E-04
GOTERM_MF_DIRECT	GO:0005200~structural constituent of cytoskeleton	0.001089718
GOTERM_MF_DIRECT	GO:0046875~ephrin receptor binding	0.001342806
GOTERM_MF_DIRECT	GO:0003688~DNA replication origin binding	0.002110303
GOTERM_MF_DIRECT	GO:0030246~carbohydrate binding	0.002627803
GOTERM_MF_DIRECT	GO:0008022~protein C-terminus binding	0.003495729
GOTERM_MF_DIRECT	GO:0017108~5'-flap endonuclease activity	0.004128107
GOTERM_MF_DIRECT	GO:0005102~receptor binding	0.004948082
GOTERM_MF_DIRECT	GO:0005178~integrin binding	0.005638591
GOTERM_MF_DIRECT	GO:0045296~cadherin binding	0.006833175
GOTERM_MF_DIRECT	GO:0030165~PDZ domain binding	0.006852272
GOTERM_MF_DIRECT	GO:0042043~neurexin family protein binding	0.00762415
GOTERM_MF_DIRECT	GO:0017116~single-stranded DNA-dependent ATP-dependent DNA helicase activity	0.00762415
GOTERM_MF_DIRECT	GO:0004672~protein kinase activity	0.008208539
GOTERM_MF_DIRECT	GO:0051015~actin filament binding	0.009431858
GOTERM_MF_DIRECT	GO:0042166~acetylcholine binding	0.012836569
GOTERM_MF_DIRECT	GO:0017147~Wnt-protein binding	0.012846848
GOTERM_MF_DIRECT	GO:0004653~polypeptide N-acetylgalactosaminyltransferase activity	0.012904668
GOTERM_MF_DIRECT	GO:0003779~actin binding	0.013339661
GOTERM_MF_DIRECT	GO:0008201~heparin binding	0.013558292
GOTERM_MF_DIRECT	GO:0051010~microtubule plus-end binding	0.014579601
GOTERM_MF_DIRECT	GO:0019900~kinase binding	0.015478078
GOTERM_MF_DIRECT	GO:0003682~chromatin binding	0.015912974

GOTERM_MF_DIRECT	GO:0038023~signaling receptor activity	0.016569926
GOTERM_MF_DIRECT	GO:0035717~chemokine (C-C motif) ligand 7 binding	0.01709261
GOTERM_MF_DIRECT	GO:0031727~CCR2 chemokine receptor binding	0.01709261
GOTERM_MF_DIRECT	GO:0016810~hydrolase activity, acting on carbon-nitrogen (but not peptide) bonds	0.019366559
GOTERM_MF_DIRECT	GO:0016887~ATPase activity	0.019746322
GOTERM_MF_DIRECT	GO:0005096~GTPase activator activity	0.02010883
GOTERM_MF_DIRECT	GO:0016493~C-C chemokine receptor activity	0.024093783
GOTERM_MF_DIRECT	GO:0016538~cyclin-dependent protein serine/threonine kinase regulator activity	0.025449235
GOTERM_MF_DIRECT	GO:0001786~phosphatidylserine binding	0.026697806
GOTERM_MF_DIRECT	GO:0046982~protein heterodimerization activity	0.026943997
GOTERM_MF_DIRECT	GO:0019904~protein domain specific binding	0.028179428
GOTERM_MF_DIRECT	GO:0005540~hyaluronic acid binding	0.028876843
GOTERM_MF_DIRECT	GO:0042803~protein homodimerization activity	0.030555237
GOTERM_MF_DIRECT	GO:0035173~histone kinase activity	0.032432706
GOTERM_MF_DIRECT	GO:0042802~identical protein binding	0.034531888
GOTERM_MF_DIRECT	GO:0043139~5'-3' DNA helicase activity	0.036928109
GOTERM_MF_DIRECT	GO:0005244~voltage-gated ion channel activity	0.044372098
GOTERM_MF_DIRECT	GO:0005243~gap junction channel activity	0.04582393
GOTERM_MF_DIRECT	GO:0042577~lipid phosphatase activity	0.047917093
KEGG_PATHWAY	ssc04110:Cell cycle	1.22E-18
KEGG_PATHWAY	ssc04814:Motor proteins	6.44E-09
KEGG_PATHWAY	ssc03460:Fanconi anemia pathway	2.41E-06
KEGG_PATHWAY	ssc04360:Axon guidance	3.09E-06
KEGG_PATHWAY	ssc05226:Gastric cancer	8.55E-06
KEGG_PATHWAY	ssc03030:DNA replication	4.11E-05
KEGG_PATHWAY	ssc04914:Progesterone-mediated oocyte maturation	6.30E-05
KEGG_PATHWAY	ssc00100:Steroid biosynthesis	6.74E-05
KEGG_PATHWAY	ssc04218:Cellular senescence	1.19E-04
KEGG_PATHWAY	ssc00514:Other types of O-glycan biosynthesis	1.76E-04
KEGG_PATHWAY	ssc00240:Pyrimidine metabolism	2.21E-04
KEGG_PATHWAY	ssc04114:Oocyte meiosis	3.16E-04
KEGG_PATHWAY	ssc04540:Gap junction	7.03E-04
KEGG_PATHWAY	ssc05100:Bacterial invasion of epithelial cells	7.04E-04
KEGG_PATHWAY	ssc05222:Small cell lung cancer	7.81E-04
KEGG_PATHWAY	ssc04015:Rap1 signaling pathway	9.51E-04
KEGG_PATHWAY	ssc04115:p53 signaling pathway	0.00141376
KEGG_PATHWAY	ssc05224:Breast cancer	0.001687543
KEGG_PATHWAY	ssc04974:Protein digestion and absorption	0.002463506
KEGG_PATHWAY	ssc05225:Hepatocellular carcinoma	0.003495711
KEGG_PATHWAY	ssc01232:Nucleotide metabolism	0.003837496
KEGG_PATHWAY	ssc04390:Hippo signaling pathway	0.003939688
KEGG_PATHWAY	ssc05206:MicroRNAs in cancer	0.004802201

KEGG_PATHWAY	ssc04810:Regulation of actin cytoskeleton	0.005311432
KEGG_PATHWAY	ssc05218:Melanoma	0.006171148
KEGG_PATHWAY	ssc01521:EGFR tyrosine kinase inhibitor resistance	0.006456221
KEGG_PATHWAY	ssc05034:Alcoholism	0.006803893
KEGG_PATHWAY	ssc05214:Glioma	0.007780596
KEGG_PATHWAY	ssc04514:Cell adhesion molecules	0.008168595
KEGG_PATHWAY	ssc00983:Drug metabolism - other enzymes	0.011749521
KEGG_PATHWAY	ssc04012:ErbB signaling pathway	0.011968686
KEGG_PATHWAY	ssc05203:Viral carcinogenesis	0.013006502
KEGG_PATHWAY	ssc04014:Ras signaling pathway	0.015098466
KEGG_PATHWAY	ssc01100:Metabolic pathways	0.018278621
KEGG_PATHWAY	ssc05200:Pathways in cancer	0.018513264
KEGG_PATHWAY	ssc05207:Chemical carcinogenesis - receptor activation	0.022915201
KEGG_PATHWAY	ssc04310:Wnt signaling pathway	0.023097658
KEGG_PATHWAY	ssc00900:Terpenoid backbone biosynthesis	0.02385517
KEGG_PATHWAY	ssc05166:Human T-cell leukemia virus 1 infection	0.025272247
KEGG_PATHWAY	ssc00250:Alanine, aspartate and glutamate metabolism	0.028141953
KEGG_PATHWAY	ssc04510:Focal adhesion	0.035560791
KEGG_PATHWAY	ssc04020:Calcium signaling pathway	0.038975046
KEGG_PATHWAY	ssc05165:Human papillomavirus infection	0.048612776
KEGG_PATHWAY	ssc04151:PI3K-Akt signaling pathway	0.048656506
KEGG_PATHWAY	ssc04010:MAPK signaling pathway	0.04948653
KEGG_PATHWAY	ssc01230:Biosynthesis of amino acids	0.049530672
KEGG_PATHWAY	ssc00512:Mucin type O-glycan biosynthesis	0.049659778

E70＞E45

Category	Term	PValue
GOTERM_BP_DIRECT	GO:0006936~muscle contraction	7.29E-08
GOTERM_BP_DIRECT	GO:0019882~antigen processing and presentation	9.66E-06
GOTERM_BP_DIRECT	GO:0045214~sarcomere organization	3.97E-05
GOTERM_BP_DIRECT	GO:0003009~skeletal muscle contraction	4.30E-05
GOTERM_BP_DIRECT	GO:0002504~antigen processing and presentation of peptide or polysaccharide antigen via MHC class II	4.30E-05
GOTERM_BP_DIRECT	GO:0018120~peptidyl-arginine ADP-ribosylation	2.81E-04
GOTERM_BP_DIRECT	GO:0035725~sodium ion transmembrane transport	5.17E-04
GOTERM_BP_DIRECT	GO:0030500~regulation of bone mineralization	6.79E-04
GOTERM_BP_DIRECT	GO:0007605~sensory perception of sound	8.40E-04
GOTERM_BP_DIRECT	GO:0019227~neuronal action potential propagation	0.001316772
GOTERM_BP_DIRECT	GO:0002250~adaptive immune response	0.002347422
GOTERM_BP_DIRECT	GO:0034765~regulation of ion transmembrane transport	0.002988114
GOTERM_BP_DIRECT	GO:0002503~peptide antigen assembly with MHC class II protein complex	0.005033537
GOTERM_BP_DIRECT	GO:0035995~detection of muscle stretch	0.00507394

GOTERM_BP_DIRECT	GO:0071356~cellular response to tumor necrosis factor	0.005456401
GOTERM_BP_DIRECT	GO:0071277~cellular response to calcium ion	0.006202179
GOTERM_BP_DIRECT	GO:0046330~positive regulation of JNK cascade	0.007168773
GOTERM_BP_DIRECT	GO:0048741~skeletal muscle fiber development	0.008540684
GOTERM_BP_DIRECT	GO:0051480~regulation of cytosolic calcium ion concentration	0.008605742
GOTERM_BP_DIRECT	GO:0002063~chondrocyte development	0.00928905
GOTERM_BP_DIRECT	GO:0006508~proteolysis	0.009468911
GOTERM_BP_DIRECT	GO:0035914~skeletal muscle cell differentiation	0.012723135
GOTERM_BP_DIRECT	GO:0019886~antigen processing and presentation of exogenous peptide antigen via MHC class II	0.015131065
GOTERM_BP_DIRECT	GO:0007015~actin filament organization	0.015488028
GOTERM_BP_DIRECT	GO:0097647~amylin receptor signaling pathway	0.01599264
GOTERM_BP_DIRECT	GO:0019233~sensory perception of pain	0.018922593
GOTERM_BP_DIRECT	GO:0006096~glycolytic process	0.021644135
GOTERM_BP_DIRECT	GO:0019933~cAMP-mediated signaling	0.022628487
GOTERM_BP_DIRECT	GO:0086073~bundle of His cell-Purkinje myocyte adhesion involved in cell communication	0.023330574
GOTERM_BP_DIRECT	GO:0001768~establishment of T cell polarity	0.023330574
GOTERM_BP_DIRECT	GO:0090279~regulation of calcium ion import	0.023330574
GOTERM_BP_DIRECT	GO:0045766~positive regulation of angiogenesis	0.023781679
GOTERM_BP_DIRECT	GO:0042060~wound healing	0.02454983
GOTERM_BP_DIRECT	GO:0007165~signal transduction	0.025368651
GOTERM_BP_DIRECT	GO:0086091~regulation of heart rate by cardiac conduction	0.027772363
GOTERM_BP_DIRECT	GO:0006955~immune response	0.028456043
GOTERM_BP_DIRECT	GO:0033210~leptin-mediated signaling pathway	0.031769601
GOTERM_BP_DIRECT	GO:0030239~myofibril assembly	0.031769601
GOTERM_BP_DIRECT	GO:0005980~glycogen catabolic process	0.031769601
GOTERM_BP_DIRECT	GO:2000378~negative regulation of reactive oxygen species metabolic process	0.031799474
GOTERM_BP_DIRECT	GO:0010595~positive regulation of endothelial cell migration	0.034834198
GOTERM_BP_DIRECT	GO:0050870~positive regulation of T cell activation	0.037006883
GOTERM_BP_DIRECT	GO:0030073~insulin secretion	0.037006883
GOTERM_BP_DIRECT	GO:0007519~skeletal muscle tissue development	0.038719895
GOTERM_BP_DIRECT	GO:0051897~positive regulation of protein kinase B signaling	0.038991082
GOTERM_BP_DIRECT	GO:0007156~homophilic cell adhesion via plasma membrane adhesion molecules	0.040462343
GOTERM_BP_DIRECT	GO:0014808~release of sequestered calcium ion into cytosol by sarcoplasmic reticulum	0.041205325
GOTERM_BP_DIRECT	GO:0060347~heart trabecula formation	0.041205325
GOTERM_BP_DIRECT	GO:0090331~negative regulation of platelet aggregation	0.041205325

GOTERM_BP_DIRECT	GO:0051694~pointed-end actin filament capping	0.041205325
GOTERM_BP_DIRECT	GO:0008637~apoptotic mitochondrial changes	0.041205325
GOTERM_BP_DIRECT	GO:0007399~nervous system development	0.042479719
GOTERM_BP_DIRECT	GO:0007189~adenylate cyclase-activating G-protein coupled receptor signaling pathway	0.042479719
GOTERM_BP_DIRECT	GO:0046716~muscle cell cellular homeostasis	0.042621591
GOTERM_BP_DIRECT	GO:0006836~neurotransmitter transport	0.042621591
GOTERM_BP_DIRECT	GO:0019228~neuronal action potential	0.048635868
GOTERM_BP_DIRECT	GO:0042593~glucose homeostasis	0.04926494
GOTERM_CC_DIRECT	GO:0030018~Z disc	8.63E-10
GOTERM_CC_DIRECT	GO:0042383~sarcolemma	5.40E-07
GOTERM_CC_DIRECT	GO:0030016~myofibril	7.27E-07
GOTERM_CC_DIRECT	GO:0005615~extracellular space	5.69E-06
GOTERM_CC_DIRECT	GO:0042613~MHC class II protein complex	7.20E-06
GOTERM_CC_DIRECT	GO:0016459~myosin complex	7.28E-06
GOTERM_CC_DIRECT	GO:0030315~T-tubule	6.55E-05
GOTERM_CC_DIRECT	GO:0033017~sarcoplasmic reticulum membrane	1.57E-04
GOTERM_CC_DIRECT	GO:0005887~integral component of plasma membrane	2.07E-04
GOTERM_CC_DIRECT	GO:0032982~myosin filament	4.90E-04
GOTERM_CC_DIRECT	GO:0005576~extracellular region	8.40E-04
GOTERM_CC_DIRECT	GO:0016323~basolateral plasma membrane	0.001002673
GOTERM_CC_DIRECT	GO:0030424~axon	0.00133745
GOTERM_CC_DIRECT	GO:0014704~intercalated disc	0.002446989
GOTERM_CC_DIRECT	GO:0043209~myelin sheath	0.00387684
GOTERM_CC_DIRECT	GO:0001725~stress fiber	0.003904883
GOTERM_CC_DIRECT	GO:0005856~cytoskeleton	0.004407654
GOTERM_CC_DIRECT	GO:0005737~cytoplasm	0.006149804
GOTERM_CC_DIRECT	GO:0015629~actin cytoskeleton	0.008200562
GOTERM_CC_DIRECT	GO:0016324~apical plasma membrane	0.009352333
GOTERM_CC_DIRECT	GO:0005865~striated muscle thin filament	0.018887145
GOTERM_CC_DIRECT	GO:0031012~extracellular matrix	0.023877178
GOTERM_CC_DIRECT	GO:0000786~nucleosome	0.02706078
GOTERM_CC_DIRECT	GO:0001518~voltage-gated sodium channel complex	0.032141693
GOTERM_CC_DIRECT	GO:0016529~sarcoplasmic reticulum	0.032141693
GOTERM_CC_DIRECT	GO:0033268~node of Ranvier	0.033552296
GOTERM_CC_DIRECT	GO:0043235~receptor complex	0.04363192
GOTERM_MF_DIRECT	GO:0051015~actin filament binding	1.79E-07
GOTERM_MF_DIRECT	GO:0005509~calcium ion binding	3.91E-06
GOTERM_MF_DIRECT	GO:0003779~actin binding	6.87E-06
GOTERM_MF_DIRECT	GO:0008307~structural constituent of muscle	1.13E-05
GOTERM_MF_DIRECT	GO:0003774~motor activity	4.22E-05
GOTERM_MF_DIRECT	GO:0044325~ion channel binding	0.001213279
GOTERM_MF_DIRECT	GO:0005244~voltage-gated ion channel activity	0.001634537
GOTERM_MF_DIRECT	GO:0004556~alpha-amylase activity	0.001729824

GOTERM_MF_DIRECT	GO:0031432~titin binding	0.002689416
GOTERM_MF_DIRECT	GO:0000146~microfilament motor activity	0.005168386
GOTERM_MF_DIRECT	GO:0008270~zinc ion binding	0.005380313
GOTERM_MF_DIRECT	GO:0023026~MHC class II protein complex binding	0.005442498
GOTERM_MF_DIRECT	GO:0005516~calmodulin binding	0.00639509
GOTERM_MF_DIRECT	GO:0005332~gamma-aminobutyric acid:sodium symporter activity	0.00830311
GOTERM_MF_DIRECT	GO:0004029~aldehyde dehydrogenase (NAD) activity	0.009425141
GOTERM_MF_DIRECT	GO:0031433~telethonin binding	0.0134895
GOTERM_MF_DIRECT	GO:0070006~metalloaminopeptidase activity	0.016149427
GOTERM_MF_DIRECT	GO:0022848~acetylcholine-gated cation-selective channel activity	0.021421911
GOTERM_MF_DIRECT	GO:0005523~tropomyosin binding	0.021421911
GOTERM_MF_DIRECT	GO:0005518~collagen binding	0.025448917
GOTERM_MF_DIRECT	GO:0046982~protein heterodimerization activity	0.037493425
GOTERM_MF_DIRECT	GO:0005248~voltage-gated sodium channel activity	0.038976281
GOTERM_MF_DIRECT	GO:0005245~voltage-gated calcium channel activity	0.039488655
GOTERM_MF_DIRECT	GO:0017080~sodium channel regulator activity	0.044220172
GOTERM_MF_DIRECT	GO:0015459~potassium channel regulator activity	0.044220172
GOTERM_MF_DIRECT	GO:0004879~RNA polymerase II transcription factor activity, ligand-activated sequence-specific DNA binding	0.047648898
GOTERM_MF_DIRECT	GO:0000976~transcription regulatory region sequence-specific DNA binding	0.048532619
KEGG_PATHWAY	ssc04020:Calcium signaling pathway	2.00E-08
KEGG_PATHWAY	ssc04814:Motor proteins	2.09E-08
KEGG_PATHWAY	ssc05412:Arrhythmogenic right ventricular cardiomyopathy	6.24E-07
KEGG_PATHWAY	ssc05320:Autoimmune thyroid disease	1.33E-06
KEGG_PATHWAY	ssc05321:Inflammatory bowel disease	5.44E-06
KEGG_PATHWAY	ssc05322:Systemic lupus erythematosus	3.24E-05
KEGG_PATHWAY	ssc05332:Graft-versus-host disease	7.52E-05
KEGG_PATHWAY	ssc05310:Asthma	8.95E-05
KEGG_PATHWAY	ssc05330:Allograft rejection	1.31E-04
KEGG_PATHWAY	ssc05414:Dilated cardiomyopathy	1.95E-04
KEGG_PATHWAY	ssc00500:Starch and sucrose metabolism	2.08E-04
KEGG_PATHWAY	ssc04940:Type I diabetes mellitus	3.44E-04
KEGG_PATHWAY	ssc04640:Hematopoietic cell lineage	4.33E-04
KEGG_PATHWAY	ssc04970:Salivary secretion	4.77E-04
KEGG_PATHWAY	ssc05410:Hypertrophic cardiomyopathy	4.77E-04
KEGG_PATHWAY	ssc04612:Antigen processing and presentation	4.89E-04
KEGG_PATHWAY	ssc04024:cAMP signaling pathway	7.11E-04
KEGG_PATHWAY	ssc04659:Th17 cell differentiation	8.25E-04
KEGG_PATHWAY	ssc04972:Pancreatic secretion	0.00107425
KEGG_PATHWAY	ssc04060:Cytokine-cytokine receptor interaction	0.001262186

KEGG_PATHWAY	ssc04710:Circadian rhythm	0.001642638
KEGG_PATHWAY	ssc04512:ECM-receptor interaction	0.001794037
KEGG_PATHWAY	ssc00010:Glycolysis / Gluconeogenesis	0.001965284
KEGG_PATHWAY	ssc05323:Rheumatoid arthritis	0.002297086
KEGG_PATHWAY	ssc04672:Intestinal immune network for IgA production	0.002321157
KEGG_PATHWAY	ssc05164:Influenza A	0.002965327
KEGG_PATHWAY	ssc04261:Adrenergic signaling in cardiomyocytes	0.00360864
KEGG_PATHWAY	ssc04514:Cell adhesion molecules	0.005049599
KEGG_PATHWAY	ssc05140:Leishmaniasis	0.005492711
KEGG_PATHWAY	ssc04658:Th1 and Th2 cell differentiation	0.007518088
KEGG_PATHWAY	ssc05416:Viral myocarditis	0.010926067
KEGG_PATHWAY	ssc04260:Cardiac muscle contraction	0.015292767
KEGG_PATHWAY	ssc04920:Adipocytokine signaling pathway	0.017127289
KEGG_PATHWAY	ssc04080:Neuroactive ligand-receptor interaction	0.018738011
KEGG_PATHWAY	ssc04145:Phagosome	0.019126248
KEGG_PATHWAY	ssc05150:Staphylococcus aureus infection	0.020794811
KEGG_PATHWAY	ssc04810:Regulation of actin cytoskeleton	0.022863597
KEGG_PATHWAY	ssc05169:Epstein-Barr virus infection	0.023935033
KEGG_PATHWAY	ssc04930:Type II diabetes mellitus	0.029450577
KEGG_PATHWAY	ssc04390:Hippo signaling pathway	0.029555654
KEGG_PATHWAY	ssc00051:Fructose and mannose metabolism	0.029675964
KEGG_PATHWAY	ssc04217:Necroptosis	0.032052717
KEGG_PATHWAY	ssc00410:beta-Alanine metabolism	0.032812437
KEGG_PATHWAY	ssc04510:Focal adhesion	0.035588939
KEGG_PATHWAY	ssc05165:Human papillomavirus infection	0.036755922
KEGG_PATHWAY	ssc04152:AMPK signaling pathway	0.037717119
KEGG_PATHWAY	ssc04911:Insulin secretion	0.038303956
KEGG_PATHWAY	ssc00340:Histidine metabolism	0.046449137
KEGG_PATHWAY	ssc00350:Tyrosine metabolism	0.0472283

E70＞E100

Category	Term	PValue
GOTERM_BP_DIRECT	GO:0007018~microtubule-based movement	7.70E-10
GOTERM_BP_DIRECT	GO:0007409~axonogenesis	5.37E-06
GOTERM_BP_DIRECT	GO:0007094~mitotic spindle assembly checkpoint	4.43E-05
GOTERM_BP_DIRECT	GO:0000278~mitotic cell cycle	4.49E-05
GOTERM_BP_DIRECT	GO:0051965~positive regulation of synapse assembly	4.73E-05
GOTERM_BP_DIRECT	GO:0007156~homophilic cell adhesion via plasma membrane adhesion molecules	9.72E-05
GOTERM_BP_DIRECT	GO:0007399~nervous system development	1.08E-04
GOTERM_BP_DIRECT	GO:0051301~cell division	1.40E-04
GOTERM_BP_DIRECT	GO:0007411~axon guidance	2.73E-04
GOTERM_BP_DIRECT	GO:0007080~mitotic metaphase plate congression	3.33E-04

GOTERM_BP_DIRECT	GO:0007157~heterophilic cell-cell adhesion via plasma membrane cell adhesion molecules	3.33E-04
GOTERM_BP_DIRECT	GO:0000281~mitotic cytokinesis	3.69E-04
GOTERM_BP_DIRECT	GO:1905606~regulation of presynapse assembly	4.09E-04
GOTERM_BP_DIRECT	GO:0009887~animal organ morphogenesis	9.70E-04
GOTERM_BP_DIRECT	GO:0007019~microtubule depolymerization	9.79E-04
GOTERM_BP_DIRECT	GO:0006270~DNA replication initiation	0.001095
GOTERM_BP_DIRECT	GO:0034765~regulation of ion transmembrane transport	0.001265
GOTERM_BP_DIRECT	GO:0006695~cholesterol biosynthetic process	0.001405
GOTERM_BP_DIRECT	GO:0007059~chromosome segregation	0.001685
GOTERM_BP_DIRECT	GO:0007420~brain development	0.001982
GOTERM_BP_DIRECT	GO:0051382~kinetochore assembly	0.002131
GOTERM_BP_DIRECT	GO:0086010~membrane depolarization during action potential	0.002958
GOTERM_BP_DIRECT	GO:0030334~regulation of cell migration	0.003701
GOTERM_BP_DIRECT	GO:0000070~mitotic sister chromatid segregation	0.003796
GOTERM_BP_DIRECT	GO:0090307~mitotic spindle assembly	0.005039
GOTERM_BP_DIRECT	GO:0098742~cell-cell adhesion via plasma-membrane adhesion molecules	0.005646
GOTERM_BP_DIRECT	GO:0000086~G2/M transition of mitotic cell cycle	0.005868
GOTERM_BP_DIRECT	GO:0035725~sodium ion transmembrane transport	0.006792
GOTERM_BP_DIRECT	GO:0016126~sterol biosynthetic process	0.008075
GOTERM_BP_DIRECT	GO:0099560~synaptic membrane adhesion	0.008412
GOTERM_BP_DIRECT	GO:0043524~negative regulation of neuron apoptotic process	0.010543
GOTERM_BP_DIRECT	GO:0010976~positive regulation of neuron projection development	0.010974
GOTERM_BP_DIRECT	GO:0008284~positive regulation of cell proliferation	0.011588
GOTERM_BP_DIRECT	GO:0090090~negative regulation of canonical Wnt signaling pathway	0.011905
GOTERM_BP_DIRECT	GO:0001501~skeletal system development	0.012193
GOTERM_BP_DIRECT	GO:0007155~cell adhesion	0.01465
GOTERM_BP_DIRECT	GO:0030199~collagen fibril organization	0.014661
GOTERM_BP_DIRECT	GO:0007051~spindle organization	0.01473
GOTERM_BP_DIRECT	GO:0060174~limb bud formation	0.01473
GOTERM_BP_DIRECT	GO:0008203~cholesterol metabolic process	0.014919
GOTERM_BP_DIRECT	GO:0019228~neuronal action potential	0.015228
GOTERM_BP_DIRECT	GO:0048514~blood vessel morphogenesis	0.01893
GOTERM_BP_DIRECT	GO:0048566~embryonic digestive tract development	0.01893
GOTERM_BP_DIRECT	GO:0030916~otic vesicle formation	0.022083
GOTERM_BP_DIRECT	GO:0010389~regulation of G2/M transition of mitotic cell cycle	0.022083
GOTERM_BP_DIRECT	GO:0070384~Harderian gland development	0.022083
GOTERM_BP_DIRECT	GO:0021707~cerebellar granule cell differentiation	0.022083

GOTERM_BP_DIRECT	GO:0046602~regulation of mitotic centrosome separation	0.022083
GOTERM_BP_DIRECT	GO:0034501~protein localization to kinetochore	0.023721
GOTERM_BP_DIRECT	GO:0007043~cell-cell junction assembly	0.024766
GOTERM_BP_DIRECT	GO:0008543~fibroblast growth factor receptor signaling pathway	0.027634
GOTERM_BP_DIRECT	GO:0001578~microtubule bundle formation	0.02859
GOTERM_BP_DIRECT	GO:0032331~negative regulation of chondrocyte differentiation	0.029105
GOTERM_BP_DIRECT	GO:0032482~Rab protein signal transduction	0.029105
GOTERM_BP_DIRECT	GO:0032466~negative regulation of cytokinesis	0.032049
GOTERM_BP_DIRECT	GO:0007052~mitotic spindle organization	0.034249
GOTERM_BP_DIRECT	GO:0007064~mitotic sister chromatid cohesion	0.035076
GOTERM_BP_DIRECT	GO:0031032~actomyosin structure organization	0.037234
GOTERM_BP_DIRECT	GO:0060041~retina development in camera-type eye	0.037893
GOTERM_BP_DIRECT	GO:0090263~positive regulation of canonical Wnt signaling pathway	0.038381
GOTERM_BP_DIRECT	GO:0070509~calcium ion import	0.041627
GOTERM_BP_DIRECT	GO:0014032~neural crest cell development	0.041627
GOTERM_BP_DIRECT	GO:0006268~DNA unwinding involved in DNA replication	0.041627
GOTERM_BP_DIRECT	GO:0007017~microtubule-based process	0.042059
GOTERM_BP_DIRECT	GO:0007416~synapse assembly	0.042059
GOTERM_BP_DIRECT	GO:0051983~regulation of chromosome segregation	0.04342
GOTERM_BP_DIRECT	GO:0018243~protein O-linked glycosylation via threonine	0.04342
GOTERM_BP_DIRECT	GO:0060371~regulation of atrial cardiac muscle cell membrane depolarization	0.04342
GOTERM_BP_DIRECT	GO:0033314~mitotic DNA replication checkpoint	0.04342
GOTERM_BP_DIRECT	GO:2000096~positive regulation of Wnt signaling pathway, planar cell polarity pathway	0.04342
GOTERM_BP_DIRECT	GO:0042416~dopamine biosynthetic process	0.04342
GOTERM_BP_DIRECT	GO:0007049~cell cycle	0.045285
GOTERM_BP_DIRECT	GO:0014068~positive regulation of phosphatidylinositol 3-kinase signaling	0.045697
GOTERM_BP_DIRECT	GO:0051781~positive regulation of cell division	0.045869
GOTERM_BP_DIRECT	GO:0042472~inner ear morphogenesis	0.045869
GOTERM_CC_DIRECT	GO:0098978~glutamatergic synapse	1.30E-08
GOTERM_CC_DIRECT	GO:0005874~microtubule	1.25E-07
GOTERM_CC_DIRECT	GO:0001518~voltage-gated sodium channel complex	9.18E-07
GOTERM_CC_DIRECT	GO:0005576~extracellular region	2.61E-06
GOTERM_CC_DIRECT	GO:0000776~kinetochore	3.47E-06
GOTERM_CC_DIRECT	GO:0031012~extracellular matrix	9.17E-06
GOTERM_CC_DIRECT	GO:0005871~kinesin complex	2.08E-05
GOTERM_CC_DIRECT	GO:0005737~cytoplasm	3.63E-05
GOTERM_CC_DIRECT	GO:0045171~intercellular bridge	4.75E-05
GOTERM_CC_DIRECT	GO:0043005~neuron projection	5.46E-05

GOTERM_CC_DIRECT	GO:0045202~synapse	2.32E-04
GOTERM_CC_DIRECT	GO:0030424~axon	2.72E-04
GOTERM_CC_DIRECT	GO:0000922~spindle pole	3.09E-04
GOTERM_CC_DIRECT	GO:0072686~mitotic spindle	5.30E-04
GOTERM_CC_DIRECT	GO:0098685~Schaffer collateral - CA1 synapse	0.001847
GOTERM_CC_DIRECT	GO:0005887~integral component of plasma membrane	0.001932
GOTERM_CC_DIRECT	GO:0000940~condensed chromosome outer kinetochore	0.002261
GOTERM_CC_DIRECT	GO:0043025~neuronal cell body	0.003985
GOTERM_CC_DIRECT	GO:0099061~integral component of postsynaptic density membrane	0.004133
GOTERM_CC_DIRECT	GO:0009986~cell surface	0.00495
GOTERM_CC_DIRECT	GO:0005819~spindle	0.006058
GOTERM_CC_DIRECT	GO:0043197~dendritic spine	0.006515
GOTERM_CC_DIRECT	GO:0099056~integral component of presynaptic membrane	0.008059
GOTERM_CC_DIRECT	GO:0098982~GABA-ergic synapse	0.009291
GOTERM_CC_DIRECT	GO:0005912~adherens junction	0.010212
GOTERM_CC_DIRECT	GO:0005588~collagen type V trimer	0.011874
GOTERM_CC_DIRECT	GO:0030425~dendrite	0.011938
GOTERM_CC_DIRECT	GO:0005892~acetylcholine-gated channel complex	0.012062
GOTERM_CC_DIRECT	GO:0005615~extracellular space	0.012958
GOTERM_CC_DIRECT	GO:0016342~catenin complex	0.016663
GOTERM_CC_DIRECT	GO:0014069~postsynaptic density	0.019884
GOTERM_CC_DIRECT	GO:0030496~midbody	0.023401
GOTERM_CC_DIRECT	GO:0043296~apical junction complex	0.029021
GOTERM_CC_DIRECT	GO:0031594~neuromuscular junction	0.035109
GOTERM_CC_DIRECT	GO:0005813~centrosome	0.038037
GOTERM_CC_DIRECT	GO:0032580~Golgi cisterna membrane	0.04215
GOTERM_CC_DIRECT	GO:0002177~manchette	0.049033
GOTERM_MF_DIRECT	GO:0003777~microtubule motor activity	1.09E-12
GOTERM_MF_DIRECT	GO:0008017~microtubule binding	6.41E-11
GOTERM_MF_DIRECT	GO:0008574~ATP-dependent microtubule motor activity, plus-end-directed	6.29E-06
GOTERM_MF_DIRECT	GO:0005248~voltage-gated sodium channel activity	1.79E-05
GOTERM_MF_DIRECT	GO:0005509~calcium ion binding	1.95E-05
GOTERM_MF_DIRECT	GO:0005201~extracellular matrix structural constituent	1.88E-04
GOTERM_MF_DIRECT	GO:0005540~hyaluronic acid binding	4.47E-04
GOTERM_MF_DIRECT	GO:0005524~ATP binding	5.93E-04
GOTERM_MF_DIRECT	GO:0005244~voltage-gated ion channel activity	7.13E-04
GOTERM_MF_DIRECT	GO:0001540~beta-amyloid binding	0.001313
GOTERM_MF_DIRECT	GO:0050839~cell adhesion molecule binding	0.001375
GOTERM_MF_DIRECT	GO:0051015~actin filament binding	0.002061
GOTERM_MF_DIRECT	GO:0005104~fibroblast growth factor receptor binding	0.003245
GOTERM_MF_DIRECT	GO:0003779~actin binding	0.003703
GOTERM_MF_DIRECT	GO:0008201~heparin binding	0.005238

GOTERM_MF_DIRECT	GO:0016887~ATPase activity	0.006581
GOTERM_MF_DIRECT	GO:0005102~receptor binding	0.008119
GOTERM_MF_DIRECT	GO:0008013~beta-catenin binding	0.008949
GOTERM_MF_DIRECT	GO:0005178~integrin binding	0.010218
GOTERM_MF_DIRECT	GO:0030506~ankyrin binding	0.011718
GOTERM_MF_DIRECT	GO:0005506~iron ion binding	0.012964
GOTERM_MF_DIRECT	GO:0004029~aldehyde dehydrogenase (NAD) activity	0.015104
GOTERM_MF_DIRECT	GO:0045296~cadherin binding	0.015498
GOTERM_MF_DIRECT	GO:0030246~carbohydrate binding	0.016153
GOTERM_MF_DIRECT	GO:0008046~axon guidance receptor activity	0.018807
GOTERM_MF_DIRECT	GO:0043515~kinetochore binding	0.018807
GOTERM_MF_DIRECT	GO:0005200~structural constituent of cytoskeleton	0.022864
GOTERM_MF_DIRECT	GO:0008376~acetylgalactosaminyltransferase activity	0.023359
GOTERM_MF_DIRECT	GO:0016620~oxidoreductase activity, acting on the aldehyde or oxo group of donors, NAD or NADP as acceptor	0.028234
GOTERM_MF_DIRECT	GO:0008083~growth factor activity	0.032976
GOTERM_MF_DIRECT	GO:0022848~acetylcholine-gated cation-selective channel activity	0.033604
GOTERM_MF_DIRECT	GO:0045295~gamma-catenin binding	0.037177
GOTERM_MF_DIRECT	GO:0005272~sodium channel activity	0.037177
GOTERM_MF_DIRECT	GO:0042166~acetylcholine binding	0.037177
GOTERM_MF_DIRECT	GO:0015293~symporter activity	0.048126
GOTERM_MF_DIRECT	GO:0004672~protein kinase activity	0.049694
KEGG_PATHWAY	ssc04814:Motor proteins	1.94E-12
KEGG_PATHWAY	ssc04110:Cell cycle	1.38E-07
KEGG_PATHWAY	ssc04514:Cell adhesion molecules	1.05E-04
KEGG_PATHWAY	ssc00100:Steroid biosynthesis	1.36E-04
KEGG_PATHWAY	ssc04974:Protein digestion and absorption	4.64E-04
KEGG_PATHWAY	ssc05226:Gastric cancer	0.001732
KEGG_PATHWAY	ssc03030:DNA replication	0.003321
KEGG_PATHWAY	ssc04512:ECM-receptor interaction	0.004905
KEGG_PATHWAY	ssc04020:Calcium signaling pathway	0.006664
KEGG_PATHWAY	ssc00900:Terpenoid backbone biosynthesis	0.01304
KEGG_PATHWAY	ssc04080:Neuroactive ligand-receptor interaction	0.014551
KEGG_PATHWAY	ssc05224:Breast cancer	0.021284
KEGG_PATHWAY	ssc05218:Melanoma	0.027352
KEGG_PATHWAY	ssc05144:Malaria	0.029101
KEGG_PATHWAY	ssc01100:Metabolic pathways	0.032171
KEGG_PATHWAY	ssc04390:Hippo signaling pathway	0.033538
KEGG_PATHWAY	ssc04360:Axon guidance	0.048074
KEGG_PATHWAY	ssc04930:Type II diabetes mellitus	0.049043
KEGG_PATHWAY	ssc00514:Other types of O-glycan biosynthesis	0.049043

E100＞E45

Category	Term	PValue
GOTERM_BP_DIRECT	GO:0032981~mitochondrial respiratory chain complex I assembly	3.00E-08
GOTERM_BP_DIRECT	GO:0006099~tricarboxylic acid cycle	2.30E-07
GOTERM_BP_DIRECT	GO:0006936~muscle contraction	1.54E-06
GOTERM_BP_DIRECT	GO:0045214~sarcomere organization	5.90E-06
GOTERM_BP_DIRECT	GO:0022904~respiratory electron transport chain	5.11E-05
GOTERM_BP_DIRECT	GO:0032496~response to lipopolysaccharide	1.22E-04
GOTERM_BP_DIRECT	GO:0033539~fatty acid beta-oxidation using acyl-CoA dehydrogenase	1.32E-04
GOTERM_BP_DIRECT	GO:0045766~positive regulation of angiogenesis	1.41E-04
GOTERM_BP_DIRECT	GO:0006122~mitochondrial electron transport, ubiquinol to cytochrome c	2.75E-04
GOTERM_BP_DIRECT	GO:0019882~antigen processing and presentation	3.57E-04
GOTERM_BP_DIRECT	GO:0003009~skeletal muscle contraction	6.97E-04
GOTERM_BP_DIRECT	GO:0002504~antigen processing and presentation of peptide or polysaccharide antigen via MHC class II	6.97E-04
GOTERM_BP_DIRECT	GO:0006123~mitochondrial electron transport, cytochrome c to oxygen	0.001082223
GOTERM_BP_DIRECT	GO:0014898~cardiac muscle hypertrophy in response to stress	0.001500604
GOTERM_BP_DIRECT	GO:0006120~mitochondrial electron transport, NADH to ubiquinone	0.001515102
GOTERM_BP_DIRECT	GO:0048741~skeletal muscle fiber development	0.002135184
GOTERM_BP_DIRECT	GO:0001525~angiogenesis	0.002372504
GOTERM_BP_DIRECT	GO:0055013~cardiac muscle cell development	0.002529474
GOTERM_BP_DIRECT	GO:0001516~prostaglandin biosynthetic process	0.00299332
GOTERM_BP_DIRECT	GO:0043552~positive regulation of phosphatidylinositol 3-kinase activity	0.00299332
GOTERM_BP_DIRECT	GO:0018120~peptidyl-arginine ADP-ribosylation	0.003507211
GOTERM_BP_DIRECT	GO:0034765~regulation of ion transmembrane transport	0.003659184
GOTERM_BP_DIRECT	GO:0001570~vasculogenesis	0.004168513
GOTERM_BP_DIRECT	GO:0086091~regulation of heart rate by cardiac conduction	0.004400277
GOTERM_BP_DIRECT	GO:0006006~glucose metabolic process	0.004404766
GOTERM_BP_DIRECT	GO:0006635~fatty acid beta-oxidation	0.004404766
GOTERM_BP_DIRECT	GO:0006809~nitric oxide biosynthetic process	0.004694744
GOTERM_BP_DIRECT	GO:0045591~positive regulation of regulatory T cell differentiation	0.004694744
GOTERM_BP_DIRECT	GO:0008637~apoptotic mitochondrial changes	0.004694744
GOTERM_BP_DIRECT	GO:0007517~muscle organ development	0.005195339

GOTERM_BP_DIRECT	GO:0045746~negative regulation of Notch signaling pathway	0.00672314
GOTERM_BP_DIRECT	GO:0071456~cellular response to hypoxia	0.007603678
GOTERM_BP_DIRECT	GO:0002503~peptide antigen assembly with MHC class II protein complex	0.00779755
GOTERM_BP_DIRECT	GO:0010628~positive regulation of gene expression	0.007810703
GOTERM_BP_DIRECT	GO:0046716~muscle cell cellular homeostasis	0.007845156
GOTERM_BP_DIRECT	GO:0097647~amylin receptor signaling pathway	0.008126583
GOTERM_BP_DIRECT	GO:0005978~glycogen biosynthetic process	0.008532083
GOTERM_BP_DIRECT	GO:0072593~reactive oxygen species metabolic process	0.008560732
GOTERM_BP_DIRECT	GO:0032728~positive regulation of interferon-beta production	0.008570859
GOTERM_BP_DIRECT	GO:0007519~skeletal muscle tissue development	0.008570859
GOTERM_BP_DIRECT	GO:0006955~immune response	0.010566417
GOTERM_BP_DIRECT	GO:0006633~fatty acid biosynthetic process	0.011675565
GOTERM_BP_DIRECT	GO:0019885~antigen processing and presentation of endogenous peptide antigen via MHC class I	0.011995515
GOTERM_BP_DIRECT	GO:0006103~2-oxoglutarate metabolic process	0.011995515
GOTERM_BP_DIRECT	GO:0061061~muscle structure development	0.011995515
GOTERM_BP_DIRECT	GO:0014850~response to muscle activity	0.011995515
GOTERM_BP_DIRECT	GO:0035458~cellular response to interferon-beta	0.013235485
GOTERM_BP_DIRECT	GO:0019915~lipid storage	0.013299571
GOTERM_BP_DIRECT	GO:0045071~negative regulation of viral genome replication	0.013299571
GOTERM_BP_DIRECT	GO:0006198~cAMP catabolic process	0.015070498
GOTERM_BP_DIRECT	GO:0035914~skeletal muscle cell differentiation	0.015158973
GOTERM_BP_DIRECT	GO:0007173~epidermal growth factor receptor signaling pathway	0.016719971
GOTERM_BP_DIRECT	GO:0050873~brown fat cell differentiation	0.016719971
GOTERM_BP_DIRECT	GO:0034605~cellular response to heat	0.016719971
GOTERM_BP_DIRECT	GO:0007219~Notch signaling pathway	0.016891905
GOTERM_BP_DIRECT	GO:0010460~positive regulation of heart rate	0.017404681
GOTERM_BP_DIRECT	GO:0071260~cellular response to mechanical stimulus	0.018004733
GOTERM_BP_DIRECT	GO:0043484~regulation of RNA splicing	0.018834549
GOTERM_BP_DIRECT	GO:0003180~aortic valve morphogenesis	0.020574732
GOTERM_BP_DIRECT	GO:2000378~negative regulation of reactive oxygen species metabolic process	0.020574732
GOTERM_BP_DIRECT	GO:0034620~cellular response to unfolded protein	0.020574732
GOTERM_BP_DIRECT	GO:0071805~potassium ion transmembrane transport	0.020621747
GOTERM_BP_DIRECT	GO:2001244~positive regulation of intrinsic apoptotic signaling pathway	0.020787488
GOTERM_BP_DIRECT	GO:0006874~cellular calcium ion homeostasis	0.023105382
GOTERM_BP_DIRECT	GO:0010595~positive regulation of endothelial cell migration	0.0235451

GOTERM_BP_DIRECT	GO:0055119~relaxation of cardiac muscle	0.024464813
GOTERM_BP_DIRECT	GO:2001214~positive regulation of vasculogenesis	0.024464813
GOTERM_BP_DIRECT	GO:0046033~AMP metabolic process	0.024464813
GOTERM_BP_DIRECT	GO:0007263~nitric oxide mediated signal transduction	0.024464813
GOTERM_BP_DIRECT	GO:0007155~cell adhesion	0.025106095
GOTERM_BP_DIRECT	GO:0046034~ATP metabolic process	0.026239555
GOTERM_BP_DIRECT	GO:0050870~positive regulation of T cell activation	0.026239555
GOTERM_BP_DIRECT	GO:0045840~positive regulation of mitotic nuclear division	0.026239555
GOTERM_BP_DIRECT	GO:0030500~regulation of bone mineralization	0.026239555
GOTERM_BP_DIRECT	GO:0061737~leukotriene signaling pathway	0.027043701
GOTERM_BP_DIRECT	GO:0006533~aspartate catabolic process	0.027043701
GOTERM_BP_DIRECT	GO:0035995~detection of muscle stretch	0.027043701
GOTERM_BP_DIRECT	GO:0071376~cellular response to corticotropin-releasing hormone stimulus	0.027043701
GOTERM_BP_DIRECT	GO:0006086~acetyl-CoA biosynthetic process from pyruvate	0.027043701
GOTERM_BP_DIRECT	GO:1902037~negative regulation of hematopoietic stem cell differentiation	0.027043701
GOTERM_BP_DIRECT	GO:0007599~hemostasis	0.027043701
GOTERM_BP_DIRECT	GO:0032922~circadian regulation of gene expression	0.028069589
GOTERM_BP_DIRECT	GO:0045444~fat cell differentiation	0.028734825
GOTERM_BP_DIRECT	GO:0043409~negative regulation of MAPK cascade	0.030817142
GOTERM_BP_DIRECT	GO:0072659~protein localization to plasma membrane	0.031568191
GOTERM_BP_DIRECT	GO:0071277~cellular response to calcium ion	0.031660058
GOTERM_BP_DIRECT	GO:0046330~positive regulation of JNK cascade	0.03187343
GOTERM_BP_DIRECT	GO:0071360~cellular response to exogenous dsRNA	0.03218063
GOTERM_BP_DIRECT	GO:0019886~antigen processing and presentation of exogenous peptide antigen via MHC class II	0.03218063
GOTERM_BP_DIRECT	GO:1990573~potassium ion import across plasma membrane	0.032797694
GOTERM_BP_DIRECT	GO:1902600~hydrogen ion transmembrane transport	0.032797694
GOTERM_BP_DIRECT	GO:0006836~neurotransmitter transport	0.032808983
GOTERM_BP_DIRECT	GO:0008277~regulation of G-protein coupled receptor protein signaling pathway	0.032808983
GOTERM_BP_DIRECT	GO:0009725~response to hormone	0.032808983
GOTERM_BP_DIRECT	GO:0033627~cell adhesion mediated by integrin	0.032808983
GOTERM_BP_DIRECT	GO:0030335~positive regulation of cell migration	0.033144976
GOTERM_BP_DIRECT	GO:0060347~heart trabecula formation	0.036326855
GOTERM_BP_DIRECT	GO:0006816~calcium ion transport	0.038084519
GOTERM_BP_DIRECT	GO:0008217~regulation of blood pressure	0.038084519
GOTERM_BP_DIRECT	GO:0030308~negative regulation of cell growth	0.039736429
GOTERM_BP_DIRECT	GO:0001503~ossification	0.043397921
GOTERM_BP_DIRECT	GO:0043406~positive regulation of MAP kinase activity	0.046924358

GOTERM_BP_DIRECT	GO:0007005~mitochondrion organization	0.046924358
GOTERM_BP_DIRECT	GO:0060048~cardiac muscle contraction	0.048776756
GOTERM_CC_DIRECT	GO:0030018~Z disc	1.12E-16
GOTERM_CC_DIRECT	GO:0005739~mitochondrion	3.74E-13
GOTERM_CC_DIRECT	GO:0005759~mitochondrial matrix	3.88E-13
GOTERM_CC_DIRECT	GO:0005747~mitochondrial respiratory chain complex I	2.83E-12
GOTERM_CC_DIRECT	GO:0042383~sarcolemma	1.84E-09
GOTERM_CC_DIRECT	GO:0005743~mitochondrial inner membrane	1.68E-08
GOTERM_CC_DIRECT	GO:0001725~stress fiber	9.73E-07
GOTERM_CC_DIRECT	GO:0005750~mitochondrial respiratory chain complex III	3.06E-06
GOTERM_CC_DIRECT	GO:0005751~mitochondrial respiratory chain complex IV	2.59E-05
GOTERM_CC_DIRECT	GO:0005604~basement membrane	4.09E-05
GOTERM_CC_DIRECT	GO:0031430~M band	7.11E-05
GOTERM_CC_DIRECT	GO:0016324~apical plasma membrane	8.82E-05
GOTERM_CC_DIRECT	GO:0033017~sarcoplasmic reticulum membrane	1.38E-04
GOTERM_CC_DIRECT	GO:0030315~T-tubule	1.70E-04
GOTERM_CC_DIRECT	GO:0014704~intercalated disc	2.44E-04
GOTERM_CC_DIRECT	GO:0042613~MHC class II protein complex	2.54E-04
GOTERM_CC_DIRECT	GO:0030016~myofibril	3.69E-04
GOTERM_CC_DIRECT	GO:0005737~cytoplasm	3.79E-04
GOTERM_CC_DIRECT	GO:0005887~integral component of plasma membrane	4.51E-04
GOTERM_CC_DIRECT	GO:0005967~mitochondrial pyruvate dehydrogenase complex	7.78E-04
GOTERM_CC_DIRECT	GO:0005741~mitochondrial outer membrane	9.26E-04
GOTERM_CC_DIRECT	GO:0016020~membrane	0.001136049
GOTERM_CC_DIRECT	GO:0008076~voltage-gated potassium channel complex	0.001578781
GOTERM_CC_DIRECT	GO:0015629~actin cytoskeleton	0.001895451
GOTERM_CC_DIRECT	GO:0005749~mitochondrial respiratory chain complex II, succinate dehydrogenase complex (ubiquinone)	0.002548002
GOTERM_CC_DIRECT	GO:0030424~axon	0.002574568
GOTERM_CC_DIRECT	GO:0070469~respiratory chain	0.002837285
GOTERM_CC_DIRECT	GO:0005615~extracellular space	0.003026752
GOTERM_CC_DIRECT	GO:0043209~myelin sheath	0.00339133
GOTERM_CC_DIRECT	GO:0016323~basolateral plasma membrane	0.003919182
GOTERM_CC_DIRECT	GO:0016529~sarcoplasmic reticulum	0.004591723
GOTERM_CC_DIRECT	GO:0030054~cell junction	0.005046397
GOTERM_CC_DIRECT	GO:0032982~myosin filament	0.005951935
GOTERM_CC_DIRECT	GO:0005753~mitochondrial proton-transporting ATP synthase complex	0.007459874
GOTERM_CC_DIRECT	GO:0005777~peroxisome	0.009512365
GOTERM_CC_DIRECT	GO:0005576~extracellular region	0.010179487
GOTERM_CC_DIRECT	GO:0043231~intracellular membrane-bounded organelle	0.013698381

GOTERM_CC_DIRECT	GO:0009897~external side of plasma membrane	0.018090321
GOTERM_CC_DIRECT	GO:0043034~costamere	0.01820541
GOTERM_CC_DIRECT	GO:0045121~membrane raft	0.01903771
GOTERM_CC_DIRECT	GO:0045254~pyruvate dehydrogenase complex	0.02189303
GOTERM_CC_DIRECT	GO:0009986~cell surface	0.02224632
GOTERM_CC_DIRECT	GO:0000276~mitochondrial proton-transporting ATP synthase complex, coupling factor F(o)	0.022442414
GOTERM_CC_DIRECT	GO:0005856~cytoskeleton	0.023105281
GOTERM_CC_DIRECT	GO:0031966~mitochondrial membrane	0.025604867
GOTERM_CC_DIRECT	GO:0030175~filopodium	0.028179296
GOTERM_CC_DIRECT	GO:0005923~bicellular tight junction	0.035404333
GOTERM_CC_DIRECT	GO:0005901~caveola	0.036270924
GOTERM_CC_DIRECT	GO:0031941~filamentous actin	0.039107268
GOTERM_CC_DIRECT	GO:0043197~dendritic spine	0.039361503
GOTERM_CC_DIRECT	GO:0030027~lamellipodium	0.047786761
GOTERM_MF_DIRECT	GO:0016491~oxidoreductase activity	4.44E-06
GOTERM_MF_DIRECT	GO:0044325~ion channel binding	8.41E-06
GOTERM_MF_DIRECT	GO:0008307~structural constituent of muscle	1.41E-05
GOTERM_MF_DIRECT	GO:0050660~flavin adenine dinucleotide binding	2.43E-05
GOTERM_MF_DIRECT	GO:0005509~calcium ion binding	9.56E-05
GOTERM_MF_DIRECT	GO:0042802~identical protein binding	2.37E-04
GOTERM_MF_DIRECT	GO:0005516~calmodulin binding	2.86E-04
GOTERM_MF_DIRECT	GO:0034604~pyruvate dehydrogenase (NAD+) activity	2.91E-04
GOTERM_MF_DIRECT	GO:0003779~actin binding	4.39E-04
GOTERM_MF_DIRECT	GO:0003995~acyl-CoA dehydrogenase activity	5.15E-04
GOTERM_MF_DIRECT	GO:0051539~4 iron, 4 sulfur cluster binding	5.48E-04
GOTERM_MF_DIRECT	GO:0008137~NADH dehydrogenase (ubiquinone) activity	5.76E-04
GOTERM_MF_DIRECT	GO:0046872~metal ion binding	8.70E-04
GOTERM_MF_DIRECT	GO:0051015~actin filament binding	0.001014127
GOTERM_MF_DIRECT	GO:0071949~FAD binding	0.001407582
GOTERM_MF_DIRECT	GO:0030552~cAMP binding	0.001802022
GOTERM_MF_DIRECT	GO:0009055~electron carrier activity	0.002378023
GOTERM_MF_DIRECT	GO:0005246~calcium channel regulator activity	0.002577036
GOTERM_MF_DIRECT	GO:0005515~protein binding	0.00261204
GOTERM_MF_DIRECT	GO:0031625~ubiquitin protein ligase binding	0.00318066
GOTERM_MF_DIRECT	GO:0031432~titin binding	0.003266196
GOTERM_MF_DIRECT	GO:0001228~transcriptional activator activity, RNA polymerase II transcription regulatory region sequence-specific binding	0.003395325
GOTERM_MF_DIRECT	GO:0051287~NAD binding	0.003531581
GOTERM_MF_DIRECT	GO:0008270~zinc ion binding	0.005004943
GOTERM_MF_DIRECT	GO:0005044~scavenger receptor activity	0.005326483

GOTERM_MF_DIRECT	GO:0000900~translation repressor activity, nucleic acid binding	0.005466903
GOTERM_MF_DIRECT	GO:0042609~CD4 receptor binding	0.006136277
GOTERM_MF_DIRECT	GO:0005249~voltage-gated potassium channel activity	0.006802664
GOTERM_MF_DIRECT	GO:0030170~pyridoxal phosphate binding	0.007166966
GOTERM_MF_DIRECT	GO:0046933~proton-transporting ATP synthase activity, rotational mechanism	0.007802557
GOTERM_MF_DIRECT	GO:0005154~epidermal growth factor receptor binding	0.008297933
GOTERM_MF_DIRECT	GO:0005251~delayed rectifier potassium channel activity	0.008297933
GOTERM_MF_DIRECT	GO:0051371~muscle alpha-actinin binding	0.008474763
GOTERM_MF_DIRECT	GO:0023026~MHC class II protein complex binding	0.008474763
GOTERM_MF_DIRECT	GO:0008138~protein tyrosine/serine/threonine phosphatase activity	0.008610657
GOTERM_MF_DIRECT	GO:0005319~lipid transporter activity	0.010530073
GOTERM_MF_DIRECT	GO:0008970~phosphatidylcholine 1-acylhydrolase activity	0.011462512
GOTERM_MF_DIRECT	GO:0048039~ubiquinone binding	0.011462512
GOTERM_MF_DIRECT	GO:0004115~3',5'-cyclic-AMP phosphodiesterase activity	0.012390029
GOTERM_MF_DIRECT	GO:0005518~collagen binding	0.01398503
GOTERM_MF_DIRECT	GO:0008092~cytoskeletal protein binding	0.014439363
GOTERM_MF_DIRECT	GO:0008233~peptidase activity	0.014439363
GOTERM_MF_DIRECT	GO:0003700~transcription factor activity, sequence-specific DNA binding	0.01473009
GOTERM_MF_DIRECT	GO:0004879~RNA polymerase II transcription factor activity, ligand-activated sequence-specific DNA binding	0.015100769
GOTERM_MF_DIRECT	GO:0031404~chloride ion binding	0.01874188
GOTERM_MF_DIRECT	GO:0004556~alpha-amylase activity	0.01874188
GOTERM_MF_DIRECT	GO:0050840~extracellular matrix binding	0.019942016
GOTERM_MF_DIRECT	GO:0002020~protease binding	0.02204384
GOTERM_MF_DIRECT	GO:0008135~translation factor activity, RNA binding	0.022351485
GOTERM_MF_DIRECT	GO:0004517~nitric-oxide synthase activity	0.022351485
GOTERM_MF_DIRECT	GO:0008177~succinate dehydrogenase (ubiquinone) activity	0.022351485
GOTERM_MF_DIRECT	GO:0004725~protein tyrosine phosphatase activity	0.023133867
GOTERM_MF_DIRECT	GO:0000978~RNA polymerase II core promoter proximal region sequence-specific DNA binding	0.026397633
GOTERM_MF_DIRECT	GO:0005244~voltage-gated ion channel activity	0.027766409
GOTERM_MF_DIRECT	GO:2001069~glycogen binding	0.02802729
GOTERM_MF_DIRECT	GO:0030247~polysaccharide binding	0.02802729
GOTERM_MF_DIRECT	GO:0045182~translation regulator activity	0.030313498
GOTERM_MF_DIRECT	GO:0016836~hydro-lyase activity	0.038499295
GOTERM_MF_DIRECT	GO:0004089~carbonate dehydratase activity	0.038499295
GOTERM_MF_DIRECT	GO:0005388~calcium-transporting ATPase activity	0.039307483
GOTERM_MF_DIRECT	GO:0008289~lipid binding	0.039378111

GOTERM_MF_DIRECT	GO:0051787~misfolded protein binding	0.040683117
GOTERM_MF_DIRECT	GO:0051373~FATZ binding	0.042074104
GOTERM_MF_DIRECT	GO:0004095~carnitine O-palmitoyltransferase activity	0.042074104
GOTERM_MF_DIRECT	GO:0005332~gamma-aminobutyric acid:sodium symporter activity	0.042074104
GOTERM_MF_DIRECT	GO:0003774~motor activity	0.043042962
GOTERM_MF_DIRECT	GO:0005523~tropomyosin binding	0.047822369
GOTERM_MF_DIRECT	GO:0004602~glutathione peroxidase activity	0.047822369
GOTERM_MF_DIRECT	GO:0005504~fatty acid binding	0.04830571
KEGG_PATHWAY	ssc05415:Diabetic cardiomyopathy	3.61E-33
KEGG_PATHWAY	ssc04932:Non-alcoholic fatty liver disease	6.17E-25
KEGG_PATHWAY	ssc00190:Oxidative phosphorylation	9.14E-24
KEGG_PATHWAY	ssc05208:Chemical carcinogenesis - reactive oxygen species	1.70E-23
KEGG_PATHWAY	ssc04714:Thermogenesis	3.72E-22
KEGG_PATHWAY	ssc01100:Metabolic pathways	9.02E-21
KEGG_PATHWAY	ssc05020:Prion disease	3.67E-17
KEGG_PATHWAY	ssc05012:Parkinson disease	1.33E-14
KEGG_PATHWAY	ssc05010:Alzheimer disease	9.14E-14
KEGG_PATHWAY	ssc05016:Huntington disease	1.80E-12
KEGG_PATHWAY	ssc04260:Cardiac muscle contraction	4.28E-12
KEGG_PATHWAY	ssc01200:Carbon metabolism	8.85E-11
KEGG_PATHWAY	ssc05022:Pathways of neurodegeneration - multiple diseases	2.98E-10
KEGG_PATHWAY	ssc00020:Citrate cycle (TCA cycle)	7.97E-10
KEGG_PATHWAY	ssc01210:2-Oxocarboxylic acid metabolism	2.88E-08
KEGG_PATHWAY	ssc05014:Amyotrophic lateral sclerosis	1.44E-07
KEGG_PATHWAY	ssc03320:PPAR signaling pathway	4.93E-07
KEGG_PATHWAY	ssc00280:Valine, leucine and isoleucine degradation	6.29E-07
KEGG_PATHWAY	ssc00071:Fatty acid degradation	8.65E-07
KEGG_PATHWAY	ssc04723:Retrograde endocannabinoid signaling	9.37E-07
KEGG_PATHWAY	ssc04020:Calcium signaling pathway	1.22E-06
KEGG_PATHWAY	ssc04931:Insulin resistance	2.83E-06
KEGG_PATHWAY	ssc00640:Propanoate metabolism	2.93E-06
KEGG_PATHWAY	ssc04514:Cell adhesion molecules	6.76E-06
KEGG_PATHWAY	ssc01212:Fatty acid metabolism	1.29E-05
KEGG_PATHWAY	ssc04261:Adrenergic signaling in cardiomyocytes	2.07E-05
KEGG_PATHWAY	ssc05410:Hypertrophic cardiomyopathy	2.49E-05
KEGG_PATHWAY	ssc04920:Adipocytokine signaling pathway	2.81E-05
KEGG_PATHWAY	ssc04024:cAMP signaling pathway	3.07E-05
KEGG_PATHWAY	ssc00785:Lipoic acid metabolism	1.18E-04
KEGG_PATHWAY	ssc04922:Glucagon signaling pathway	1.59E-04
KEGG_PATHWAY	ssc04066:HIF-1 signaling pathway	1.89E-04
KEGG_PATHWAY	ssc04612:Antigen processing and presentation	1.94E-04

KEGG_PATHWAY	ssc04640:Hematopoietic cell lineage	2.01E-04
KEGG_PATHWAY	ssc05412:Arrhythmogenic right ventricular cardiomyopathy	2.06E-04
KEGG_PATHWAY	ssc05323:Rheumatoid arthritis	3.74E-04
KEGG_PATHWAY	ssc00630:Glyoxylate and dicarboxylate metabolism	3.94E-04
KEGG_PATHWAY	ssc04610:Complement and coagulation cascades	4.05E-04
KEGG_PATHWAY	ssc04152:AMPK signaling pathway	4.20E-04
KEGG_PATHWAY	ssc04022:cGMP-PKG signaling pathway	4.46E-04
KEGG_PATHWAY	ssc05414:Dilated cardiomyopathy	5.01E-04
KEGG_PATHWAY	ssc04659:Th17 cell differentiation	6.46E-04
KEGG_PATHWAY	ssc04970:Salivary secretion	6.62E-04
KEGG_PATHWAY	ssc05332:Graft-versus-host disease	7.40E-04
KEGG_PATHWAY	ssc00500:Starch and sucrose metabolism	8.95E-04
KEGG_PATHWAY	ssc04933:AGE-RAGE signaling pathway in diabetic complications	9.95E-04
KEGG_PATHWAY	ssc04658:Th1 and Th2 cell differentiation	0.001008449
KEGG_PATHWAY	ssc05416:Viral myocarditis	0.00107873
KEGG_PATHWAY	ssc04068:FoxO signaling pathway	0.001085462
KEGG_PATHWAY	ssc05140:Leishmaniasis	0.00112952
KEGG_PATHWAY	ssc04380:Osteoclast differentiation	0.001212554
KEGG_PATHWAY	ssc04940:Type I diabetes mellitus	0.001391948
KEGG_PATHWAY	ssc05330:Allograft rejection	0.00145092
KEGG_PATHWAY	ssc00010:Glycolysis / Gluconeogenesis	0.001672399
KEGG_PATHWAY	ssc04924:Renin secretion	0.001740805
KEGG_PATHWAY	ssc00620:Pyruvate metabolism	0.001785511
KEGG_PATHWAY	ssc04919:Thyroid hormone signaling pathway	0.001887426
KEGG_PATHWAY	ssc01230:Biosynthesis of amino acids	0.002712106
KEGG_PATHWAY	ssc05418:Fluid shear stress and atherosclerosis	0.002775122
KEGG_PATHWAY	ssc04973:Carbohydrate digestion and absorption	0.003806173
KEGG_PATHWAY	ssc04710:Circadian rhythm	0.004414902
KEGG_PATHWAY	ssc04010:MAPK signaling pathway	0.004818006
KEGG_PATHWAY	ssc05310:Asthma	0.005641317
KEGG_PATHWAY	ssc00650:Butanoate metabolism	0.007107439
KEGG_PATHWAY	ssc05200:Pathways in cancer	0.007167812
KEGG_PATHWAY	ssc05169:Epstein-Barr virus infection	0.008061349
KEGG_PATHWAY	ssc05322:Systemic lupus erythematosus	0.008131042
KEGG_PATHWAY	ssc05320:Autoimmune thyroid disease	0.008484545
KEGG_PATHWAY	ssc00220:Arginine biosynthesis	0.008734138
KEGG_PATHWAY	ssc04910:Insulin signaling pathway	0.00892909
KEGG_PATHWAY	ssc05321:Inflammatory bowel disease	0.009416411
KEGG_PATHWAY	ssc04213:Longevity regulating pathway - multiple species	0.009715838
KEGG_PATHWAY	ssc04512:ECM-receptor interaction	0.010016722
KEGG_PATHWAY	ssc00410:beta-Alanine metabolism	0.010375807
KEGG_PATHWAY	ssc05145:Toxoplasmosis	0.01052038

KEGG_PATHWAY	ssc05164:Influenza A	0.0108784
KEGG_PATHWAY	ssc04972:Pancreatic secretion	0.011394878
KEGG_PATHWAY	ssc04921:Oxytocin signaling pathway	0.013193369
KEGG_PATHWAY	ssc00910:Nitrogen metabolism	0.013722611
KEGG_PATHWAY	ssc04964:Proximal tubule bicarbonate reclamation	0.014273516
KEGG_PATHWAY	ssc04672:Intestinal immune network for IgA production	0.017483627
KEGG_PATHWAY	ssc04145:Phagosome	0.018219517
KEGG_PATHWAY	ssc00230:Purine metabolism	0.019052816
KEGG_PATHWAY	ssc04926:Relaxin signaling pathway	0.019052816
KEGG_PATHWAY	ssc04371:Apelin signaling pathway	0.020112748
KEGG_PATHWAY	ssc00350:Tyrosine metabolism	0.020591789
KEGG_PATHWAY	ssc04670:Leukocyte transendothelial migration	0.023138694
KEGG_PATHWAY	ssc05150:Staphylococcus aureus infection	0.026006463
KEGG_PATHWAY	ssc00051:Fructose and mannose metabolism	0.027810104
KEGG_PATHWAY	ssc00062:Fatty acid elongation	0.031818229
KEGG_PATHWAY	ssc05162:Measles	0.03288467
KEGG_PATHWAY	ssc04137:Mitophagy - animal	0.034104944
KEGG_PATHWAY	ssc05166:Human T-cell leukemia virus 1 infection	0.035212922
KEGG_PATHWAY	ssc00360:Phenylalanine metabolism	0.035476316
KEGG_PATHWAY	ssc04923:Regulation of lipolysis in adipocytes	0.036266771
KEGG_PATHWAY	ssc04510:Focal adhesion	0.038530617
KEGG_PATHWAY	ssc04625:C-type lectin receptor signaling pathway	0.038625587
KEGG_PATHWAY	ssc05165:Human papillomavirus infection	0.040135432
KEGG_PATHWAY	ssc04936:Alcoholic liver disease	0.041561248
KEGG_PATHWAY	ssc04971:Gastric acid secretion	0.04510831
KEGG_PATHWAY	ssc05146:Amoebiasis	0.046200108
KEGG_PATHWAY	ssc00330:Arginine and proline metabolism	0.048743479
KEGG_PATHWAY	ssc05230:Central carbon metabolism in cancer	0.049686297

E100＞E70

Category	Term	PValue
GOTERM_BP_DIRECT	GO:0032981~mitochondrial respiratory chain complex I assembly	1.24E-14
GOTERM_BP_DIRECT	GO:0006099~tricarboxylic acid cycle	6.30E-13
GOTERM_BP_DIRECT	GO:0006120~mitochondrial electron transport, NADH to ubiquinone	1.66E-08
GOTERM_BP_DIRECT	GO:0033539~fatty acid beta-oxidation using acyl-CoA dehydrogenase	5.21E-06
GOTERM_BP_DIRECT	GO:0006122~mitochondrial electron transport, ubiquinol to cytochrome c	7.25E-06
GOTERM_BP_DIRECT	GO:0022904~respiratory electron transport chain	6.82E-05

GOTERM_BP_DIRECT	GO:0006103~2-oxoglutarate metabolic process	1.08E-04
GOTERM_BP_DIRECT	GO:0034765~regulation of ion transmembrane transport	0.001313447
GOTERM_BP_DIRECT	GO:0006123~mitochondrial electron transport, cytochrome c to oxygen	0.001337258
GOTERM_BP_DIRECT	GO:0009060~aerobic respiration	0.001409832
GOTERM_BP_DIRECT	GO:0072593~reactive oxygen species metabolic process	0.0021319
GOTERM_BP_DIRECT	GO:1900016~negative regulation of cytokine production involved in inflammatory response	0.002338936
GOTERM_BP_DIRECT	GO:0071456~cellular response to hypoxia	0.002346722
GOTERM_BP_DIRECT	GO:0001525~angiogenesis	0.002400152
GOTERM_BP_DIRECT	GO:0071805~potassium ion transmembrane transport	0.002758282
GOTERM_BP_DIRECT	GO:0034138~toll-like receptor 3 signaling pathway	0.005153799
GOTERM_BP_DIRECT	GO:0035458~cellular response to interferon-beta	0.005203563
GOTERM_BP_DIRECT	GO:0007005~mitochondrion organization	0.005433675
GOTERM_BP_DIRECT	GO:0006006~glucose metabolic process	0.006179303
GOTERM_BP_DIRECT	GO:0009409~response to cold	0.006429035
GOTERM_BP_DIRECT	GO:0032728~positive regulation of interferon-beta production	0.006660253
GOTERM_BP_DIRECT	GO:1902600~hydrogen ion transmembrane transport	0.00781766
GOTERM_BP_DIRECT	GO:0032922~circadian regulation of gene expression	0.007828119
GOTERM_BP_DIRECT	GO:0006809~nitric oxide biosynthetic process	0.007905238
GOTERM_BP_DIRECT	GO:0008637~apoptotic mitochondrial changes	0.007905238
GOTERM_BP_DIRECT	GO:0010940~positive regulation of necrotic cell death	0.009031955
GOTERM_BP_DIRECT	GO:0006086~acetyl-CoA biosynthetic process from pyruvate	0.009031955
GOTERM_BP_DIRECT	GO:0006533~aspartate catabolic process	0.009031955
GOTERM_BP_DIRECT	GO:2000379~positive regulation of reactive oxygen species metabolic process	0.012900167

GOTERM_BP_DIRECT	GO:0042632~cholesterol homeostasis	0.014880642
GOTERM_BP_DIRECT	GO:0014898~cardiac muscle hypertrophy in response to stress	0.01557444
GOTERM_BP_DIRECT	GO:0045840~positive regulation of mitotic nuclear division	0.015866232
GOTERM_BP_DIRECT	GO:0015990~electron transport coupled proton transport	0.017394875
GOTERM_BP_DIRECT	GO:0006531~aspartate metabolic process	0.017394875
GOTERM_BP_DIRECT	GO:0003081~regulation of systemic arterial blood pressure by renin-angiotensin	0.017394875
GOTERM_BP_DIRECT	GO:0006121~mitochondrial electron transport, succinate to ubiquinone	0.017394875
GOTERM_BP_DIRECT	GO:0006104~succinyl-CoA metabolic process	0.017394875
GOTERM_BP_DIRECT	GO:0009063~cellular amino acid catabolic process	0.017394875
GOTERM_BP_DIRECT	GO:0045785~positive regulation of cell adhesion	0.018198066
GOTERM_BP_DIRECT	GO:0045766~positive regulation of angiogenesis	0.018539934
GOTERM_BP_DIRECT	GO:0051607~defense response to virus	0.0187057
GOTERM_BP_DIRECT	GO:0008154~actin polymerization or depolymerization	0.019227092
GOTERM_BP_DIRECT	GO:0009968~negative regulation of signal transduction	0.019227092
GOTERM_BP_DIRECT	GO:0009725~response to hormone	0.019227092
GOTERM_BP_DIRECT	GO:0045595~regulation of cell differentiation	0.019227092
GOTERM_BP_DIRECT	GO:0006633~fatty acid biosynthetic process	0.020233293
GOTERM_BP_DIRECT	GO:0008217~regulation of blood pressure	0.022713408
GOTERM_BP_DIRECT	GO:0006635~fatty acid beta-oxidation	0.022713408
GOTERM_BP_DIRECT	GO:0031623~receptor internalization	0.024079432
GOTERM_BP_DIRECT	GO:0010875~positive regulation of cholesterol efflux	0.027181977
GOTERM_BP_DIRECT	GO:0032496~response to lipopolysaccharide	0.027381879
GOTERM_BP_DIRECT	GO:0045600~positive regulation of fat cell differentiation	0.027429274

GOTERM_BP_DIRECT	GO:0007219~Notch signaling pathway	0.027556284
GOTERM_BP_DIRECT	GO:0071356~cellular response to tumor necrosis factor	0.027912191
GOTERM_BP_DIRECT	GO:0006874~cellular calcium ion homeostasis	0.027912191
GOTERM_BP_DIRECT	GO:1901098~positive regulation of autophagosome maturation	0.027922896
GOTERM_BP_DIRECT	GO:0010745~negative regulation of macrophage derived foam cell differentiation	0.027922896
GOTERM_BP_DIRECT	GO:0050873~brown fat cell differentiation	0.031793742
GOTERM_BP_DIRECT	GO:0050728~negative regulation of inflammatory response	0.032751982
GOTERM_BP_DIRECT	GO:0010942~positive regulation of cell death	0.03275553
GOTERM_BP_DIRECT	GO:0030335~positive regulation of cell migration	0.033525397
GOTERM_BP_DIRECT	GO:0015986~ATP synthesis coupled proton transport	0.036835916
GOTERM_BP_DIRECT	GO:0006936~muscle contraction	0.039191647
GOTERM_BP_DIRECT	GO:0051014~actin filament severing	0.039997497
GOTERM_BP_DIRECT	GO:1990845~adaptive thermogenesis	0.040348145
GOTERM_BP_DIRECT	GO:0006102~isocitrate metabolic process	0.040348145
GOTERM_BP_DIRECT	GO:0001768~establishment of T cell polarity	0.040348145
GOTERM_BP_DIRECT	GO:0019227~neuronal action potential propagation	0.040348145
GOTERM_BP_DIRECT	GO:0042773~ATP synthesis coupled electron transport	0.040348145
GOTERM_BP_DIRECT	GO:0045746~negative regulation of Notch signaling pathway	0.042311059
GOTERM_BP_DIRECT	GO:0071260~cellular response to mechanical stimulus	0.043698428
GOTERM_BP_DIRECT	GO:0007189~adenylate cyclase-activating G-protein coupled receptor signaling pathway	0.046202076
GOTERM_BP_DIRECT	GO:0060339~negative regulation of type I interferon-mediated signaling pathway	0.047975007
GOTERM_BP_DIRECT	GO:0010906~regulation of glucose metabolic process	0.047975007
GOTERM_BP_DIRECT	GO:0034142~toll-like receptor 4 signaling pathway	0.047975007

GOTERM_BP_DIRECT	GO:0051482~positive regulation of cytosolic calcium ion concentration involved in phospholipase C-activating G-protein coupled signaling pathway	0.048219398
GOTERM_CC_DIRECT	GO:0005747~mitochondrial respiratory chain complex I	6.49E-19
GOTERM_CC_DIRECT	GO:0005743~mitochondrial inner membrane	2.23E-15
GOTERM_CC_DIRECT	GO:0005759~mitochondrial matrix	3.50E-15
GOTERM_CC_DIRECT	GO:0005739~mitochondrion	1.74E-14
GOTERM_CC_DIRECT	GO:0005750~mitochondrial respiratory chain complex III	3.63E-08
GOTERM_CC_DIRECT	GO:0005751~mitochondrial respiratory chain complex IV	1.72E-06
GOTERM_CC_DIRECT	GO:0070469~respiratory chain	7.05E-06
GOTERM_CC_DIRECT	GO:0008076~voltage-gated potassium channel complex	2.64E-05
GOTERM_CC_DIRECT	GO:0005967~mitochondrial pyruvate dehydrogenase complex	8.29E-05
GOTERM_CC_DIRECT	GO:0016323~basolateral plasma membrane	1.95E-04
GOTERM_CC_DIRECT	GO:0005749~mitochondrial respiratory chain complex II, succinate dehydrogenase complex (ubiquinone)	4.67E-04
GOTERM_CC_DIRECT	GO:0005887~integral component of plasma membrane	8.15E-04
GOTERM_CC_DIRECT	GO:0005741~mitochondrial outer membrane	0.00118537
GOTERM_CC_DIRECT	GO:0031966~mitochondrial membrane	0.001272972
GOTERM_CC_DIRECT	GO:0043231~intracellular membrane-bounded organelle	0.001767119
GOTERM_CC_DIRECT	GO:0005737~cytoplasm	0.002929187
GOTERM_CC_DIRECT	GO:0030016~myofibril	0.003840978
GOTERM_CC_DIRECT	GO:0042383~sarcolemma	0.004835282
GOTERM_CC_DIRECT	GO:0005753~mitochondrial proton-transporting ATP synthase complex	0.005268936
GOTERM_CC_DIRECT	GO:0016324~apical plasma membrane	0.005913963
GOTERM_CC_DIRECT	GO:0045121~membrane raft	0.006963492

GOTERM_CC_DIRECT	GO:0045254~pyruvate dehydrogenase complex	0.007114575
GOTERM_CC_DIRECT	GO:0045261~proton-transporting ATP synthase complex, catalytic core F(1)	0.008132651
GOTERM_CC_DIRECT	GO:0009897~external side of plasma membrane	0.009089545
GOTERM_CC_DIRECT	GO:0043235~receptor complex	0.012234318
GOTERM_CC_DIRECT	GO:0001725~stress fiber	0.014844376
GOTERM_CC_DIRECT	GO:0032982~myosin filament	0.022187285
GOTERM_CC_DIRECT	GO:0045252~oxoglutarate dehydrogenase complex	0.022187285
GOTERM_CC_DIRECT	GO:0005615~extracellular space	0.0228264
GOTERM_CC_DIRECT	GO:0030018~Z disc	0.025643703
GOTERM_CC_DIRECT	GO:0015629~actin cytoskeleton	0.028055235
GOTERM_CC_DIRECT	GO:0005865~striated muscle thin filament	0.032197959
GOTERM_CC_DIRECT	GO:0033017~sarcoplasmic reticulum membrane	0.035304786
GOTERM_CC_DIRECT	GO:0014704~intercalated disc	0.037538936
GOTERM_CC_DIRECT	GO:0005604~basement membrane	0.044256588
GOTERM_MF_DIRECT	GO:0008137~NADH dehydrogenase (ubiquinone) activity	2.57E-09
GOTERM_MF_DIRECT	GO:0051287~NAD binding	6.61E-06
GOTERM_MF_DIRECT	GO:0005249~voltage-gated potassium channel activity	7.09E-06
GOTERM_MF_DIRECT	GO:0034604~pyruvate dehydrogenase (NAD+) activity	2.88E-05
GOTERM_MF_DIRECT	GO:0016491~oxidoreductase activity	3.78E-05
GOTERM_MF_DIRECT	GO:0009055~electron carrier activity	4.50E-05
GOTERM_MF_DIRECT	GO:0050660~flavin adenine dinucleotide binding	5.22E-05
GOTERM_MF_DIRECT	GO:0048039~ubiquinone binding	8.29E-05
GOTERM_MF_DIRECT	GO:0005251~delayed rectifier potassium channel activity	4.15E-04

GOTERM_MF_DIRECT	GO:0003995~acyl-CoA dehydrogenase activity	6.17E-04
GOTERM_MF_DIRECT	GO:0051539~4 iron, 4 sulfur cluster binding	6.57E-04
GOTERM_MF_DIRECT	GO:0005319~lipid transporter activity	8.16E-04
GOTERM_MF_DIRECT	GO:0070888~E-box binding	0.002346767
GOTERM_MF_DIRECT	GO:0001228~transcriptional activator activity, RNA polymerase II transcription regulatory region sequence-specific binding	0.002872168
GOTERM_MF_DIRECT	GO:0004556~alpha-amylase activity	0.003651467
GOTERM_MF_DIRECT	GO:0046872~metal ion binding	0.004159179
GOTERM_MF_DIRECT	GO:0046933~proton-transporting ATP synthase activity, rotational mechanism	0.005268901
GOTERM_MF_DIRECT	GO:0005516~calmodulin binding	0.00531125
GOTERM_MF_DIRECT	GO:0005244~voltage-gated ion channel activity	0.005945242
GOTERM_MF_DIRECT	GO:0008177~succinate dehydrogenase (ubiquinone) activity	0.007115868
GOTERM_MF_DIRECT	GO:0005509~calcium ion binding	0.012593202
GOTERM_MF_DIRECT	GO:0004879~RNA polymerase II transcription factor activity, ligand-activated sequence-specific DNA binding	0.013694083
GOTERM_MF_DIRECT	GO:0030170~pyridoxal phosphate binding	0.016656931
GOTERM_MF_DIRECT	GO:0044325~ion channel binding	0.021417583
GOTERM_MF_DIRECT	GO:0004957~prostaglandin E receptor activity	0.02219146
GOTERM_MF_DIRECT	GO:0045028~G-protein coupled purinergic nucleotide receptor activity	0.02388461
GOTERM_MF_DIRECT	GO:0042802~identical protein binding	0.028433193
GOTERM_MF_DIRECT	GO:0051537~2 iron, 2 sulfur cluster binding	0.028833324
GOTERM_MF_DIRECT	GO:0005223~intracellular cGMP activated cation channel activity	0.03220411
GOTERM_MF_DIRECT	GO:0031625~ubiquitin protein ligase binding	0.032349766
GOTERM_MF_DIRECT	GO:0008270~zinc ion binding	0.032918724

GOTERM_MF_DIRECT	GO:0004725~protein tyrosine phosphatase activity	0.035287637
GOTERM_MF_DIRECT	GO:0042803~protein homodimerization activity	0.037046905
GOTERM_MF_DIRECT	GO:0004602~glutathione peroxidase activity	0.041900817
GOTERM_MF_DIRECT	GO:0005523~tropomyosin binding	0.041900817
GOTERM_MF_DIRECT	GO:0005080~protein kinase C binding	0.042403296
GOTERM_MF_DIRECT	GO:0031404~chloride ion binding	0.043625167
GOTERM_MF_DIRECT	GO:0005222~intracellular cAMP activated cation channel activity	0.043625167
GOTERM_MF_DIRECT	GO:0008467~[heparan sulfate]-glucosamine 3-sulfotransferase 1 activity	0.043625167
GOTERM_MF_DIRECT	GO:0001223~transcription coactivator binding	0.047601082
GOTERM_MF_DIRECT	GO:0016747~transferase activity, transferring acyl groups other than amino-acyl groups	0.04906024
GOTERM_MF_DIRECT	GO:0004622~lysophospholipase activity	0.04906024
KEGG_PATHWAY	ssc05415:Diabetic cardiomyopathy	1.98E-42
KEGG_PATHWAY	ssc00190:Oxidative phosphorylation	1.71E-31
KEGG_PATHWAY	ssc04714:Thermogenesis	4.63E-28
KEGG_PATHWAY	ssc01100:Metabolic pathways	1.45E-26
KEGG_PATHWAY	ssc04932:Non-alcoholic fatty liver disease	5.30E-26
KEGG_PATHWAY	ssc05208:Chemical carcinogenesis - reactive oxygen species	1.95E-24
KEGG_PATHWAY	ssc05020:Prion disease	3.49E-20
KEGG_PATHWAY	ssc05012:Parkinson disease	8.36E-20
KEGG_PATHWAY	ssc05016:Huntington disease	9.31E-19
KEGG_PATHWAY	ssc05010:Alzheimer disease	1.70E-17
KEGG_PATHWAY	ssc00020:Citrate cycle (TCA cycle)	4.39E-16
KEGG_PATHWAY	ssc05014:Amyotrophic lateral sclerosis	1.85E-14
KEGG_PATHWAY	ssc05022:Pathways of neurodegeneration - multiple diseases	3.19E-14
KEGG_PATHWAY	ssc01200:Carbon metabolism	2.81E-13
KEGG_PATHWAY	ssc04723:Retrograde endocannabinoid signaling	1.10E-12
KEGG_PATHWAY	ssc04260:Cardiac muscle contraction	2.36E-10
KEGG_PATHWAY	ssc01210:2-Oxocarboxylic acid metabolism	3.88E-09
KEGG_PATHWAY	ssc03320:PPAR signaling pathway	1.09E-06
KEGG_PATHWAY	ssc00071:Fatty acid degradation	2.69E-06
KEGG_PATHWAY	ssc04024:cAMP signaling pathway	8.26E-06
KEGG_PATHWAY	ssc04931:Insulin resistance	1.01E-05
KEGG_PATHWAY	ssc04020:Calcium signaling pathway	9.98E-05
KEGG_PATHWAY	ssc04964:Proximal tubule bicarbonate reclamation	1.38E-04

KEGG_PATHWAY	ssc04261:Adrenergic signaling in cardiomyocytes	2.41E-04
KEGG_PATHWAY	ssc01212:Fatty acid metabolism	2.76E-04
KEGG_PATHWAY	ssc04022:cGMP-PKG signaling pathway	4.22E-04
KEGG_PATHWAY	ssc00620:Pyruvate metabolism	5.27E-04
KEGG_PATHWAY	ssc00280:Valine, leucine and isoleucine degradation	5.36E-04
KEGG_PATHWAY	ssc04920:Adipocytokine signaling pathway	6.08E-04
KEGG_PATHWAY	ssc00630:Glyoxylate and dicarboxylate metabolism	0.001369931
KEGG_PATHWAY	ssc04922:Glucagon signaling pathway	0.001468607
KEGG_PATHWAY	ssc04924:Renin secretion	0.001482669
KEGG_PATHWAY	ssc00640:Propanoate metabolism	0.001670055
KEGG_PATHWAY	ssc01230:Biosynthesis of amino acids	0.002110713
KEGG_PATHWAY	ssc00785:Lipoic acid metabolism	0.002553254
KEGG_PATHWAY	ssc04972:Pancreatic secretion	0.003481488
KEGG_PATHWAY	ssc00220:Arginine biosynthesis	0.004209097
KEGG_PATHWAY	ssc04970:Salivary secretion	0.004331883
KEGG_PATHWAY	ssc04919:Thyroid hormone signaling pathway	0.007239927
KEGG_PATHWAY	ssc04066:HIF-1 signaling pathway	0.007909008
KEGG_PATHWAY	ssc04710:Circadian rhythm	0.012323716
KEGG_PATHWAY	ssc04923:Regulation of lipolysis in adipocytes	0.012345327
KEGG_PATHWAY	ssc00230:Purine metabolism	0.013338138
KEGG_PATHWAY	ssc04610:Complement and coagulation cascades	0.013703114
KEGG_PATHWAY	ssc04973:Carbohydrate digestion and absorption	0.015104127
KEGG_PATHWAY	ssc04979:Cholesterol metabolism	0.020776995
KEGG_PATHWAY	ssc02010:ABC transporters	0.022958414
KEGG_PATHWAY	ssc04146:Peroxisome	0.023539769
KEGG_PATHWAY	ssc04936:Alcoholic liver disease	0.025476498
KEGG_PATHWAY	ssc04926:Relaxin signaling pathway	0.028978797
KEGG_PATHWAY	ssc05410:Hypertrophic cardiomyopathy	0.029027085
KEGG_PATHWAY	ssc00270:Cysteine and methionine metabolism	0.030438158
KEGG_PATHWAY	ssc05032:Morphine addiction	0.033154968
KEGG_PATHWAY	ssc00500:Starch and sucrose metabolism	0.03968327
KEGG_PATHWAY	ssc04152:AMPK signaling pathway	0.041078723
KEGG_PATHWAY	ssc05418:Fluid shear stress and atherosclerosis	0.048530928
