# Supplementary material for: Dynamic changes in chromatin accessibility and gene expression involved in fetal myogenesis of Min pigs
Source: Anim Biosci. 2025 May 12;38(11):2525–36. doi: 10.5713/ab.25.0034 (PMC12580940; doi:10.5713/ab.25.0034)
Supplement: Supplementary file 5 [file ab-25-0034-supplementary-5.pdf]

Supplementary File 5. Gene list for Figure 4B.

Energy metabolism	
Ensembl Gene ID	Gene Symbol
ENSSSCG00000040199	AFG1L
ENSSSCG00000005528	NDUFA8
ENSSSCG00000004927	CLPX
ENSSSCG00000004596	LIPC
ENSSSCG00000004193	ENPP1
ENSSSCG00000004576	RORA
ENSSSCG00000005627	AK1
ENSSSCG00000005636	SLC25A25
ENSSSCG00000056902	CAV3
ENSSSCG00000011698	GYG1
ENSSSCG00000030413	PIK3CA
ENSSSCG00000011831	APOD
ENSSSCG00000025881	PDHB
ENSSSCG00000011477	ACOX2
ENSSSCG00000031913	NDUFB4
ENSSSCG00000011356	UQCRC1
ENSSSCG00000003776	ACADM
ENSSSCG00000031450	MLYCD
ENSSSCG00000029034	COX4I1
ENSSSCG00000036145	PRKAA2
ENSSSCG00000003851	CPT2
ENSSSCG00000060225	CIDEA
ENSSSCG00000038826	NDUFV2
ENSSSCG00000003903	UQCRH
ENSSSCG00000027085	LOC100522678
ENSSSCG00000025188	LEPR
ENSSSCG00000003812	EFCAB7; PGM1
ENSSSCG00000037792	COX7A1
ENSSSCG00000003229	ETFB
ENSSSCG00000020926	SDHB
ENSSSCG00000003506	PINK1
ENSSSCG00000003154	GYS1
ENSSSCG00000040325	COX7C
ENSSSCG00000013354	CSRP3
ENSSSCG00000014289	UQCRQ
ENSSSCG00000014371	NDUFA2
ENSSSCG00000013778	NDUFB7
ENSSSCG00000032081	NDUFS3
ENSSSCG00000061991	COX8H
ENSSSCG00000012880	CPT1A

ENSSSCG00000012841	PNPLA2
ENSSSCG00000013513	LRG1; PLIN5
ENSSSCG00000013436	UQCR11
ENSSSCG00000024144	NDUFS7
ENSSSCG00000035532	ATP5F1D
ENSSSCG00000037455	NDUFS8
ENSSSCG00000027307	NDUFA11
ENSSSCG00000013022	PYGM
ENSSSCG00000046640	-
ENSSSCG00000040631	LPL
ENSSSCG00000061436	ACACB
ENSSSCG00000010464	PPP1R3C
ENSSSCG00000024550	UQCR10
ENSSSCG00000009916	ACADS
ENSSSCG00000016156	ACADL
ENSSSCG00000016349	NDUFA10
ENSSSCG00000016127	NDUFS1
ENSSSCG00000016100	NDUFB3
ENSSSCG00000015030	DLAT
ENSSSCG00000015034	SDHD
ENSSSCG00000015334	PDK4
ENSSSCG00000026153	ACAT1
ENSSSCG00000015405	LOC100511343
ENSSSCG00000061957	CD36
ENSSSCG00000025187	TMEM126A
ENSSSCG00000029275	PPARGC1A
ENSSSCG00000008888	NPY1R
ENSSSCG00000009150	HADH
ENSSSCG00000008877	ETFDH
ENSSSCG00000008261	HK2
ENSSSCG00000008569	HADHB
ENSSSCG00000008571	HADHA
ENSSSCG00000008247	SUCLG1
ENSSSCG00000008195	COX5B
ENSSSCG00000025486	MDH2
ENSSSCG00000007744	PHKG1
ENSSSCG00000036789	COX6A2
ENSSSCG00000032556	ALDOA
ENSSSCG00000030328	NDUFB10
ENSSSCG00000037846	ACOXL
ENSSSCG00000026453	ACSM5
ENSSSCG00000010795	NDUFAB1
ENSSSCG00000030033	ACSM4

ENSSSCG00000025751	MDH1
ENSSSCG00000055783	UQCRB
ENSSSCG00000006126	DECR1
ENSSSCG00000005976	NDUFB9
ENSSSCG00000031918	CYC1
ENSSSCG00000006872	-
ENSSSCG00000006703	PRKAB2
ENSSSCG00000030318	SDHC
ENSSSCG00000006358	NDUFS2
ENSSSCG00000006296	ATP1B1
ENSSSCG00000053432	NDUFA1
ENSSSCG00000034487	NDUFB11
ENSSSCG00000023643	PDHA1
ENSSSCG00000032498	IDH3A
ENSSSCG00000002374	DLST
ENSSSCG00000002007	FITM1
ENSSSCG00000002029	LOC100736765
ENSSSCG00000001862	ETFA
ENSSSCG00000001852	IDH2
ENSSSCG00000000997	PPP1R3G
ENSSSCG00000024316	COX5A
ENSSSCG00000021129	PFKM
ENSSSCG00000000905	NDUFA12
ENSSSCG00000022364	CPT1B
ENSSSCG00000000719	NDUFA9
ENSSSCG00000000682	GNB3
ENSSSCG00000000694	GAPDH
ENSSSCG00000000064	ACO2
ENSSSCG00000033843	ETFRF1
ENSSSCG00000035686	CS
ENSSSCG00000020686	SDHA
ENSSSCG00000009405	SUCLA2
ENSSSCG00000011003	NDUFB6
ENSSSCG00000038007	HACD1
ENSSSCG00000007173	IDH3B
ENSSSCG00000017406	STAT5B
ENSSSCG00000036933	NR1D1
ENSSSCG00000017904	ENO3
ENSSSCG00000031851	CBR2
ENSSSCG00000039873	DCXR
ENSSSCG00000017947	ACADVL
ENSSSCG00000023915	SLC2A4
ENSSSCG00000016720	PGAM2

ENSSSCG00000016714	CYCS
ENSSSCG00000016751	GCK; MYL7
ENSSSCG00000016743	OGDH
ENSSSCG00000040464	LEP
ENSSSCG00000016634	CAV1
ENSSSCG00000037869	NDUFA5
ENSSSCG00000032390	NDUFB2
ENSSSCG00000018087	ND4
ENSSSCG00000018091	ND5
ENSSSCG00000018092	ND6

Mitosis

Ensembl Gene ID	Gene Symbol
ENSSSCG00000004241	GJA1
ENSSSCG00000004969	KIF23
ENSSSCG00000004423	TUBE1
ENSSSCG00000005587	NEK6
ENSSSCG00000004518	SKA1
ENSSSCG00000004466	TTK
ENSSSCG00000004225	TPD52L1
ENSSSCG00000004588	CCNB2
ENSSSCG00000057184	CENPW
ENSSSCG00000004170	-
ENSSSCG00000005052	WDHD1
ENSSSCG00000005056	DLGAP5
ENSSSCG00000004782	BUB1B
ENSSSCG00000035544	NUSAP1
ENSSSCG00000042522	KNL1
ENSSSCG00000011624	MCM2
ENSSSCG00000011527	CNTN4
ENSSSCG00000034229	MIS18A
ENSSSCG00000011207	SGO1
ENSSSCG00000023503	PRKCD
ENSSSCG00000002768	CENPT
ENSSSCG00000003768	NEXN
ENSSSCG00000002651	CDT1
ENSSSCG00000024800	DSC2
ENSSSCG00000023376	PARD6G
ENSSSCG00000003861	ORC1
ENSSSCG00000032909	CDCA8
ENSSSCG00000038992	TUBB6
ENSSSCG00000059665	CEP192
ENSSSCG00000003949	CDC20

ENSSSCG00000003888	STIL
ENSSSCG00000002855	CCNE1
ENSSSCG00000025652	CDH1
ENSSSCG00000023162	CDH3
ENSSSCG00000003697	NDC80
ENSSSCG00000002923	WDR62
ENSSSCG00000026257	STMN1
ENSSSCG00000003403	APITD1
ENSSSCG00000013332	KIF18A
ENSSSCG00000013411	WEE1
ENSSSCG00000014324	MYOT
ENSSSCG00000014326	KIF20A
ENSSSCG00000051455	CDC25C
ENSSSCG00000024954	FGF1
ENSSSCG00000014214	TRIM36
ENSSSCG00000033444	SPC24
ENSSSCG00000014039	RGS14
ENSSSCG00000013425	MISP
ENSSSCG00000013249	CKAP5
ENSSSCG00000013260	MDK
ENSSSCG00000013509	CHAF1A
ENSSSCG00000037337	BRSK2
ENSSSCG00000013517	UHRF1
ENSSSCG00000013066	INCENP
ENSSSCG00000013008	CDCA5
ENSSSCG00000027984	ZWINT
ENSSSCG00000009670	ESCO2
ENSSSCG00000010214	CDK1
ENSSSCG00000009839	CIT
ENSSSCG00000010122	CDC45
ENSSSCG00000028559	LZTS1
ENSSSCG00000021161	CKS2
ENSSSCG00000052117	LOC100510930; LOC100155138
ENSSSCG00000052017	LOC100155138
ENSSSCG00000009653	CDCA2
ENSSSCG00000026302	MKI67
ENSSSCG00000010471	KIF11
ENSSSCG00000034864	CHEK2
ENSSSCG00000010477	CEP55
ENSSSCG00000033051	POLE
ENSSSCG00000039546	KNTC1
ENSSSCG00000035564	MCM6
ENSSSCG00000016092	SGO2

ENSSSCG00000015924	SPC25
ENSSSCG00000015961	CDCA7
ENSSSCG00000015215	CHEK1
ENSSSCG00000015581	CENPF
ENSSSCG00000015604	NEK2
ENSSSCG00000008747	NCAPG
ENSSSCG00000036256	MAD2L1
ENSSSCG00000009134	EGF
ENSSSCG00000009031	EDNRA
ENSSSCG00000058425	FGF5
ENSSSCG00000038185	EREG
ENSSSCG00000008965	BTC
ENSSSCG00000008677	TACC3
ENSSSCG00000008697	HTT
ENSSSCG00000038543	CENPA
ENSSSCG00000008538	SPDYA
ENSSSCG00000040479	NDE1
ENSSSCG00000030469	BUB1
ENSSSCG00000008125	NCAPH
ENSSSCG00000026748	PLK1
ENSSSCG00000005965	MYC
ENSSSCG00000047247	DSCC1
ENSSSCG00000006095	CCNE2
ENSSSCG00000025980	CHMP4C
ENSSSCG00000006474	NES
ENSSSCG00000006273	MCM4
ENSSSCG00000036975	PSRC1
ENSSSCG00000006333	NUF2
ENSSSCG00000012377	KIF4A
ENSSSCG00000029771	PCDH11X
ENSSSCG00000012307	CCNB3
ENSSSCG00000025488	MCM3
ENSSSCG00000040355	TICRR
ENSSSCG00000033655	TUBB2B
ENSSSCG00000035598	EDN1
ENSSSCG00000041322	ALPK3
ENSSSCG00000037307	PRC1
ENSSSCG00000001379	TUBB
ENSSSCG00000001510	KIFC1
ENSSSCG00000000182	WNT10B
ENSSSCG00000000265	ESPL1
ENSSSCG00000000217	RACGAP1
ENSSSCG00000000190	TUBA1B

ENSSSCG00000000194	LOC100127131
ENSSSCG00000061347	TUBA1A
ENSSSCG00000038694	CCND2
ENSSSCG00000033327	-
ENSSSCG00000031361	LOC102159820
ENSSSCG00000000683	CDCA3
ENSSSCG00000000739	FOXM1
ENSSSCG00000025092	CDK4
ENSSSCG00000037597	CDK2
ENSSSCG00000025965	SPDL1
ENSSSCG00000016878	FGF10
ENSSSCG00000016835	SKP2
ENSSSCG00000030186	KIF2A
ENSSSCG00000029326	CCNB1
ENSSSCG00000027147	CENPH
ENSSSCG00000017032	PTTG1
ENSSSCG00000034522	TRIP13
ENSSSCG00000009434	RGCC
ENSSSCG00000025120	BORA
ENSSSCG00000024635	SPART
ENSSSCG00000009378	CKAP2
ENSSSCG00000031955	PCDH20
ENSSSCG00000029393	CENPJ
ENSSSCG00000028249	SKA3
ENSSSCG00000009278	FGF9
ENSSSCG00000010816	TGFB2
ENSSSCG00000037241	RGS2
ENSSSCG00000011065	MASTL
ENSSSCG00000010896	ASPM
ENSSSCG00000010912	KIF14
ENSSSCG00000007366	MYBL2
ENSSSCG00000007493	AURKA
ENSSSCG00000007253	MAPRE1
ENSSSCG00000007235	TPX2
ENSSSCG00000007331	RBL1
ENSSSCG00000017645	TEX14
ENSSSCG00000017761	UNC119
ENSSSCG00000017758	SPAG5
ENSSSCG00000017471	CDC6
ENSSSCG00000017342	KIF18B
ENSSSCG00000028924	AURKB
ENSSSCG00000017178	SPHK1
ENSSSCG00000040486	BIRC5

ENSSSCG00000016522	PTN
ENSSSCG00000016658	ANLN
ENSSSCG00000028169	NCAPG2
ENSSSCG00000032315	XRCC2
ENSSSCG00000022823	MCM5
ENSSSCG00000034866	LOC106506226

Muscle contraction

Ensembl Gene ID	Gene Symbol
ENSSSCG00000005316	TPM2
ENSSSCG00000004570	TPM1
ENSSSCG00000038873	SLMAP
ENSSSCG00000058790	RYR1
ENSSSCG00000003218	MYBPC2
ENSSSCG00000027407	MYH14
ENSSSCG00000033260	TNNI2
ENSSSCG00000031903	TNNT3
ENSSSCG00000009856	NOS1
ENSSSCG00000010144	ACTN2
ENSSSCG00000015747	MYOM2
ENSSSCG00000007799	MYL11
ENSSSCG00000006631	TMOD4
ENSSSCG00000006307	RCSD1
ENSSSCG00000026498	STAC3; R3HDM2
ENSSSCG00000024784	CACNA1S
ENSSSCG00000031201	LMOD1
ENSSSCG00000007424	TNNC2
ENSSSCG00000017500	TCAP
ENSSSCG00000018005	MYH8
ENSSSCG00000016605	LMOD2
ENSSSCG00000056050	CLCN1
