# Supplementary material for: Dynamic changes in chromatin accessibility and gene expression involved in fetal myogenesis of Min pigs
Source: Anim Biosci. 2025 May 12;38(11):2525–36. doi: 10.5713/ab.25.0034 (PMC12580940; doi:10.5713/ab.25.0034)
Supplement: Supplementary file 7 [file ab-25-0034-supplementary-7.pdf]

Supplement 7. GO and KEGG enrichment analysis of clustering genes.

cluster4

Category	Term	PValue
GOTERM_BP_DIRECT	GO:0019882~antigen processing and presentation	1.57E-06
GOTERM_BP_DIRECT	GO:0016567~protein ubiquitination	9.64E-06
GOTERM_BP_DIRECT	GO:0019886~antigen processing and presentation of exogenous peptide antigen via MHC class II	4.19E-05
GOTERM_BP_DIRECT	GO:0006511~ubiquitin-dependent protein catabolic process	6.39E-05
GOTERM_BP_DIRECT	GO:0043161~proteasome-mediated ubiquitin-dependent protein catabolic process	1.11E-04
GOTERM_BP_DIRECT	GO:0003009~skeletal muscle contraction	1.49E-04
GOTERM_BP_DIRECT	GO:0007519~skeletal muscle tissue development	3.06E-04
GOTERM_BP_DIRECT	GO:0002503~peptide antigen assembly with MHC class II protein complex	8.35E-04
GOTERM_BP_DIRECT	GO:0010628~positive regulation of gene expression	0.001266603
GOTERM_BP_DIRECT	GO:0060291~long-term synaptic potentiation	0.001527337
GOTERM_BP_DIRECT	GO:0032469~endoplasmic reticulum calcium ion homeostasis	0.001627565
GOTERM_BP_DIRECT	GO:0000422~mitophagy	0.002057159
GOTERM_BP_DIRECT	GO:0002381~immunoglobulin production involved in immunoglobulin mediated immune response	0.0023073
GOTERM_BP_DIRECT	GO:0043484~regulation of RNA splicing	0.002656351
GOTERM_BP_DIRECT	GO:0045944~positive regulation of transcription from RNA polymerase II promoter	0.003039637
GOTERM_BP_DIRECT	GO:0050870~positive regulation of T cell activation	0.003144303
GOTERM_BP_DIRECT	GO:0043524~negative regulation of neuron apoptotic process	0.003616239
GOTERM_BP_DIRECT	GO:0000045~autophagosome assembly	0.003770943
GOTERM_BP_DIRECT	GO:0002504~antigen processing and presentation of peptide or polysaccharide antigen via MHC class II	0.00382972
GOTERM_BP_DIRECT	GO:0070373~negative regulation of ERK1 and ERK2 cascade	0.004196325
GOTERM_BP_DIRECT	GO:0060047~heart contraction	0.004248043
GOTERM_BP_DIRECT	GO:0051259~protein oligomerization	0.004248043
GOTERM_BP_DIRECT	GO:0045599~negative regulation of fat cell differentiation	0.00452326
GOTERM_BP_DIRECT	GO:0060136~embryonic process involved in female pregnancy	0.005184736

GOTERM_BP_DIRECT	GO:0007007~inner mitochondrial membrane organization	0.005184736
GOTERM_BP_DIRECT	GO:0016311~dephosphorylation	0.00600506
GOTERM_BP_DIRECT	GO:0045766~positive regulation of angiogenesis	0.006889967
GOTERM_BP_DIRECT	GO:0001843~neural tube closure	0.007053886
GOTERM_BP_DIRECT	GO:0070584~mitochondrion morphogenesis	0.00713552
GOTERM_BP_DIRECT	GO:0051603~proteolysis involved in cellular protein catabolic process	0.007137815
GOTERM_BP_DIRECT	GO:0071356~cellular response to tumor necrosis factor	0.007630329
GOTERM_BP_DIRECT	GO:0030213~hyaluronan biosynthetic process	0.008622004
GOTERM_BP_DIRECT	GO:0016079~synaptic vesicle exocytosis	0.008872851
GOTERM_BP_DIRECT	GO:0046716~muscle cell cellular homeostasis	0.008996607
GOTERM_BP_DIRECT	GO:0006470~protein dephosphorylation	0.009303597
GOTERM_BP_DIRECT	GO:0042127~regulation of cell proliferation	0.012834109
GOTERM_BP_DIRECT	GO:0097176~epoxide metabolic process	0.012922372
GOTERM_BP_DIRECT	GO:0005979~regulation of glycogen biosynthetic process	0.013111567
GOTERM_BP_DIRECT	GO:0032287~peripheral nervous system myelin maintenance	0.013111567
GOTERM_BP_DIRECT	GO:0009615~response to virus	0.013823755
GOTERM_BP_DIRECT	GO:0030155~regulation of cell adhesion	0.014041397
GOTERM_BP_DIRECT	GO:0030308~negative regulation of cell growth	0.01444292
GOTERM_BP_DIRECT	GO:0071280~cellular response to copper ion	0.015212299
GOTERM_BP_DIRECT	GO:0034599~cellular response to oxidative stress	0.016283276
GOTERM_BP_DIRECT	GO:0006816~calcium ion transport	0.017484653
GOTERM_BP_DIRECT	GO:0045921~positive regulation of exocytosis	0.019225685
GOTERM_BP_DIRECT	GO:0071456~cellular response to hypoxia	0.01995453
GOTERM_BP_DIRECT	GO:0032436~positive regulation of proteasomal ubiquitin-dependent protein catabolic process	0.021638275
GOTERM_BP_DIRECT	GO:0045732~positive regulation of protein catabolic process	0.021998619
GOTERM_BP_DIRECT	GO:0007265~Ras protein signal transduction	0.021998619
GOTERM_BP_DIRECT	GO:0044344~cellular response to fibroblast growth factor stimulus	0.023834189
GOTERM_BP_DIRECT	GO:0006986~response to unfolded protein	0.023834189
GOTERM_BP_DIRECT	GO:0006936~muscle contraction	0.024142165
GOTERM_BP_DIRECT	GO:0045786~negative regulation of cell cycle	0.024142165
GOTERM_BP_DIRECT	GO:1900106~positive regulation of hyaluranon cable assembly	0.024695758
GOTERM_BP_DIRECT	GO:0048102~autophagic cell death	0.024695758
GOTERM_BP_DIRECT	GO:0099105~ion channel modulating, G-protein coupled receptor signaling pathway	0.024695758

GOTERM_BP_DIRECT	GO:0098779~positive regulation of macromitophagy in response to mitochondrial depolarization	0.024695758
GOTERM_BP_DIRECT	GO:0061520~Langerhans cell differentiation	0.024695758
GOTERM_BP_DIRECT	GO:0071786~endoplasmic reticulum tubular network organization	0.025395288
GOTERM_BP_DIRECT	GO:0045050~protein insertion into ER membrane by stop-transfer membrane-anchor sequence	0.025395288
GOTERM_BP_DIRECT	GO:0002011~morphogenesis of an epithelial sheet	0.025395288
GOTERM_BP_DIRECT	GO:0050435~beta-amyloid metabolic process	0.025395288
GOTERM_BP_DIRECT	GO:0005975~carbohydrate metabolic process	0.025828426
GOTERM_BP_DIRECT	GO:0034341~response to interferon-gamma	0.027357635
GOTERM_BP_DIRECT	GO:0051881~regulation of mitochondrial membrane potential	0.027357635
GOTERM_BP_DIRECT	GO:0006914~autophagy	0.027959376
GOTERM_BP_DIRECT	GO:0030279~negative regulation of ossification	0.029055167
GOTERM_BP_DIRECT	GO:0007015~actin filament organization	0.030448992
GOTERM_BP_DIRECT	GO:0033627~cell adhesion mediated by integrin	0.031225808
GOTERM_BP_DIRECT	GO:0032728~positive regulation of interferon-beta production	0.031225808
GOTERM_BP_DIRECT	GO:0010595~positive regulation of endothelial cell migration	0.031225808
GOTERM_BP_DIRECT	GO:0033617~mitochondrial respiratory chain complex IV assembly	0.031775611
GOTERM_BP_DIRECT	GO:0050729~positive regulation of inflammatory response	0.032559732
GOTERM_BP_DIRECT	GO:0072659~protein localization to plasma membrane	0.033509641
GOTERM_BP_DIRECT	GO:0050778~positive regulation of immune response	0.034900406
GOTERM_BP_DIRECT	GO:0001541~ovarian follicle development	0.036614418
GOTERM_BP_DIRECT	GO:0010977~negative regulation of neuron projection development	0.036614418
GOTERM_BP_DIRECT	GO:1902514~regulation of calcium ion transmembrane transport via high voltage-gated calcium channel	0.039340549
GOTERM_BP_DIRECT	GO:0071281~cellular response to iron ion	0.039340549
GOTERM_BP_DIRECT	GO:0034641~cellular nitrogen compound metabolic process	0.039340549
GOTERM_BP_DIRECT	GO:0042552~myelination	0.039995165
GOTERM_BP_DIRECT	GO:0016310~phosphorylation	0.040070072
GOTERM_BP_DIRECT	GO:0060079~excitatory postsynaptic potential	0.041376356
GOTERM_BP_DIRECT	GO:0016236~macroautophagy	0.041881407
GOTERM_BP_DIRECT	GO:0045893~positive regulation of transcription, DNA-templated	0.047140833

GOTERM_BP_DIRECT	GO:0032543~mitochondrial translation	0.047581686
GOTERM_BP_DIRECT	GO:0070059~intrinsic apoptotic signaling pathway in response to endoplasmic reticulum stress	0.047581686
GOTERM_BP_DIRECT	GO:0045214~sarcomere organization	0.047581686
GOTERM_CC_DIRECT	GO:0005829~cytosol	1.77E-12
GOTERM_CC_DIRECT	GO:0005737~cytoplasm	1.53E-11
GOTERM_CC_DIRECT	GO:0005739~mitochondrion	5.57E-08
GOTERM_CC_DIRECT	GO:0030018~Z disc	1.16E-05
GOTERM_CC_DIRECT	GO:0005654~nucleoplasm	2.23E-05
GOTERM_CC_DIRECT	GO:0005634~nucleus	1.05E-04
GOTERM_CC_DIRECT	GO:0031902~late endosome membrane	1.53E-04
GOTERM_CC_DIRECT	GO:0005783~endoplasmic reticulum	2.02E-04
GOTERM_CC_DIRECT	GO:0016529~sarcoplasmic reticulum	3.18E-04
GOTERM_CC_DIRECT	GO:0042383~sarcolemma	3.33E-04
GOTERM_CC_DIRECT	GO:0048471~perinuclear region of cytoplasm	3.57E-04
GOTERM_CC_DIRECT	GO:0005604~basement membrane	8.87E-04
GOTERM_CC_DIRECT	GO:0042613~MHC class II protein complex	9.62E-04
GOTERM_CC_DIRECT	GO:0000421~autophagosome membrane	0.001247011
GOTERM_CC_DIRECT	GO:0001725~stress fiber	0.001550145
GOTERM_CC_DIRECT	GO:0043220~Schmidt-Lanterman incisure	0.001631251
GOTERM_CC_DIRECT	GO:0005762~mitochondrial large ribosomal subunit	0.001756512
GOTERM_CC_DIRECT	GO:0000164~protein phosphatase type 1 complex	0.003851721
GOTERM_CC_DIRECT	GO:0031410~cytoplasmic vesicle	0.004530338
GOTERM_CC_DIRECT	GO:0005741~mitochondrial outer membrane	0.005352939
GOTERM_CC_DIRECT	GO:0005777~peroxisome	0.007124197
GOTERM_CC_DIRECT	GO:0005789~endoplasmic reticulum membrane	0.008005575
GOTERM_CC_DIRECT	GO:0032991~macromolecular complex	0.008803491
GOTERM_CC_DIRECT	GO:0000502~proteasome complex	0.009366407
GOTERM_CC_DIRECT	GO:0005758~mitochondrial intermembrane space	0.009946747
GOTERM_CC_DIRECT	GO:0005759~mitochondrial matrix	0.010161585
GOTERM_CC_DIRECT	GO:0008305~integrin complex	0.010777461
GOTERM_CC_DIRECT	GO:0036117~hyaluranon cable	0.011792195
GOTERM_CC_DIRECT	GO:1990072~TRAPPIII protein complex	0.011792195
GOTERM_CC_DIRECT	GO:0005765~lysosomal membrane	0.012959176
GOTERM_CC_DIRECT	GO:0043209~myelin sheath	0.013703895
GOTERM_CC_DIRECT	GO:0071782~endoplasmic reticulum tubular network	0.016431106
GOTERM_CC_DIRECT	GO:0005761~mitochondrial ribosome	0.016479415
GOTERM_CC_DIRECT	GO:0031466~Cul5-RING ubiquitin ligase complex	0.016479415
GOTERM_CC_DIRECT	GO:0008021~synaptic vesicle	0.016660379
GOTERM_CC_DIRECT	GO:0005794~Golgi apparatus	0.020413245
GOTERM_CC_DIRECT	GO:0031594~neuromuscular junction	0.022265976
GOTERM_CC_DIRECT	GO:0000151~ubiquitin ligase complex	0.022265976

GOTERM_CC_DIRECT	GO:0045178~basal part of cell	0.022435752
GOTERM_CC_DIRECT	GO:0072546~ER membrane protein complex	0.022435752
GOTERM_CC_DIRECT	GO:0005743~mitochondrial inner membrane	0.02418905
GOTERM_CC_DIRECT	GO:0031966~mitochondrial membrane	0.024776236
GOTERM_CC_DIRECT	GO:0016607~nuclear speck	0.024813701
GOTERM_CC_DIRECT	GO:0005776~autophagosome	0.025550495
GOTERM_CC_DIRECT	GO:0014704~intercalated disc	0.035257562
GOTERM_CC_DIRECT	GO:1990712~HFE-transferrin receptor complex	0.036050885
GOTERM_CC_DIRECT	GO:0043025~neuronal cell body	0.039702724
GOTERM_CC_DIRECT	GO:0042645~mitochondrial nucleoid	0.040590968
GOTERM_CC_DIRECT	GO:0002080~acrosomal membrane	0.041934769
GOTERM_CC_DIRECT	GO:0055037~recycling endosome	0.042002735
GOTERM_CC_DIRECT	GO:0008540~proteasome regulatory particle, base subcomplex	0.046332328
GOTERM_CC_DIRECT	GO:0000407~pre-autophagosomal structure	0.048740451
GOTERM_MF_DIRECT	GO:0046872~metal ion binding	4.68E-07
GOTERM_MF_DIRECT	GO:0042802~identical protein binding	6.85E-07
GOTERM_MF_DIRECT	GO:0031624~ubiquitin conjugating enzyme binding	6.30E-05
GOTERM_MF_DIRECT	GO:0061630~ubiquitin protein ligase activity	2.98E-04
GOTERM_MF_DIRECT	GO:0008270~zinc ion binding	3.46E-04
GOTERM_MF_DIRECT	GO:0051019~mitogen-activated protein kinase binding	7.13E-04
GOTERM_MF_DIRECT	GO:0042605~peptide antigen binding	0.001261783
GOTERM_MF_DIRECT	GO:0023026~MHC class II protein complex binding	0.001330798
GOTERM_MF_DIRECT	GO:0031625~ubiquitin protein ligase binding	0.001506159
GOTERM_MF_DIRECT	GO:0004721~phosphoprotein phosphatase activity	0.002170137
GOTERM_MF_DIRECT	GO:0030552~cAMP binding	0.002785553
GOTERM_MF_DIRECT	GO:0004175~endopeptidase activity	0.003245092
GOTERM_MF_DIRECT	GO:0044183~protein binding involved in protein folding	0.003923814
GOTERM_MF_DIRECT	GO:0004596~peptide alpha-N-acetyltransferase activity	0.004097806
GOTERM_MF_DIRECT	GO:0004709~MAP kinase kinase kinase activity	0.006297077
GOTERM_MF_DIRECT	GO:0008157~protein phosphatase 1 binding	0.006297077
GOTERM_MF_DIRECT	GO:0043022~ribosome binding	0.006465442
GOTERM_MF_DIRECT	GO:0008233~peptidase activity	0.006912106
GOTERM_MF_DIRECT	GO:0061629~RNA polymerase II sequence-specific DNA binding transcription factor binding	0.00733498
GOTERM_MF_DIRECT	GO:0043130~ubiquitin binding	0.007875295
GOTERM_MF_DIRECT	GO:0036402~proteasome-activating ATPase activity	0.00793525
GOTERM_MF_DIRECT	GO:0042609~CD4 receptor binding	0.00793525

GOTERM_MF_DIRECT	GO:0004722~protein serine/threonine phosphatase activity	0.007937397
GOTERM_MF_DIRECT	GO:0004115~3',5'-cyclic-AMP phosphodiesterase activity	0.00799437
GOTERM_MF_DIRECT	GO:0019003~GDP binding	0.009029422
GOTERM_MF_DIRECT	GO:0043169~cation binding	0.010622341
GOTERM_MF_DIRECT	GO:0031489~myosin V binding	0.010622341
GOTERM_MF_DIRECT	GO:0032977~membrane insertase activity	0.010622341
GOTERM_MF_DIRECT	GO:0008092~cytoskeletal protein binding	0.011666093
GOTERM_MF_DIRECT	GO:0097371~MDM2/MDM4 family protein binding	0.012085383
GOTERM_MF_DIRECT	GO:0000210~NAD+ diphosphatase activity	0.012193809
GOTERM_MF_DIRECT	GO:1901612~cardiolipin binding	0.017258781
GOTERM_MF_DIRECT	GO:0016922~ligand-dependent nuclear receptor binding	0.017565621
GOTERM_MF_DIRECT	GO:0044325~ion channel binding	0.017630912
GOTERM_MF_DIRECT	GO:0008289~lipid binding	0.019601299
GOTERM_MF_DIRECT	GO:0015421~oligopeptide-transporting ATPase activity	0.023334985
GOTERM_MF_DIRECT	GO:0035529~NADH pyrophosphatase activity	0.023334985
GOTERM_MF_DIRECT	GO:0004301~epoxide hydrolase activity	0.023334985
GOTERM_MF_DIRECT	GO:0016740~transferase activity	0.023415092
GOTERM_MF_DIRECT	GO:0004879~RNA polymerase II transcription factor activity, ligand-activated sequence-specific DNA binding	0.025337056
GOTERM_MF_DIRECT	GO:0031434~mitogen-activated protein kinase kinase binding	0.030744747
GOTERM_MF_DIRECT	GO:0005516~calmodulin binding	0.031300029
GOTERM_MF_DIRECT	GO:0002020~protease binding	0.031966886
GOTERM_MF_DIRECT	GO:0051373~FATZ binding	0.037222573
GOTERM_MF_DIRECT	GO:0008318~protein prenyltransferase activity	0.037222573
GOTERM_MF_DIRECT	GO:0016798~hydrolase activity, acting on glycosyl bonds	0.037687307
GOTERM_MF_DIRECT	GO:0005227~calcium activated cation channel activity	0.039048921
GOTERM_MF_DIRECT	GO:0000978~RNA polymerase II core promoter proximal region sequence-specific DNA binding	0.040687389
GOTERM_MF_DIRECT	GO:0005178~integrin binding	0.041745077
GOTERM_MF_DIRECT	GO:0017018~myosin phosphatase activity	0.046410779
GOTERM_MF_DIRECT	GO:0016791~phosphatase activity	0.048308977
GOTERM_MF_DIRECT	GO:0000146~microfilament motor activity	0.048322189
GOTERM_MF_DIRECT	GO:0019911~structural constituent of myelin sheath	0.048365287
KEGG_PATHWAY	ssc05410:Hypertrophic cardiomyopathy	6.96E-06
KEGG_PATHWAY	ssc05145:Toxoplasmosis	7.98E-06

KEGG_PATHWAY	ssc04510:Focal adhesion	1.39E-05
KEGG_PATHWAY	ssc05169:Epstein-Barr virus infection	8.36E-05
KEGG_PATHWAY	ssc05412:Arrhythmogenic right ventricular cardiomyopathy	1.33E-04
KEGG_PATHWAY	ssc05211:Renal cell carcinoma	1.67E-04
KEGG_PATHWAY	ssc05414:Dilated cardiomyopathy	1.70E-04
KEGG_PATHWAY	ssc04140:Autophagy - animal	3.44E-04
KEGG_PATHWAY	ssc05152:Tuberculosis	3.44E-04
KEGG_PATHWAY	ssc04512:ECM-receptor interaction	3.53E-04
KEGG_PATHWAY	ssc05330:Allograft rejection	4.18E-04
KEGG_PATHWAY	ssc05168:Herpes simplex virus 1 infection	5.62E-04
KEGG_PATHWAY	ssc05310:Asthma	7.01E-04
KEGG_PATHWAY	ssc04514:Cell adhesion molecules	7.68E-04
KEGG_PATHWAY	ssc04910:Insulin signaling pathway	9.09E-04
KEGG_PATHWAY	ssc05332:Graft-versus-host disease	0.001029622
KEGG_PATHWAY	ssc04722:Neurotrophin signaling pathway	0.001159999
KEGG_PATHWAY	ssc05416:Viral myocarditis	0.001215036
KEGG_PATHWAY	ssc04137:Mitophagy - animal	0.001302325
KEGG_PATHWAY	ssc04068:FoxO signaling pathway	0.001304368
KEGG_PATHWAY	ssc04611:Platelet activation	0.001551863
KEGG_PATHWAY	ssc05222:Small cell lung cancer	0.001692523
KEGG_PATHWAY	ssc04672:Intestinal immune network for IgA production	0.001776762
KEGG_PATHWAY	ssc04010:MAPK signaling pathway	0.001853535
KEGG_PATHWAY	ssc04066:HIF-1 signaling pathway	0.002920102
KEGG_PATHWAY	ssc05165:Human papillomavirus infection	0.003197265
KEGG_PATHWAY	ssc04612:Antigen processing and presentation	0.003324574
KEGG_PATHWAY	ssc04922:Glucagon signaling pathway	0.003476751
KEGG_PATHWAY	ssc04929:GnRH secretion	0.003773002
KEGG_PATHWAY	ssc04720:Long-term potentiation	0.004268468
KEGG_PATHWAY	ssc04940:Type I diabetes mellitus	0.004696043
KEGG_PATHWAY	ssc05017:Spinocerebellar ataxia	0.004976352
KEGG_PATHWAY	ssc04020:Calcium signaling pathway	0.004984509
KEGG_PATHWAY	ssc04625:C-type lectin receptor signaling pathway	0.005051009
KEGG_PATHWAY	ssc05230:Central carbon metabolism in cancer	0.00541411
KEGG_PATHWAY	ssc05321:Inflammatory bowel disease	0.00541411
KEGG_PATHWAY	ssc04261:Adrenergic signaling in cardiomyocytes	0.006204652
KEGG_PATHWAY	ssc05320:Autoimmune thyroid disease	0.006240713
KEGG_PATHWAY	ssc04931:Insulin resistance	0.007783137
KEGG_PATHWAY	ssc04380:Osteoclast differentiation	0.007869469
KEGG_PATHWAY	ssc05140:Leishmaniasis	0.010348913
KEGG_PATHWAY	ssc04012:ErbB signaling pathway	0.011502501
KEGG_PATHWAY	ssc03050:Proteasome	0.011876862
KEGG_PATHWAY	ssc04810:Regulation of actin cytoskeleton	0.012905718

KEGG_PATHWAY	ssc04921:Oxytocin signaling pathway	0.013017598
KEGG_PATHWAY	ssc05010:Alzheimer disease	0.01336976
KEGG_PATHWAY	ssc04621:NOD-like receptor signaling pathway	0.016284007
KEGG_PATHWAY	ssc05210:Colorectal cancer	0.017735251
KEGG_PATHWAY	ssc04730:Long-term depression	0.018051556
KEGG_PATHWAY	ssc04933:AGE-RAGE signaling pathway in diabetic complications	0.018484062
KEGG_PATHWAY	ssc04213:Longevity regulating pathway - multiple species	0.019994246
KEGG_PATHWAY	ssc05214:Glioma	0.022033012
KEGG_PATHWAY	ssc04210:Apoptosis	0.023250117
KEGG_PATHWAY	ssc05200:Pathways in cancer	0.024004827
KEGG_PATHWAY	ssc01522:Endocrine resistance	0.02432036
KEGG_PATHWAY	ssc04371:Apelin signaling pathway	0.026266092
KEGG_PATHWAY	ssc04814:Motor proteins	0.029367569
KEGG_PATHWAY	ssc05164:Influenza A	0.029751835
KEGG_PATHWAY	ssc05231:Choline metabolism in cancer	0.030324272
KEGG_PATHWAY	ssc05220:Chronic myeloid leukemia	0.031033884
KEGG_PATHWAY	ssc04150:mTOR signaling pathway	0.032311429
KEGG_PATHWAY	ssc05142:Chagas disease	0.032622709
KEGG_PATHWAY	ssc04218:Cellular senescence	0.034110844
KEGG_PATHWAY	ssc04071:Sphingolipid signaling pathway	0.034470449
KEGG_PATHWAY	ssc04146:Peroxisome	0.034529963
KEGG_PATHWAY	ssc05146:Amoebiasis	0.037328853
KEGG_PATHWAY	ssc05208:Chemical carcinogenesis - reactive oxygen species	0.037552853
KEGG_PATHWAY	ssc00760:Nicotinate and nicotinamide metabolism	0.037991316
KEGG_PATHWAY	ssc04659:Th17 cell differentiation	0.039540919
KEGG_PATHWAY	ssc04650:Natural killer cell mediated cytotoxicity	0.039540919
KEGG_PATHWAY	ssc04662:B cell receptor signaling pathway	0.042358455
KEGG_PATHWAY	ssc05213:Endometrial cancer	0.042733927
KEGG_PATHWAY	ssc04912:GnRH signaling pathway	0.042738668
KEGG_PATHWAY	ssc05218:Melanoma	0.04467626
KEGG_PATHWAY	ssc04270:Vascular smooth muscle contraction	0.047458315
KEGG_PATHWAY	ssc04072:Phospholipase D signaling pathway	0.048282242
KEGG_PATHWAY	ssc05223:Non-small cell lung cancer	0.048299859

cluster2&7

Category	Term	PValue
GOTERM_BP_DIRECT	GO:0051301~cell division	8.42E-18
GOTERM_BP_DIRECT	GO:0006281~DNA repair	7.48E-15
GOTERM_BP_DIRECT	GO:0006260~DNA replication	2.04E-13
GOTERM_BP_DIRECT	GO:0007059~chromosome segregation	3.38E-13

GOTERM_BP_DIRECT	GO:0000724~double-strand break repair via homologous recombination	1.26E-12
GOTERM_BP_DIRECT	GO:0007049~cell cycle	1.02E-11
GOTERM_BP_DIRECT	GO:0006486~protein glycosylation	2.07E-10
GOTERM_BP_DIRECT	GO:0015031~protein transport	6.06E-10
GOTERM_BP_DIRECT	GO:0000398~mRNA splicing, via spliceosome	6.84E-10
GOTERM_BP_DIRECT	GO:0060271~cilium assembly	3.80E-09
GOTERM_BP_DIRECT	GO:0006270~DNA replication initiation	4.85E-09
GOTERM_BP_DIRECT	GO:0006886~intracellular protein transport	1.90E-08
GOTERM_BP_DIRECT	GO:0016310~phosphorylation	7.39E-08
GOTERM_BP_DIRECT	GO:0061512~protein localization to cilium	9.01E-08
GOTERM_BP_DIRECT	GO:0000278~mitotic cell cycle	1.08E-07
GOTERM_BP_DIRECT	GO:0000132~establishment of mitotic spindle orientation	1.45E-07
GOTERM_BP_DIRECT	GO:0007094~mitotic spindle assembly checkpoint	1.63E-07
GOTERM_BP_DIRECT	GO:0016192~vesicle-mediated transport	5.20E-07
GOTERM_BP_DIRECT	GO:0007098~centrosome cycle	5.55E-07
GOTERM_BP_DIRECT	GO:0036297~interstrand cross-link repair	1.49E-06
GOTERM_BP_DIRECT	GO:0006268~DNA unwinding involved in DNA replication	2.21E-06
GOTERM_BP_DIRECT	GO:0006334~nucleosome assembly	2.70E-06
GOTERM_BP_DIRECT	GO:1905168~positive regulation of double-strand break repair via homologous recombination	2.81E-06
GOTERM_BP_DIRECT	GO:0006338~chromatin remodeling	3.62E-06
GOTERM_BP_DIRECT	GO:0090307~mitotic spindle assembly	5.38E-06
GOTERM_BP_DIRECT	GO:0006325~chromatin organization	7.60E-06
GOTERM_BP_DIRECT	GO:0000070~mitotic sister chromatid segregation	1.06E-05
GOTERM_BP_DIRECT	GO:0007052~mitotic spindle organization	1.13E-05
GOTERM_BP_DIRECT	GO:0051726~regulation of cell cycle	1.41E-05
GOTERM_BP_DIRECT	GO:0051028~mRNA transport	1.49E-05
GOTERM_BP_DIRECT	GO:0007224~smoothened signaling pathway	2.05E-05
GOTERM_BP_DIRECT	GO:0006890~retrograde vesicle-mediated transport, Golgi to ER	2.23E-05
GOTERM_BP_DIRECT	GO:0000723~telomere maintenance	2.38E-05
GOTERM_BP_DIRECT	GO:0021987~cerebral cortex development	3.12E-05
GOTERM_BP_DIRECT	GO:0006406~mRNA export from nucleus	3.44E-05
GOTERM_BP_DIRECT	GO:0051315~attachment of mitotic spindle microtubules to kinetochore	3.86E-05
GOTERM_BP_DIRECT	GO:0000727~double-strand break repair via break-induced replication	3.86E-05
GOTERM_BP_DIRECT	GO:0031297~replication fork processing	4.26E-05
GOTERM_BP_DIRECT	GO:0006468~protein phosphorylation	4.65E-05
GOTERM_BP_DIRECT	GO:0030968~endoplasmic reticulum unfolded protein response	6.25E-05
GOTERM_BP_DIRECT	GO:0017148~negative regulation of translation	7.35E-05

GOTERM_BP_DIRECT	GO:0000281~mitotic cytokinesis	8.62E-05
GOTERM_BP_DIRECT	GO:0006606~protein import into nucleus	1.38E-04
GOTERM_BP_DIRECT	GO:0010971~positive regulation of G2/M transition of mitotic cell cycle	2.04E-04
GOTERM_BP_DIRECT	GO:0006457~protein folding	3.11E-04
GOTERM_BP_DIRECT	GO:0032212~positive regulation of telomere maintenance via telomerase	3.19E-04
GOTERM_BP_DIRECT	GO:0030030~cell projection organization	3.19E-04
GOTERM_BP_DIRECT	GO:0006261~DNA-dependent DNA replication	3.36E-04
GOTERM_BP_DIRECT	GO:0006888~ER to Golgi vesicle-mediated transport	3.72E-04
GOTERM_BP_DIRECT	GO:0010212~response to ionizing radiation	4.45E-04
GOTERM_BP_DIRECT	GO:0000122~negative regulation of transcription from RNA polymerase II promoter	4.54E-04
GOTERM_BP_DIRECT	GO:0006284~base-excision repair	4.94E-04
GOTERM_BP_DIRECT	GO:0001654~eye development	4.94E-04
GOTERM_BP_DIRECT	GO:0007051~spindle organization	5.06E-04
GOTERM_BP_DIRECT	GO:0006897~endocytosis	5.52E-04
GOTERM_BP_DIRECT	GO:0018279~protein N-linked glycosylation via asparagine	5.68E-04
GOTERM_BP_DIRECT	GO:0000387~spliceosomal snRNP assembly	5.71E-04
GOTERM_BP_DIRECT	GO:0000086~G2/M transition of mitotic cell cycle	5.76E-04
GOTERM_BP_DIRECT	GO:0006513~protein monoubiquitination	6.01E-04
GOTERM_BP_DIRECT	GO:0006364~rRNA processing	6.34E-04
GOTERM_BP_DIRECT	GO:1905515~non-motile cilium assembly	6.57E-04
GOTERM_BP_DIRECT	GO:0007095~mitotic G2 DNA damage checkpoint	6.59E-04
GOTERM_BP_DIRECT	GO:0008380~RNA splicing	7.45E-04
GOTERM_BP_DIRECT	GO:0006974~cellular response to DNA damage stimulus	7.93E-04
GOTERM_BP_DIRECT	GO:0048812~neuron projection morphogenesis	8.22E-04
GOTERM_BP_DIRECT	GO:0006493~protein O-linked glycosylation	8.27E-04
GOTERM_BP_DIRECT	GO:0007163~establishment or maintenance of cell polarity	8.32E-04
GOTERM_BP_DIRECT	GO:0072583~clathrin-dependent endocytosis	8.32E-04
GOTERM_BP_DIRECT	GO:0034080~CENP-A containing nucleosome assembly	8.97E-04
GOTERM_BP_DIRECT	GO:0007080~mitotic metaphase plate congression	9.53E-04
GOTERM_BP_DIRECT	GO:0060236~regulation of mitotic spindle organization	9.62E-04
GOTERM_BP_DIRECT	GO:0021915~neural tube development	9.71E-04
GOTERM_BP_DIRECT	GO:0001701~in utero embryonic development	9.80E-04
GOTERM_BP_DIRECT	GO:0001578~microtubule bundle formation	0.001402375
GOTERM_BP_DIRECT	GO:0008284~positive regulation of cell proliferation	0.001596759
GOTERM_BP_DIRECT	GO:0035721~intraciliary retrograde transport	0.001633159
GOTERM_BP_DIRECT	GO:0007076~mitotic chromosome condensation	0.001633159
GOTERM_BP_DIRECT	GO:0010498~proteasomal protein catabolic process	0.001825457
GOTERM_BP_DIRECT	GO:0016477~cell migration	0.001856638
GOTERM_BP_DIRECT	GO:0007409~axonogenesis	0.002018599

GOTERM_BP_DIRECT	GO:0010634~positive regulation of epithelial cell migration	0.002093741
GOTERM_BP_DIRECT	GO:0000462~maturation of SSU-rRNA from tricistronic rRNA transcript (SSU-rRNA, 5.8S rRNA, LSU-rRNA)	0.002093741
GOTERM_BP_DIRECT	GO:0007420~brain development	0.002200946
GOTERM_BP_DIRECT	GO:0070934~CRD-mediated mRNA stabilization	0.002258508
GOTERM_BP_DIRECT	GO:0006098~pentose-phosphate shunt	0.002258508
GOTERM_BP_DIRECT	GO:0000226~microtubule cytoskeleton organization	0.002273217
GOTERM_BP_DIRECT	GO:0006891~intra-Golgi vesicle-mediated transport	0.002369304
GOTERM_BP_DIRECT	GO:0007264~small GTPase mediated signal transduction	0.002516299
GOTERM_BP_DIRECT	GO:0034644~cellular response to UV	0.002568692
GOTERM_BP_DIRECT	GO:0090263~positive regulation of canonical Wnt signaling pathway	0.002572102
GOTERM_BP_DIRECT	GO:0006139~nucleobase-containing compound metabolic process	0.002836338
GOTERM_BP_DIRECT	GO:0043687~post-translational protein modification	0.002836338
GOTERM_BP_DIRECT	GO:0030036~actin cytoskeleton organization	0.002846298
GOTERM_BP_DIRECT	GO:0006310~DNA recombination	0.002880482
GOTERM_BP_DIRECT	GO:0032211~negative regulation of telomere maintenance via telomerase	0.002921907
GOTERM_BP_DIRECT	GO:0001843~neural tube closure	0.002981863
GOTERM_BP_DIRECT	GO:0042147~retrograde transport, endosome to Golgi	0.003106186
GOTERM_BP_DIRECT	GO:0007346~regulation of mitotic cell cycle	0.0034544
GOTERM_BP_DIRECT	GO:0031398~positive regulation of protein ubiquitination	0.003627797
GOTERM_BP_DIRECT	GO:0043065~positive regulation of apoptotic process	0.003633794
GOTERM_BP_DIRECT	GO:0032465~regulation of cytokinesis	0.0037827
GOTERM_BP_DIRECT	GO:0099560~synaptic membrane adhesion	0.004012331
GOTERM_BP_DIRECT	GO:0044772~mitotic cell cycle phase transition	0.004012331
GOTERM_BP_DIRECT	GO:0070979~protein K11-linked ubiquitination	0.004304936
GOTERM_BP_DIRECT	GO:0006611~protein export from nucleus	0.004304936
GOTERM_BP_DIRECT	GO:0006298~mismatch repair	0.004492607
GOTERM_BP_DIRECT	GO:0048025~negative regulation of mRNA splicing, via spliceosome	0.004492607
GOTERM_BP_DIRECT	GO:0006275~regulation of DNA replication	0.004575391
GOTERM_BP_DIRECT	GO:0006302~double-strand break repair	0.004602255
GOTERM_BP_DIRECT	GO:0006024~glycosaminoglycan biosynthetic process	0.00474366
GOTERM_BP_DIRECT	GO:1901838~positive regulation of transcription of nuclear large rRNA transcript from RNA polymerase I promoter	0.00474366
GOTERM_BP_DIRECT	GO:2000036~regulation of stem cell population maintenance	0.00474366
GOTERM_BP_DIRECT	GO:0042770~signal transduction in response to DNA damage	0.00474366
GOTERM_BP_DIRECT	GO:0014032~neural crest cell development	0.004882044

GOTERM_BP_DIRECT	GO:1900026~positive regulation of substrate adhesion-dependent cell spreading	0.004978255
GOTERM_BP_DIRECT	GO:0033962~cytoplasmic mRNA processing body assembly	0.005045018
GOTERM_BP_DIRECT	GO:0030199~collagen fibril organization	0.005183825
GOTERM_BP_DIRECT	GO:0060070~canonical Wnt signaling pathway	0.005186861
GOTERM_BP_DIRECT	GO:1905832~positive regulation of spindle assembly	0.005419031
GOTERM_BP_DIRECT	GO:0006335~DNA replication-dependent nucleosome assembly	0.005419031
GOTERM_BP_DIRECT	GO:1902975~mitotic DNA replication initiation	0.005419031
GOTERM_BP_DIRECT	GO:0060831~smoothened signaling pathway involved in dorsal/ventral neural tube patterning	0.005419031
GOTERM_BP_DIRECT	GO:0000209~protein polyubiquitination	0.005597438
GOTERM_BP_DIRECT	GO:0035556~intracellular signal transduction	0.005632203
GOTERM_BP_DIRECT	GO:0030433~ubiquitin-dependent ERAD pathway	0.005657801
GOTERM_BP_DIRECT	GO:0035307~positive regulation of protein dephosphorylation	0.005910086
GOTERM_BP_DIRECT	GO:2000042~negative regulation of double-strand break repair via homologous recombination	0.005910086
GOTERM_BP_DIRECT	GO:0060976~coronary vasculature development	0.005910086
GOTERM_BP_DIRECT	GO:0042073~intraciliary transport	0.005910086
GOTERM_BP_DIRECT	GO:0002062~chondrocyte differentiation	0.006314548
GOTERM_BP_DIRECT	GO:0009887~animal organ morphogenesis	0.006780434
GOTERM_BP_DIRECT	GO:0006541~glutamine metabolic process	0.00681329
GOTERM_BP_DIRECT	GO:0045879~negative regulation of smoothened signaling pathway	0.007107451
GOTERM_BP_DIRECT	GO:0000079~regulation of cyclin-dependent protein serine/threonine kinase activity	0.007107451
GOTERM_BP_DIRECT	GO:0043923~positive regulation by host of viral transcription	0.007708719
GOTERM_BP_DIRECT	GO:0050821~protein stabilization	0.007787415
GOTERM_BP_DIRECT	GO:0006695~cholesterol biosynthetic process	0.008075246
GOTERM_BP_DIRECT	GO:0044027~hypermethylation of CpG island	0.008104997
GOTERM_BP_DIRECT	GO:0006177~GMP biosynthetic process	0.008104997
GOTERM_BP_DIRECT	GO:0042733~embryonic digit morphogenesis	0.008227925
GOTERM_BP_DIRECT	GO:0045184~establishment of protein localization	0.008439217
GOTERM_BP_DIRECT	GO:0000463~maturation of LSU-rRNA from tricistronic rRNA transcript (SSU-rRNA, 5.8S rRNA, LSU-rRNA)	0.008456877
GOTERM_BP_DIRECT	GO:0006337~nucleosome disassembly	0.008456877
GOTERM_BP_DIRECT	GO:0007019~microtubule depolymerization	0.008456877
GOTERM_BP_DIRECT	GO:0090160~Golgi to lysosome transport	0.008775194
GOTERM_BP_DIRECT	GO:0016180~snRNA processing	0.008775194
GOTERM_BP_DIRECT	GO:0043010~camera-type eye development	0.008829037

GOTERM_BP_DIRECT	GO:0042307~positive regulation of protein import into nucleus	0.008829037
GOTERM_BP_DIRECT	GO:0007018~microtubule-based movement	0.008914098
GOTERM_BP_DIRECT	GO:0007030~Golgi organization	0.00937348
GOTERM_BP_DIRECT	GO:0032331~negative regulation of chondrocyte differentiation	0.009950788
GOTERM_BP_DIRECT	GO:0009966~regulation of signal transduction	0.010061235
GOTERM_BP_DIRECT	GO:0007157~heterophilic cell-cell adhesion via plasma membrane cell adhesion molecules	0.010061235
GOTERM_BP_DIRECT	GO:0032456~endocytic recycling	0.010212473
GOTERM_BP_DIRECT	GO:0000381~regulation of alternative mRNA splicing, via spliceosome	0.010212473
GOTERM_BP_DIRECT	GO:0048144~fibroblast proliferation	0.010737589
GOTERM_BP_DIRECT	GO:0045880~positive regulation of smoothened signaling pathway	0.010737589
GOTERM_BP_DIRECT	GO:0007032~endosome organization	0.01147356
GOTERM_BP_DIRECT	GO:0006913~nucleocytoplasmic transport	0.011606623
GOTERM_BP_DIRECT	GO:0045724~positive regulation of cilium assembly	0.011606623
GOTERM_BP_DIRECT	GO:0021591~ventricular system development	0.011606623
GOTERM_BP_DIRECT	GO:0010569~regulation of double-strand break repair via homologous recombination	0.011606623
GOTERM_BP_DIRECT	GO:0001756~somitogenesis	0.011988125
GOTERM_BP_DIRECT	GO:0006487~protein N-linked glycosylation	0.0123192
GOTERM_BP_DIRECT	GO:0002052~positive regulation of neuroblast proliferation	0.013060106
GOTERM_BP_DIRECT	GO:0000184~nuclear-transcribed mRNA catabolic process, nonsense-mediated decay	0.013060106
GOTERM_BP_DIRECT	GO:0034501~protein localization to kinetochore	0.013299977
GOTERM_BP_DIRECT	GO:0000056~ribosomal small subunit export from nucleus	0.013818163
GOTERM_BP_DIRECT	GO:0043504~mitochondrial DNA repair	0.013818163
GOTERM_BP_DIRECT	GO:0014029~neural crest formation	0.013818163
GOTERM_BP_DIRECT	GO:0097294~'de novo' XMP biosynthetic process	0.013818163
GOTERM_BP_DIRECT	GO:0010833~telomere maintenance via telomere lengthening	0.013818163
GOTERM_BP_DIRECT	GO:0006269~DNA replication, synthesis of RNA primer	0.013818163
GOTERM_BP_DIRECT	GO:0016266~O-glycan processing	0.013818163
GOTERM_BP_DIRECT	GO:0051256~mitotic spindle midzone assembly	0.013818163
GOTERM_BP_DIRECT	GO:0001837~epithelial to mesenchymal transition	0.014028636
GOTERM_BP_DIRECT	GO:0048026~positive regulation of mRNA splicing, via spliceosome	0.014062028
GOTERM_BP_DIRECT	GO:0051402~neuron apoptotic process	0.014586759
GOTERM_BP_DIRECT	GO:0045995~regulation of embryonic development	0.014691554
GOTERM_BP_DIRECT	GO:0043087~regulation of GTPase activity	0.014691554

GOTERM_BP_DIRECT	GO:0030833~regulation of actin filament polymerization	0.014691554
GOTERM_BP_DIRECT	GO:0006164~purine nucleotide biosynthetic process	0.014770255
GOTERM_BP_DIRECT	GO:0032874~positive regulation of stress-activated MAPK cascade	0.014770255
GOTERM_BP_DIRECT	GO:0045665~negative regulation of neuron differentiation	0.015321525
GOTERM_BP_DIRECT	GO:0071318~cellular response to ATP	0.015413107
GOTERM_BP_DIRECT	GO:0010389~regulation of G2/M transition of mitotic cell cycle	0.015413107
GOTERM_BP_DIRECT	GO:0051492~regulation of stress fiber assembly	0.015413107
GOTERM_BP_DIRECT	GO:0000380~alternative mRNA splicing, via spliceosome	0.016780453
GOTERM_BP_DIRECT	GO:0016601~Rac protein signal transduction	0.016780453
GOTERM_BP_DIRECT	GO:0007411~axon guidance	0.016812926
GOTERM_BP_DIRECT	GO:0045739~positive regulation of DNA repair	0.017171896
GOTERM_BP_DIRECT	GO:0090090~negative regulation of canonical Wnt signaling pathway	0.017283633
GOTERM_BP_DIRECT	GO:0045815~positive regulation of gene expression, epigenetic	0.018033241
GOTERM_BP_DIRECT	GO:0048488~synaptic vesicle endocytosis	0.018033241
GOTERM_BP_DIRECT	GO:0048568~embryonic organ development	0.019302562
GOTERM_BP_DIRECT	GO:0007616~long-term memory	0.019302562
GOTERM_BP_DIRECT	GO:0050804~modulation of synaptic transmission	0.019719255
GOTERM_BP_DIRECT	GO:0007088~regulation of mitotic nuclear division	0.019847257
GOTERM_BP_DIRECT	GO:0060382~regulation of DNA strand elongation	0.019847257
GOTERM_BP_DIRECT	GO:1990403~embryonic brain development	0.019847257
GOTERM_BP_DIRECT	GO:0008608~attachment of spindle microtubules to kinetochore	0.019847257
GOTERM_BP_DIRECT	GO:0071479~cellular response to ionizing radiation	0.022835435
GOTERM_BP_DIRECT	GO:0032963~collagen metabolic process	0.023106172
GOTERM_BP_DIRECT	GO:0032897~negative regulation of viral transcription	0.023106172
GOTERM_BP_DIRECT	GO:0051603~proteolysis involved in cellular protein catabolic process	0.023162017
GOTERM_BP_DIRECT	GO:2000780~negative regulation of double-strand break repair	0.023196409
GOTERM_BP_DIRECT	GO:0090235~regulation of metaphase plate congression	0.023196409
GOTERM_BP_DIRECT	GO:0046726~positive regulation by virus of viral protein levels in host cell	0.023196409
GOTERM_BP_DIRECT	GO:0006397~mRNA processing	0.023247093
GOTERM_BP_DIRECT	GO:0071539~protein localization to centrosome	0.023425229
GOTERM_BP_DIRECT	GO:0035567~non-canonical Wnt signaling pathway	0.023425229
GOTERM_BP_DIRECT	GO:0030335~positive regulation of cell migration	0.024993888
GOTERM_BP_DIRECT	GO:0001822~kidney development	0.025599356
GOTERM_BP_DIRECT	GO:0001933~negative regulation of protein phosphorylation	0.025606243
GOTERM_BP_DIRECT	GO:0007099~centriole replication	0.025820569

GOTERM_BP_DIRECT	GO:0034063~stress granule assembly	0.025820569
GOTERM_BP_DIRECT	GO:1904851~positive regulation of establishment of protein localization to telomere	0.026082554
GOTERM_BP_DIRECT	GO:0016446~somatic hypermutation of immunoglobulin genes	0.026082554
GOTERM_BP_DIRECT	GO:0060563~neuroepithelial cell differentiation	0.026082554
GOTERM_BP_DIRECT	GO:0031076~embryonic camera-type eye development	0.026082554
GOTERM_BP_DIRECT	GO:0018242~protein O-linked glycosylation via serine	0.026082554
GOTERM_BP_DIRECT	GO:0016567~protein ubiquitination	0.026367414
GOTERM_BP_DIRECT	GO:0001947~heart looping	0.027123011
GOTERM_BP_DIRECT	GO:0071526~semaphorin-plexin signaling pathway	0.027324718
GOTERM_BP_DIRECT	GO:1900152~negative regulation of nuclear-transcribed mRNA catabolic process, deadenylation-dependent decay	0.027442532
GOTERM_BP_DIRECT	GO:0006996~organelle organization	0.027442532
GOTERM_BP_DIRECT	GO:0030948~negative regulation of vascular endothelial growth factor receptor signaling pathway	0.027442532
GOTERM_BP_DIRECT	GO:0010032~meiotic chromosome condensation	0.027442532
GOTERM_BP_DIRECT	GO:0097435~supramolecular fiber organization	0.027442532
GOTERM_BP_DIRECT	GO:0051988~regulation of attachment of spindle microtubules to kinetochore	0.027442532
GOTERM_BP_DIRECT	GO:0000076~DNA replication checkpoint	0.027442532
GOTERM_BP_DIRECT	GO:0098974~postsynaptic actin cytoskeleton organization	0.027442532
GOTERM_BP_DIRECT	GO:0006418~tRNA aminoacylation for protein translation	0.027442532
GOTERM_BP_DIRECT	GO:0008542~visual learning	0.028166106
GOTERM_BP_DIRECT	GO:0022008~neurogenesis	0.028221998
GOTERM_BP_DIRECT	GO:0015012~heparan sulfate proteoglycan biosynthetic process	0.028344234
GOTERM_BP_DIRECT	GO:0099072~regulation of postsynaptic specialization membrane neurotransmitter receptor levels	0.028344234
GOTERM_BP_DIRECT	GO:0031145~anaphase-promoting complex-dependent catabolic process	0.028344234
GOTERM_BP_DIRECT	GO:0060294~cilium movement involved in cell motility	0.028344234
GOTERM_BP_DIRECT	GO:0006605~protein targeting	0.028344234
GOTERM_BP_DIRECT	GO:0045944~positive regulation of transcription from RNA polymerase II promoter	0.028451431
GOTERM_BP_DIRECT	GO:0043488~regulation of mRNA stability	0.031717192
GOTERM_BP_DIRECT	GO:0031647~regulation of protein stability	0.032232276
GOTERM_BP_DIRECT	GO:0009954~proximal/distal pattern formation	0.033751121
GOTERM_BP_DIRECT	GO:0030511~positive regulation of transforming growth factor beta receptor signaling pathway	0.033751121
GOTERM_BP_DIRECT	GO:0045197~establishment or maintenance of epithelial cell apical/basal polarity	0.033751121
GOTERM_BP_DIRECT	GO:0050772~positive regulation of axonogenesis	0.033751121

GOTERM_BP_DIRECT	GO:0006383~transcription from RNA polymerase III promoter	0.034093142
GOTERM_BP_DIRECT	GO:0070085~glycosylation	0.034093142
GOTERM_BP_DIRECT	GO:0007097~nuclear migration	0.034093142
GOTERM_BP_DIRECT	GO:0001833~inner cell mass cell proliferation	0.034093142
GOTERM_BP_DIRECT	GO:0048013~ephrin receptor signaling pathway	0.034832409
GOTERM_BP_DIRECT	GO:0031333~negative regulation of protein complex assembly	0.034832409
GOTERM_BP_DIRECT	GO:0051017~actin filament bundle assembly	0.034832409
GOTERM_BP_DIRECT	GO:0016925~protein sumoylation	0.035152285
GOTERM_BP_DIRECT	GO:0035264~multicellular organism growth	0.036615795
GOTERM_BP_DIRECT	GO:0007064~mitotic sister chromatid cohesion	0.038995442
GOTERM_BP_DIRECT	GO:0045765~regulation of angiogenesis	0.038995442
GOTERM_BP_DIRECT	GO:0006491~N-glycan processing	0.038995442
GOTERM_BP_DIRECT	GO:0090043~regulation of tubulin deacetylation	0.040505355
GOTERM_BP_DIRECT	GO:0006241~CTP biosynthetic process	0.040505355
GOTERM_BP_DIRECT	GO:0000956~nuclear-transcribed mRNA catabolic process	0.040505355
GOTERM_BP_DIRECT	GO:0098887~neurotransmitter receptor transport, endosome to postsynaptic membrane	0.040505355
GOTERM_BP_DIRECT	GO:0071353~cellular response to interleukin-4	0.041805858
GOTERM_BP_DIRECT	GO:0001935~endothelial cell proliferation	0.041805858
GOTERM_BP_DIRECT	GO:0090398~cellular senescence	0.043662665
GOTERM_BP_DIRECT	GO:0051085~chaperone mediated protein folding requiring cofactor	0.043662665
GOTERM_BP_DIRECT	GO:0000902~cell morphogenesis	0.044366599
GOTERM_BP_DIRECT	GO:0007399~nervous system development	0.045346198
GOTERM_BP_DIRECT	GO:0070534~protein K63-linked ubiquitination	0.04637654
GOTERM_BP_DIRECT	GO:1904491~protein localization to ciliary transition zone	0.046780067
GOTERM_BP_DIRECT	GO:0019896~axonal transport of mitochondrion	0.046780067
GOTERM_BP_DIRECT	GO:0009134~nucleoside diphosphate catabolic process	0.046780067
GOTERM_BP_DIRECT	GO:0033235~positive regulation of protein sumoylation	0.046780067
GOTERM_BP_DIRECT	GO:0036295~cellular response to increased oxygen levels	0.046780067
GOTERM_BP_DIRECT	GO:0090267~positive regulation of mitotic cell cycle spindle assembly checkpoint	0.046780067
GOTERM_BP_DIRECT	GO:0098712~L-glutamate import across plasma membrane	0.046780067
GOTERM_BP_DIRECT	GO:0030953~astral microtubule organization	0.046780067
GOTERM_BP_DIRECT	GO:1990918~double-strand break repair involved in meiotic recombination	0.046780067
GOTERM_BP_DIRECT	GO:0071481~cellular response to X-ray	0.046780067
GOTERM_BP_DIRECT	GO:0031053~primary miRNA processing	0.046780067
GOTERM_BP_DIRECT	GO:0032147~activation of protein kinase activity	0.046780067

GOTERM_BP_DIRECT	GO:1905820~positive regulation of chromosome separation	0.046780067
GOTERM_BP_DIRECT	GO:0030336~negative regulation of cell migration	0.04685477
GOTERM_BP_DIRECT	GO:0051302~regulation of cell division	0.047955253
GOTERM_BP_DIRECT	GO:0090110~cargo loading into COPII-coated vesicle	0.047955253
GOTERM_BP_DIRECT	GO:0051220~cytoplasmic sequestering of protein	0.049862476
GOTERM_BP_DIRECT	GO:1904778~positive regulation of protein localization to cell cortex	0.049862476
GOTERM_BP_DIRECT	GO:0006271~DNA strand elongation involved in DNA replication	0.049862476
GOTERM_BP_DIRECT	GO:0098968~neurotransmitter receptor transport postsynaptic membrane to endosome	0.049862476
GOTERM_BP_DIRECT	GO:1903566~positive regulation of protein localization to cilium	0.049862476
GOTERM_BP_DIRECT	GO:0046602~regulation of mitotic centrosome separation	0.049862476
GOTERM_BP_DIRECT	GO:0032388~positive regulation of intracellular transport	0.049862476
GOTERM_BP_DIRECT	GO:0021696~cerebellar cortex morphogenesis	0.049862476
GOTERM_BP_DIRECT	GO:0044727~DNA demethylation of male pronucleus	0.049862476
GOTERM_BP_DIRECT	GO:0010826~negative regulation of centrosome duplication	0.049862476
GOTERM_BP_DIRECT	GO:0051664~nuclear pore localization	0.049862476
GOTERM_BP_DIRECT	GO:0071459~protein localization to chromosome, centromeric region	0.049862476
GOTERM_BP_DIRECT	GO:0030071~regulation of mitotic metaphase/anaphase transition	0.049862476
GOTERM_BP_DIRECT	GO:0048254~snoRNA localization	0.049862476
GOTERM_BP_DIRECT	GO:0045589~regulation of regulatory T cell differentiation	0.049862476
GOTERM_BP_DIRECT	GO:0072006~nephron development	0.049862476
GOTERM_CC_DIRECT	GO:0005654~nucleoplasm	3.94E-60
GOTERM_CC_DIRECT	GO:0005829~cytosol	6.95E-46
GOTERM_CC_DIRECT	GO:0005634~nucleus	6.55E-44
GOTERM_CC_DIRECT	GO:0005737~cytoplasm	2.79E-37
GOTERM_CC_DIRECT	GO:0005730~nucleolus	3.33E-24
GOTERM_CC_DIRECT	GO:0005813~centrosome	4.32E-23
GOTERM_CC_DIRECT	GO:0000776~kinetochore	7.34E-15
GOTERM_CC_DIRECT	GO:0005783~endoplasmic reticulum	2.01E-14
GOTERM_CC_DIRECT	GO:0005794~Golgi apparatus	6.33E-14
GOTERM_CC_DIRECT	GO:0000139~Golgi membrane	2.01E-12
GOTERM_CC_DIRECT	GO:0000781~chromosome, telomeric region	2.67E-12
GOTERM_CC_DIRECT	GO:0072686~mitotic spindle	6.25E-12
GOTERM_CC_DIRECT	GO:0000785~chromatin	2.12E-11
GOTERM_CC_DIRECT	GO:0016607~nuclear speck	7.29E-11
GOTERM_CC_DIRECT	GO:0030496~midbody	2.00E-10

GOTERM_CC_DIRECT	GO:0031965~nuclear membrane	5.32E-10
GOTERM_CC_DIRECT	GO:0005814~centriole	5.67E-10
GOTERM_CC_DIRECT	GO:0036064~ciliary basal body	6.23E-10
GOTERM_CC_DIRECT	GO:0000922~spindle pole	3.27E-09
GOTERM_CC_DIRECT	GO:0005694~chromosome	4.57E-09
GOTERM_CC_DIRECT	GO:0071013~catalytic step 2 spliceosome	6.89E-09
GOTERM_CC_DIRECT	GO:0045171~intercellular bridge	1.24E-08
GOTERM_CC_DIRECT	GO:0048471~perinuclear region of cytoplasm	1.90E-08
GOTERM_CC_DIRECT	GO:0005874~microtubule	2.35E-08
GOTERM_CC_DIRECT	GO:0071005~U2-type precatalytic spliceosome	5.44E-08
GOTERM_CC_DIRECT	GO:0071162~CMG complex	3.34E-07
GOTERM_CC_DIRECT	GO:0001650~fibrillar center	1.55E-06
GOTERM_CC_DIRECT	GO:0000940~condensed chromosome outer kinetochore	1.69E-06
GOTERM_CC_DIRECT	GO:0005681~spliceosomal complex	1.73E-06
GOTERM_CC_DIRECT	GO:0035371~microtubule plus-end	2.81E-06
GOTERM_CC_DIRECT	GO:0005929~cilium	3.13E-06
GOTERM_CC_DIRECT	GO:0035861~site of double-strand break	4.16E-06
GOTERM_CC_DIRECT	GO:0000775~chromosome, centromeric region	4.24E-06
GOTERM_CC_DIRECT	GO:0016581~NuRD complex	4.34E-06
GOTERM_CC_DIRECT	GO:0000932~P-body	5.72E-06
GOTERM_CC_DIRECT	GO:0005689~U12-type spliceosomal complex	5.99E-06
GOTERM_CC_DIRECT	GO:0005686~U2 snRNP	5.99E-06
GOTERM_CC_DIRECT	GO:0005789~endoplasmic reticulum membrane	6.07E-06
GOTERM_CC_DIRECT	GO:0005721~pericentric heterochromatin	7.00E-06
GOTERM_CC_DIRECT	GO:0016363~nuclear matrix	8.56E-06
GOTERM_CC_DIRECT	GO:0046540~U4/U6 x U5 tri-snRNP complex	1.19E-05
GOTERM_CC_DIRECT	GO:0005788~endoplasmic reticulum lumen	1.94E-05
GOTERM_CC_DIRECT	GO:0005876~spindle microtubule	3.27E-05
GOTERM_CC_DIRECT	GO:0031390~Ctf18 RFC-like complex	4.18E-05
GOTERM_CC_DIRECT	GO:0015629~actin cytoskeleton	4.51E-05
GOTERM_CC_DIRECT	GO:0016604~nuclear body	6.29E-05
GOTERM_CC_DIRECT	GO:0005819~spindle	6.49E-05
GOTERM_CC_DIRECT	GO:0051233~spindle midzone	7.10E-05
GOTERM_CC_DIRECT	GO:0030117~membrane coat	7.15E-05
GOTERM_CC_DIRECT	GO:0098978~glutamatergic synapse	1.00E-04
GOTERM_CC_DIRECT	GO:0042555~MCM complex	1.09E-04
GOTERM_CC_DIRECT	GO:0036464~cytoplasmic ribonucleoprotein granule	1.36E-04
GOTERM_CC_DIRECT	GO:0005635~nuclear envelope	1.71E-04
GOTERM_CC_DIRECT	GO:0034451~centriolar satellite	1.81E-04
GOTERM_CC_DIRECT	GO:0071007~U2-type catalytic step 2 spliceosome	1.91E-04
GOTERM_CC_DIRECT	GO:0000939~condensed chromosome inner kinetochore	2.03E-04
GOTERM_CC_DIRECT	GO:0005938~cell cortex	2.25E-04
GOTERM_CC_DIRECT	GO:0008250~oligosaccharyltransferase complex	2.76E-04
GOTERM_CC_DIRECT	GO:0032991~macromolecular complex	2.86E-04

GOTERM_CC_DIRECT	GO:0042470~melanosome	4.21E-04
GOTERM_CC_DIRECT	GO:0000792~heterochromatin	4.81E-04
GOTERM_CC_DIRECT	GO:0005769~early endosome	4.99E-04
GOTERM_CC_DIRECT	GO:0031090~organelle membrane	5.21E-04
GOTERM_CC_DIRECT	GO:0045177~apical part of cell	6.55E-04
GOTERM_CC_DIRECT	GO:0005912~adherens junction	7.03E-04
GOTERM_CC_DIRECT	GO:0031252~cell leading edge	7.91E-04
GOTERM_CC_DIRECT	GO:0097526~spliceosomal tri-snRNP complex	9.74E-04
GOTERM_CC_DIRECT	GO:0098685~Schaffer collateral - CA1 synapse	0.001059124
GOTERM_CC_DIRECT	GO:0032040~small-subunit processome	0.001066086
GOTERM_CC_DIRECT	GO:0005680~anaphase-promoting complex	0.001138385
GOTERM_CC_DIRECT	GO:0030136~clathrin-coated vesicle	0.001292682
GOTERM_CC_DIRECT	GO:0090734~site of DNA damage	0.001375316
GOTERM_CC_DIRECT	GO:0097431~mitotic spindle pole	0.001393562
GOTERM_CC_DIRECT	GO:0071011~precatalytic spliceosome	0.001585782
GOTERM_CC_DIRECT	GO:0035267~NuA4 histone acetyltransferase complex	0.001599013
GOTERM_CC_DIRECT	GO:0032391~photoreceptor connecting cilium	0.001681232
GOTERM_CC_DIRECT	GO:0005682~U5 snRNP	0.002021801
GOTERM_CC_DIRECT	GO:0035145~exon-exon junction complex	0.002021801
GOTERM_CC_DIRECT	GO:0030665~clathrin-coated vesicle membrane	0.002021801
GOTERM_CC_DIRECT	GO:0005643~nuclear pore	0.002071364
GOTERM_CC_DIRECT	GO:1990023~mitotic spindle midzone	0.002095409
GOTERM_CC_DIRECT	GO:0034709~methylosome	0.002095409
GOTERM_CC_DIRECT	GO:0005687~U4 snRNP	0.002095409
GOTERM_CC_DIRECT	GO:0010008~endosome membrane	0.002198983
GOTERM_CC_DIRECT	GO:0000791~euchromatin	0.002316148
GOTERM_CC_DIRECT	GO:0002102~podosome	0.002807497
GOTERM_CC_DIRECT	GO:0071339~MLL1 complex	0.002879176
GOTERM_CC_DIRECT	GO:0001940~male pronucleus	0.002898394
GOTERM_CC_DIRECT	GO:0000793~condensed chromosome	0.003609202
GOTERM_CC_DIRECT	GO:0035102~PRC1 complex	0.003609202
GOTERM_CC_DIRECT	GO:1990391~DNA repair complex	0.003835435
GOTERM_CC_DIRECT	GO:0032580~Golgi cisterna membrane	0.003937183
GOTERM_CC_DIRECT	GO:0014069~postsynaptic density	0.004039817
GOTERM_CC_DIRECT	GO:0098793~presynapse	0.004297818
GOTERM_CC_DIRECT	GO:0005815~microtubule organizing center	0.004432854
GOTERM_CC_DIRECT	GO:0031463~Cul3-RING ubiquitin ligase complex	0.004432854
GOTERM_CC_DIRECT	GO:0005663~DNA replication factor C complex	0.004531706
GOTERM_CC_DIRECT	GO:0005765~lysosomal membrane	0.004831548
GOTERM_CC_DIRECT	GO:0031616~spindle pole centrosome	0.005748473
GOTERM_CC_DIRECT	GO:0097546~ciliary base	0.005905299
GOTERM_CC_DIRECT	GO:0015630~microtubule cytoskeleton	0.005962982
GOTERM_CC_DIRECT	GO:0070971~endoplasmic reticulum exit site	0.006010024
GOTERM_CC_DIRECT	GO:0016514~SWI/SNF complex	0.006010024

GOTERM_CC_DIRECT	GO:0005637~nuclear inner membrane	0.006387111
GOTERM_CC_DIRECT	GO:0016442~RISC complex	0.006484486
GOTERM_CC_DIRECT	GO:0005875~microtubule associated complex	0.006484486
GOTERM_CC_DIRECT	GO:0030663~COPI-coated vesicle membrane	0.00657984
GOTERM_CC_DIRECT	GO:0016580~Sin3 complex	0.00657984
GOTERM_CC_DIRECT	GO:0030991~intraciliary transport particle A	0.00657984
GOTERM_CC_DIRECT	GO:0005664~nuclear origin of replication recognition complex	0.00657984
GOTERM_CC_DIRECT	GO:0036038~MKS complex	0.006921457
GOTERM_CC_DIRECT	GO:0032039~integrator complex	0.007281872
GOTERM_CC_DIRECT	GO:0005801~cis-Golgi network	0.007742345
GOTERM_CC_DIRECT	GO:0001673~male germ cell nucleus	0.008006425
GOTERM_CC_DIRECT	GO:0097730~non-motile cilium	0.008420829
GOTERM_CC_DIRECT	GO:0030904~retromer complex	0.008729811
GOTERM_CC_DIRECT	GO:0000307~cyclin-dependent protein kinase holoenzyme complex	0.009213266
GOTERM_CC_DIRECT	GO:0030127~COPII vesicle coat	0.010284745
GOTERM_CC_DIRECT	GO:0005652~nuclear lamina	0.011647844
GOTERM_CC_DIRECT	GO:0005662~DNA replication factor A complex	0.011747506
GOTERM_CC_DIRECT	GO:0030126~COPI vesicle coat	0.011747506
GOTERM_CC_DIRECT	GO:0071564~npBAF complex	0.012615294
GOTERM_CC_DIRECT	GO:0031981~nuclear lumen	0.012615294
GOTERM_CC_DIRECT	GO:0032133~chromosome passenger complex	0.012615294
GOTERM_CC_DIRECT	GO:0000796~condensin complex	0.012615294
GOTERM_CC_DIRECT	GO:0019773~proteasome core complex, alpha-subunit complex	0.012615294
GOTERM_CC_DIRECT	GO:0001939~female pronucleus	0.012615294
GOTERM_CC_DIRECT	GO:0034719~SMN-Sm protein complex	0.012615294
GOTERM_CC_DIRECT	GO:0001917~photoreceptor inner segment	0.012694084
GOTERM_CC_DIRECT	GO:0030992~intraciliary transport particle B	0.012729101
GOTERM_CC_DIRECT	GO:0005839~proteasome core complex	0.012729101
GOTERM_CC_DIRECT	GO:0000145~exocyst	0.015476833
GOTERM_CC_DIRECT	GO:0000794~condensed nuclear chromosome	0.015916593
GOTERM_CC_DIRECT	GO:0016592~mediator complex	0.01602729
GOTERM_CC_DIRECT	GO:0005667~transcription factor complex	0.01689068
GOTERM_CC_DIRECT	GO:0030864~cortical actin cytoskeleton	0.017996239
GOTERM_CC_DIRECT	GO:0043240~Fanconi anaemia nuclear complex	0.018529281
GOTERM_CC_DIRECT	GO:1990498~mitotic spindle microtubule	0.018529281
GOTERM_CC_DIRECT	GO:0043596~nuclear replication fork	0.018529281
GOTERM_CC_DIRECT	GO:0035869~ciliary transition zone	0.020142024
GOTERM_CC_DIRECT	GO:0035101~FACT complex	0.020313978
GOTERM_CC_DIRECT	GO:0070603~SWI/SNF superfamily-type complex	0.020313978
GOTERM_CC_DIRECT	GO:0031262~Ndc80 complex	0.020313978
GOTERM_CC_DIRECT	GO:0033597~mitotic checkpoint complex	0.020313978

GOTERM_CC_DIRECT	GO:0005784~Sec61 translocon complex	0.020313978
GOTERM_CC_DIRECT	GO:0005658~alpha DNA polymerase:primase complex	0.020313978
GOTERM_CC_DIRECT	GO:0000118~histone deacetylase complex	0.020390597
GOTERM_CC_DIRECT	GO:0030123~AP-3 adaptor complex	0.021520403
GOTERM_CC_DIRECT	GO:0030131~clathrin adaptor complex	0.021520403
GOTERM_CC_DIRECT	GO:0030122~AP-2 adaptor complex	0.021520403
GOTERM_CC_DIRECT	GO:0031253~cell projection membrane	0.021520403
GOTERM_CC_DIRECT	GO:0005770~late endosome	0.021983709
GOTERM_CC_DIRECT	GO:0043197~dendritic spine	0.022203573
GOTERM_CC_DIRECT	GO:0031514~motile cilium	0.022393463
GOTERM_CC_DIRECT	GO:0017053~transcriptional repressor complex	0.022603078
GOTERM_CC_DIRECT	GO:0034663~endoplasmic reticulum chaperone complex	0.023313777
GOTERM_CC_DIRECT	GO:0042382~paraspeckles	0.023313777
GOTERM_CC_DIRECT	GO:0034715~pICln-Sm protein complex	0.023313777
GOTERM_CC_DIRECT	GO:0005871~kinesin complex	0.024472233
GOTERM_CC_DIRECT	GO:0070847~core mediator complex	0.025553874
GOTERM_CC_DIRECT	GO:0005657~replication fork	0.025553874
GOTERM_CC_DIRECT	GO:0005930~axoneme	0.025973593
GOTERM_CC_DIRECT	GO:0043202~lysosomal lumen	0.027562877
GOTERM_CC_DIRECT	GO:0090575~RNA polymerase II transcription factor complex	0.029160261
GOTERM_CC_DIRECT	GO:0015030~Cajal body	0.030968954
GOTERM_CC_DIRECT	GO:0060170~ciliary membrane	0.030968954
GOTERM_CC_DIRECT	GO:0005802~trans-Golgi network	0.031980296
GOTERM_CC_DIRECT	GO:0101031~chaperone complex	0.032516266
GOTERM_CC_DIRECT	GO:0032797~SMN complex	0.033686191
GOTERM_CC_DIRECT	GO:0071565~nBAF complex	0.033686191
GOTERM_CC_DIRECT	GO:0000502~proteasome complex	0.033966273
GOTERM_CC_DIRECT	GO:0030027~lamellipodium	0.034841355
GOTERM_CC_DIRECT	GO:0031594~neuromuscular junction	0.036140477
GOTERM_CC_DIRECT	GO:0000228~nuclear chromosome	0.039081632
GOTERM_CC_DIRECT	GO:0030121~AP-1 adaptor complex	0.040047714
GOTERM_CC_DIRECT	GO:0005925~focal adhesion	0.040926969
GOTERM_CC_DIRECT	GO:0016605~PML body	0.04112144
GOTERM_CC_DIRECT	GO:0016589~NURF complex	0.043987386
GOTERM_CC_DIRECT	GO:0070937~CRD-mediated mRNA stability complex	0.043987386
GOTERM_CC_DIRECT	GO:0000811~GINS complex	0.043987386
GOTERM_CC_DIRECT	GO:0098839~postsynaptic density membrane	0.044642769
GOTERM_CC_DIRECT	GO:0043231~intracellular membrane-bounded organelle	0.045497584
GOTERM_CC_DIRECT	GO:0000813~ESCRT I complex	0.04935668
GOTERM_CC_DIRECT	GO:0010369~chromocenter	0.04935668
GOTERM_CC_DIRECT	GO:0030125~clathrin vesicle coat	0.04935668
GOTERM_CC_DIRECT	GO:0044615~nuclear pore nuclear basket	0.04935668
GOTERM_MF_DIRECT	GO:0005515~protein binding	6.17E-22

GOTERM_MF_DIRECT	GO:0005524~ATP binding	3.78E-20
GOTERM_MF_DIRECT	GO:0003682~chromatin binding	6.12E-15
GOTERM_MF_DIRECT	GO:0042393~histone binding	7.07E-14
GOTERM_MF_DIRECT	GO:0003723~RNA binding	2.58E-12
GOTERM_MF_DIRECT	GO:0005096~GTPase activator activity	2.38E-11
GOTERM_MF_DIRECT	GO:0016787~hydrolase activity	4.69E-10
GOTERM_MF_DIRECT	GO:0042802~identical protein binding	2.36E-09
GOTERM_MF_DIRECT	GO:0017116~single-stranded DNA-dependent ATP-dependent DNA helicase activity	1.04E-08
GOTERM_MF_DIRECT	GO:0008017~microtubule binding	1.12E-08
GOTERM_MF_DIRECT	GO:0019904~protein domain specific binding	2.48E-08
GOTERM_MF_DIRECT	GO:0019901~protein kinase binding	9.71E-08
GOTERM_MF_DIRECT	GO:0004674~protein serine/threonine kinase activity	3.19E-07
GOTERM_MF_DIRECT	GO:0003697~single-stranded DNA binding	6.73E-07
GOTERM_MF_DIRECT	GO:0003677~DNA binding	8.94E-07
GOTERM_MF_DIRECT	GO:0031267~small GTPase binding	1.48E-06
GOTERM_MF_DIRECT	GO:0003729~mRNA binding	3.25E-06
GOTERM_MF_DIRECT	GO:0003713~transcription coactivator activity	3.87E-06
GOTERM_MF_DIRECT	GO:0003724~RNA helicase activity	5.20E-06
GOTERM_MF_DIRECT	GO:0004672~protein kinase activity	6.60E-06
GOTERM_MF_DIRECT	GO:0035064~methylated histone binding	8.81E-06
GOTERM_MF_DIRECT	GO:0000400~four-way junction DNA binding	1.20E-05
GOTERM_MF_DIRECT	GO:0042803~protein homodimerization activity	1.21E-05
GOTERM_MF_DIRECT	GO:0003688~DNA replication origin binding	4.59E-05
GOTERM_MF_DIRECT	GO:0003712~transcription cofactor activity	6.68E-05
GOTERM_MF_DIRECT	GO:0003676~nucleic acid binding	8.53E-05
GOTERM_MF_DIRECT	GO:0004386~helicase activity	8.90E-05
GOTERM_MF_DIRECT	GO:0003678~DNA helicase activity	1.22E-04
GOTERM_MF_DIRECT	GO:0046875~ephrin receptor binding	1.28E-04
GOTERM_MF_DIRECT	GO:0042162~telomeric DNA binding	1.28E-04
GOTERM_MF_DIRECT	GO:0097718~disordered domain specific binding	1.29E-04
GOTERM_MF_DIRECT	GO:0019899~enzyme binding	3.05E-04
GOTERM_MF_DIRECT	GO:0031491~nucleosome binding	3.09E-04
GOTERM_MF_DIRECT	GO:0003777~microtubule motor activity	3.24E-04
GOTERM_MF_DIRECT	GO:0051010~microtubule plus-end binding	3.59E-04
GOTERM_MF_DIRECT	GO:0042826~histone deacetylase binding	3.65E-04
GOTERM_MF_DIRECT	GO:0030674~protein binding, bridging	4.54E-04
GOTERM_MF_DIRECT	GO:0005200~structural constituent of cytoskeleton	5.85E-04
GOTERM_MF_DIRECT	GO:0019894~kinesin binding	5.91E-04
GOTERM_MF_DIRECT	GO:0003725~double-stranded RNA binding	6.83E-04
GOTERM_MF_DIRECT	GO:0010997~anaphase-promoting complex binding	7.01E-04
GOTERM_MF_DIRECT	GO:0003689~DNA clamp loader activity	7.01E-04
GOTERM_MF_DIRECT	GO:1990841~promoter-specific chromatin binding	7.20E-04
GOTERM_MF_DIRECT	GO:0016887~ATPase activity	8.36E-04

GOTERM_MF_DIRECT	GO:0003714~transcription corepressor activity	9.23E-04
GOTERM_MF_DIRECT	GO:0003684~damaged DNA binding	9.55E-04
GOTERM_MF_DIRECT	GO:0035173~histone kinase activity	9.88E-04
GOTERM_MF_DIRECT	GO:0030145~manganese ion binding	0.001010846
GOTERM_MF_DIRECT	GO:0005049~nuclear export signal receptor activity	0.001053425
GOTERM_MF_DIRECT	GO:0046872~metal ion binding	0.001656234
GOTERM_MF_DIRECT	GO:0030957~Tat protein binding	0.001780023
GOTERM_MF_DIRECT	GO:0015183~L-aspartate transmembrane transporter activity	0.001780023
GOTERM_MF_DIRECT	GO:0051059~NF-kappaB binding	0.002068298
GOTERM_MF_DIRECT	GO:0061575~cyclin-dependent protein serine/threonine kinase activator activity	0.002184012
GOTERM_MF_DIRECT	GO:0016301~kinase activity	0.002557317
GOTERM_MF_DIRECT	GO:0017056~structural constituent of nuclear pore	0.002731288
GOTERM_MF_DIRECT	GO:0016757~transferase activity, transferring glycosyl groups	0.002840049
GOTERM_MF_DIRECT	GO:0016740~transferase activity	0.003030017
GOTERM_MF_DIRECT	GO:1990756~protein binding, bridging involved in substrate recognition for ubiquitination	0.003132531
GOTERM_MF_DIRECT	GO:0051015~actin filament binding	0.003251164
GOTERM_MF_DIRECT	GO:0051213~dioxygenase activity	0.003672067
GOTERM_MF_DIRECT	GO:0051082~unfolded protein binding	0.003672608
GOTERM_MF_DIRECT	GO:0003730~mRNA 3'-UTR binding	0.004002931
GOTERM_MF_DIRECT	GO:0005112~Notch binding	0.005042864
GOTERM_MF_DIRECT	GO:0048027~mRNA 5'-UTR binding	0.005044745
GOTERM_MF_DIRECT	GO:0004842~ubiquitin-protein transferase activity	0.005154929
GOTERM_MF_DIRECT	GO:0035615~clathrin adaptor activity	0.005856934
GOTERM_MF_DIRECT	GO:0003756~protein disulfide isomerase activity	0.005856934
GOTERM_MF_DIRECT	GO:0016538~cyclin-dependent protein serine/threonine kinase regulator activity	0.006058288
GOTERM_MF_DIRECT	GO:0008168~methyltransferase activity	0.006163421
GOTERM_MF_DIRECT	GO:0043014~alpha-tubulin binding	0.006340576
GOTERM_MF_DIRECT	GO:0035091~phosphatidylinositol binding	0.006823483
GOTERM_MF_DIRECT	GO:0000166~nucleotide binding	0.007989903
GOTERM_MF_DIRECT	GO:0070182~DNA polymerase binding	0.008889932
GOTERM_MF_DIRECT	GO:0030515~snoRNA binding	0.008889932
GOTERM_MF_DIRECT	GO:0042054~histone methyltransferase activity	0.010455032
GOTERM_MF_DIRECT	GO:0004407~histone deacetylase activity	0.010455032
GOTERM_MF_DIRECT	GO:0008195~phosphatidate phosphatase activity	0.010455032
GOTERM_MF_DIRECT	GO:0002039~p53 binding	0.011347454
GOTERM_MF_DIRECT	GO:0008270~zinc ion binding	0.011720559
GOTERM_MF_DIRECT	GO:0000287~magnesium ion binding	0.011733173
GOTERM_MF_DIRECT	GO:0050681~androgen receptor binding	0.01174816
GOTERM_MF_DIRECT	GO:0051787~misfolded protein binding	0.01174816

GOTERM_MF_DIRECT	GO:0003924~GTPase activity	0.011772994
GOTERM_MF_DIRECT	GO:0015616~DNA translocase activity	0.011775841
GOTERM_MF_DIRECT	GO:0016799~hydrolase activity, hydrolyzing N-glycosyl compounds	0.011775841
GOTERM_MF_DIRECT	GO:0035252~UDP-xylosyltransferase activity	0.011775841
GOTERM_MF_DIRECT	GO:0003779~actin binding	0.01217887
GOTERM_MF_DIRECT	GO:1990247~N6-methyladenosine-containing RNA binding	0.012777943
GOTERM_MF_DIRECT	GO:0045134~uridine-diphosphatase activity	0.012777943
GOTERM_MF_DIRECT	GO:0001223~transcription coactivator binding	0.012896835
GOTERM_MF_DIRECT	GO:0031625~ubiquitin protein ligase binding	0.013014416
GOTERM_MF_DIRECT	GO:0003755~peptidyl-prolyl cis-trans isomerase activity	0.013017086
GOTERM_MF_DIRECT	GO:0019900~kinase binding	0.013555498
GOTERM_MF_DIRECT	GO:0043130~ubiquitin binding	0.014470162
GOTERM_MF_DIRECT	GO:0005525~GTP binding	0.014895479
GOTERM_MF_DIRECT	GO:0031418~L-ascorbic acid binding	0.018230058
GOTERM_MF_DIRECT	GO:0005540~hyaluronic acid binding	0.019745254
GOTERM_MF_DIRECT	GO:0030374~ligand-dependent nuclear receptor transcription coactivator activity	0.019852561
GOTERM_MF_DIRECT	GO:0046923~ER retention sequence binding	0.020487426
GOTERM_MF_DIRECT	GO:0032357~oxidized purine DNA binding	0.020487426
GOTERM_MF_DIRECT	GO:0004693~cyclin-dependent protein serine/threonine kinase activity	0.020539601
GOTERM_MF_DIRECT	GO:0017147~Wnt-protein binding	0.020817255
GOTERM_MF_DIRECT	GO:0003690~double-stranded DNA binding	0.020973058
GOTERM_MF_DIRECT	GO:0004382~guanosine-diphosphatase activity	0.021787175
GOTERM_MF_DIRECT	GO:0043139~5'-3' DNA helicase activity	0.021787175
GOTERM_MF_DIRECT	GO:0008574~ATP-dependent microtubule motor activity, plus-end-directed	0.022632842
GOTERM_MF_DIRECT	GO:0051018~protein kinase A binding	0.022632842
GOTERM_MF_DIRECT	GO:0005123~death receptor binding	0.023558697
GOTERM_MF_DIRECT	GO:0015075~ion transmembrane transporter activity	0.023558697
GOTERM_MF_DIRECT	GO:0061676~importin-alpha family protein binding	0.023558697
GOTERM_MF_DIRECT	GO:0050750~low-density lipoprotein particle receptor binding	0.024877317
GOTERM_MF_DIRECT	GO:0030165~PDZ domain binding	0.024931817
GOTERM_MF_DIRECT	GO:0031072~heat shock protein binding	0.031620898
GOTERM_MF_DIRECT	GO:0031435~mitogen-activated protein kinase kinase kinase binding	0.034087274
GOTERM_MF_DIRECT	GO:0032452~histone demethylase activity	0.034087274
GOTERM_MF_DIRECT	GO:0004812~aminoacyl-tRNA ligase activity	0.039599112
GOTERM_MF_DIRECT	GO:0051879~Hsp90 protein binding	0.04004915
GOTERM_MF_DIRECT	GO:0098505~G-rich strand telomeric DNA binding	0.040449406
GOTERM_MF_DIRECT	GO:0036002~pre-mRNA binding	0.040449406

GOTERM_MF_DIRECT	GO:0044877~macromolecular complex binding	0.041221518
GOTERM_MF_DIRECT	GO:0051219~phosphoprotein binding	0.041479436
GOTERM_MF_DIRECT	GO:0004653~polypeptide N-acetylgalactosaminyltransferase activity	0.042124728
GOTERM_MF_DIRECT	GO:0015035~protein disulfide oxidoreductase activity	0.042860004
GOTERM_MF_DIRECT	GO:0070851~growth factor receptor binding	0.044342965
GOTERM_MF_DIRECT	GO:0004694~eukaryotic translation initiation factor 2alpha kinase activity	0.044342965
GOTERM_MF_DIRECT	GO:0045145~single-stranded DNA 5'-3' exodeoxyribonuclease activity	0.044342965
KEGG_PATHWAY	ssc04110:Cell cycle	1.11E-20
KEGG_PATHWAY	ssc03040:Spliceosome	4.62E-14
KEGG_PATHWAY	ssc03030:DNA replication	2.10E-13
KEGG_PATHWAY	ssc03013:Nucleocytoplasmic transport	9.80E-11
KEGG_PATHWAY	ssc04144:Endocytosis	1.52E-10
KEGG_PATHWAY	ssc04141:Protein processing in endoplasmic reticulum	2.27E-10
KEGG_PATHWAY	ssc03082:ATP-dependent chromatin remodeling	1.45E-08
KEGG_PATHWAY	ssc03410:Base excision repair	3.73E-08
KEGG_PATHWAY	ssc00510:N-Glycan biosynthesis	4.87E-08
KEGG_PATHWAY	ssc00513:Various types of N-glycan biosynthesis	1.12E-07
KEGG_PATHWAY	ssc03430:Mismatch repair	3.09E-07
KEGG_PATHWAY	ssc00514:Other types of O-glycan biosynthesis	1.01E-06
KEGG_PATHWAY	ssc05100:Bacterial invasion of epithelial cells	1.76E-06
KEGG_PATHWAY	ssc04142:Lysosome	1.83E-06
KEGG_PATHWAY	ssc01232:Nucleotide metabolism	3.04E-06
KEGG_PATHWAY	ssc03460:Fanconi anemia pathway	1.05E-05
KEGG_PATHWAY	ssc03440:Homologous recombination	1.21E-05
KEGG_PATHWAY	ssc00240:Pyrimidine metabolism	3.12E-05
KEGG_PATHWAY	ssc01100:Metabolic pathways	3.38E-05
KEGG_PATHWAY	ssc03008:Ribosome biogenesis in eukaryotes	3.58E-05
KEGG_PATHWAY	ssc05203:Viral carcinogenesis	4.61E-05
KEGG_PATHWAY	ssc03083:Polycomb repressive complex	7.11E-05
KEGG_PATHWAY	ssc04360:Axon guidance	2.49E-04
KEGG_PATHWAY	ssc04120:Ubiquitin mediated proteolysis	3.94E-04
KEGG_PATHWAY	ssc00100:Steroid biosynthesis	4.06E-04
KEGG_PATHWAY	ssc04114:Oocyte meiosis	8.16E-04
KEGG_PATHWAY	ssc00310:Lysine degradation	0.001039685
KEGG_PATHWAY	ssc04340:Hedgehog signaling pathway	0.001355341
KEGG_PATHWAY	ssc04330:Notch signaling pathway	0.001592921
KEGG_PATHWAY	ssc04914:Progesterone-mediated oocyte maturation	0.001662968
KEGG_PATHWAY	ssc00230:Purine metabolism	0.001857209
KEGG_PATHWAY	ssc00534:Glycosaminoglycan biosynthesis - heparan sulfate / heparin	0.002820516
KEGG_PATHWAY	ssc00531:Glycosaminoglycan degradation	0.003299668

KEGG_PATHWAY	ssc04721:Synaptic vesicle cycle	0.003426803
KEGG_PATHWAY	ssc05225:Hepatocellular carcinoma	0.003558873
KEGG_PATHWAY	ssc03420:Nucleotide excision repair	0.004631994
KEGG_PATHWAY	ssc04510:Focal adhesion	0.00471357
KEGG_PATHWAY	ssc04666:Fc gamma R-mediated phagocytosis	0.004896772
KEGG_PATHWAY	ssc03015:mRNA surveillance pathway	0.005865927
KEGG_PATHWAY	ssc04390:Hippo signaling pathway	0.006573521
KEGG_PATHWAY	ssc00515:Mannose type O-glycan biosynthesis	0.006900989
KEGG_PATHWAY	ssc04814:Motor proteins	0.006976032
KEGG_PATHWAY	ssc04810:Regulation of actin cytoskeleton	0.008624085
KEGG_PATHWAY	ssc05165:Human papillomavirus infection	0.008697297
KEGG_PATHWAY	ssc04218:Cellular senescence	0.009793263
KEGG_PATHWAY	ssc04961:Endocrine and other factor-regulated calcium reabsorption	0.010138262
KEGG_PATHWAY	ssc05014:Amyotrophic lateral sclerosis	0.011483597
KEGG_PATHWAY	ssc05166:Human T-cell leukemia virus 1 infection	0.015058071
KEGG_PATHWAY	ssc00900:Terpenoid backbone biosynthesis	0.016104981
KEGG_PATHWAY	ssc04015:Rap1 signaling pathway	0.017363984
KEGG_PATHWAY	ssc00480:Glutathione metabolism	0.018563149
KEGG_PATHWAY	ssc04115:p53 signaling pathway	0.020416361
KEGG_PATHWAY	ssc05205:Proteoglycans in cancer	0.020889552
KEGG_PATHWAY	ssc00983:Drug metabolism - other enzymes	0.021417057
KEGG_PATHWAY	ssc01250:Biosynthesis of nucleotide sugars	0.021892101
KEGG_PATHWAY	ssc03050:Proteasome	0.02256513
KEGG_PATHWAY	ssc03018:RNA degradation	0.023049108
KEGG_PATHWAY	ssc05220:Chronic myeloid leukemia	0.023773573
KEGG_PATHWAY	ssc05161:Hepatitis B	0.024349482
KEGG_PATHWAY	ssc04520:Adherens junction	0.027215063
KEGG_PATHWAY	ssc01240:Biosynthesis of cofactors	0.032454405
KEGG_PATHWAY	ssc00532:Glycosaminoglycan biosynthesis - chondroitin sulfate / dermatan sulfate	0.035630015
KEGG_PATHWAY	ssc00533:Glycosaminoglycan biosynthesis - keratan sulfate	0.03753408
KEGG_PATHWAY	ssc00670:One carbon pool by folate	0.043291327
KEGG_PATHWAY	ssc00600:Sphingolipid metabolism	0.045108699
KEGG_PATHWAY	ssc00520:Amino sugar and nucleotide sugar metabolism	0.046426568

cluster10

Category	Term	PValue
GOTERM_BP_DIRECT	GO:0001558~regulation of cell growth	5.59E-05
GOTERM_BP_DIRECT	GO:0032259~methylation	1.29E-04
GOTERM_BP_DIRECT	GO:0090090~negative regulation of canonical Wnt signaling pathway	2.08E-04

GOTERM_BP_DIRECT	GO:0032481~positive regulation of type I interferon production	4.88E-04
GOTERM_BP_DIRECT	GO:0001701~in utero embryonic development	5.46E-04
GOTERM_BP_DIRECT	GO:0010629~negative regulation of gene expression	0.001298077
GOTERM_BP_DIRECT	GO:1904888~cranial skeletal system development	0.001937637
GOTERM_BP_DIRECT	GO:1903238~positive regulation of leukocyte tethering or rolling	0.002245076
GOTERM_BP_DIRECT	GO:1990481~mRNA pseudouridine synthesis	0.002245076
GOTERM_BP_DIRECT	GO:0010977~negative regulation of neuron projection development	0.002570243
GOTERM_BP_DIRECT	GO:0140361~cyclic-GMP-AMP transmembrane import across plasma membrane	0.005792174
GOTERM_BP_DIRECT	GO:0090162~establishment of epithelial cell polarity	0.00744591
GOTERM_BP_DIRECT	GO:0005975~carbohydrate metabolic process	0.008614061
GOTERM_BP_DIRECT	GO:0016310~phosphorylation	0.009441017
GOTERM_BP_DIRECT	GO:0001933~negative regulation of protein phosphorylation	0.010285457
GOTERM_BP_DIRECT	GO:0046488~phosphatidylinositol metabolic process	0.01056663
GOTERM_BP_DIRECT	GO:0034214~protein hexamerization	0.014587596
GOTERM_BP_DIRECT	GO:1903428~positive regulation of reactive oxygen species biosynthetic process	0.014587596
GOTERM_BP_DIRECT	GO:0009314~response to radiation	0.014587596
GOTERM_BP_DIRECT	GO:0000165~MAPK cascade	0.014869646
GOTERM_BP_DIRECT	GO:0032868~response to insulin	0.014905159
GOTERM_BP_DIRECT	GO:0006904~vesicle docking involved in exocytosis	0.015983288
GOTERM_BP_DIRECT	GO:0034220~monoatomic ion transmembrane transport	0.016596711
GOTERM_BP_DIRECT	GO:0048863~stem cell differentiation	0.017101119
GOTERM_BP_DIRECT	GO:0050727~regulation of inflammatory response	0.017344367
GOTERM_BP_DIRECT	GO:0006654~phosphatidic acid biosynthetic process	0.017358202
GOTERM_BP_DIRECT	GO:0031669~cellular response to nutrient levels	0.017358202
GOTERM_BP_DIRECT	GO:0006468~protein phosphorylation	0.018152412
GOTERM_BP_DIRECT	GO:0006364~rRNA processing	0.019103559
GOTERM_BP_DIRECT	GO:0050729~positive regulation of inflammatory response	0.020483388
GOTERM_BP_DIRECT	GO:0031529~ruffle organization	0.020758857
GOTERM_BP_DIRECT	GO:2000234~positive regulation of rRNA processing	0.020758857
GOTERM_BP_DIRECT	GO:0019722~calcium-mediated signaling	0.020990046

GOTERM_BP_DIRECT	GO:0048144~fibroblast proliferation	0.021677731
GOTERM_BP_DIRECT	GO:0032743~positive regulation of interleukin-2 production	0.021677731
GOTERM_BP_DIRECT	GO:0046854~phosphatidylinositol phosphate biosynthetic process	0.022158519
GOTERM_BP_DIRECT	GO:0060325~face morphogenesis	0.022985738
GOTERM_BP_DIRECT	GO:0051123~RNA polymerase II preinitiation complex assembly	0.022985738
GOTERM_BP_DIRECT	GO:0007283~spermatogenesis	0.02309436
GOTERM_BP_DIRECT	GO:0031175~neuron projection development	0.024863735
GOTERM_BP_DIRECT	GO:0071230~cellular response to amino acid stimulus	0.025038872
GOTERM_BP_DIRECT	GO:1902775~mitochondrial large ribosomal subunit assembly	0.02660063
GOTERM_BP_DIRECT	GO:2000439~positive regulation of monocyte extravasation	0.02660063
GOTERM_BP_DIRECT	GO:0043403~skeletal muscle tissue regeneration	0.02660063
GOTERM_BP_DIRECT	GO:0030540~female genitalia development	0.02660063
GOTERM_BP_DIRECT	GO:1903801~L-leucine import across plasma membrane	0.02660063
GOTERM_BP_DIRECT	GO:0015829~valine transport	0.02660063
GOTERM_BP_DIRECT	GO:0045943~positive regulation of transcription by RNA polymerase I	0.027081358
GOTERM_BP_DIRECT	GO:0071711~basement membrane organization	0.027081358
GOTERM_BP_DIRECT	GO:0008045~motor neuron axon guidance	0.027126473
GOTERM_BP_DIRECT	GO:0010976~positive regulation of neuron projection development	0.027482379
GOTERM_BP_DIRECT	GO:0045444~fat cell differentiation	0.027482379
GOTERM_BP_DIRECT	GO:0060322~head development	0.028140132
GOTERM_BP_DIRECT	GO:0031638~zymogen activation	0.028593648
GOTERM_BP_DIRECT	GO:0045815~transcription initiation-coupled chromatin remodeling	0.028593648
GOTERM_BP_DIRECT	GO:0097009~energy homeostasis	0.028593648
GOTERM_BP_DIRECT	GO:0043409~negative regulation of MAPK cascade	0.028593648
GOTERM_BP_DIRECT	GO:0035556~intracellular signal transduction	0.02916394
GOTERM_BP_DIRECT	GO:0007628~adult walking behavior	0.03171246
GOTERM_BP_DIRECT	GO:0006367~transcription initiation at RNA polymerase II promoter	0.03253899
GOTERM_BP_DIRECT	GO:0006954~inflammatory response	0.033580544
GOTERM_BP_DIRECT	GO:0030336~negative regulation of cell migration	0.034057694
GOTERM_BP_DIRECT	GO:0042273~ribosomal large subunit biogenesis	0.035187235
GOTERM_BP_DIRECT	GO:0140374~antiviral innate immune response	0.035187235

GOTERM_BP_DIRECT	GO:0031397~negative regulation of protein ubiquitination	0.035187235
GOTERM_BP_DIRECT	GO:0006334~nucleosome assembly	0.035237793
GOTERM_BP_DIRECT	GO:0046834~lipid phosphorylation	0.036723401
GOTERM_BP_DIRECT	GO:0002082~regulation of oxidative phosphorylation	0.036723401
GOTERM_BP_DIRECT	GO:0009966~regulation of signal transduction	0.036821255
GOTERM_BP_DIRECT	GO:0045893~positive regulation of DNA-templated transcription	0.038209462
GOTERM_BP_DIRECT	GO:0060392~negative regulation of SMAD protein signal transduction	0.039489815
GOTERM_BP_DIRECT	GO:0001655~urogenital system development	0.039489815
GOTERM_BP_DIRECT	GO:0050821~protein stabilization	0.040758059
GOTERM_BP_DIRECT	GO:0007611~learning or memory	0.042261219
GOTERM_BP_DIRECT	GO:0048741~skeletal muscle fiber development	0.042261219
GOTERM_BP_DIRECT	GO:0009749~response to glucose	0.042261219
GOTERM_BP_DIRECT	GO:2000535~regulation of entry of bacterium into host cell	0.042297986
GOTERM_BP_DIRECT	GO:0018023~peptidyl-lysine trimethylation	0.042297986
GOTERM_BP_DIRECT	GO:0033564~anterior/posterior axon guidance	0.042297986
GOTERM_BP_DIRECT	GO:0070100~negative regulation of chemokine-mediated signaling pathway	0.042297986
GOTERM_BP_DIRECT	GO:0038007~netrin-activated signaling pathway	0.042297986
GOTERM_BP_DIRECT	GO:0046883~regulation of hormone secretion	0.042297986
GOTERM_BP_DIRECT	GO:0006811~monoatomic ion transport	0.046199462
GOTERM_BP_DIRECT	GO:0006914~autophagy	0.046199462
GOTERM_BP_DIRECT	GO:0034644~cellular response to UV	0.046424518
GOTERM_BP_DIRECT	GO:0010820~positive regulation of T cell chemotaxis	0.046482565
GOTERM_BP_DIRECT	GO:0045745~positive regulation of G protein-coupled receptor signaling pathway	0.046482565
GOTERM_BP_DIRECT	GO:0033689~negative regulation of osteoblast proliferation	0.046482565
GOTERM_BP_DIRECT	GO:0001510~RNA methylation	0.046482565
GOTERM_BP_DIRECT	GO:0048546~digestive tract morphogenesis	0.046482565
GOTERM_BP_DIRECT	GO:0045945~positive regulation of transcription by RNA polymerase III	0.046482565
GOTERM_BP_DIRECT	GO:0042474~middle ear morphogenesis	0.046720611
GOTERM_BP_DIRECT	GO:0031663~lipopolysaccharide-mediated signaling pathway	0.048237898
GOTERM_BP_DIRECT	GO:0042593~glucose homeostasis	0.048624801
GOTERM_CC_DIRECT	GO:0005829~cytosol	7.54E-07
GOTERM_CC_DIRECT	GO:0045121~membrane raft	7.03E-06
GOTERM_CC_DIRECT	GO:0005730~nucleolus	1.18E-05

GOTERM_CC_DIRECT	GO:0016328~lateral plasma membrane	6.53E-05
GOTERM_CC_DIRECT	GO:0005634~nucleus	2.09E-04
GOTERM_CC_DIRECT	GO:0005737~cytoplasm	2.70E-04
GOTERM_CC_DIRECT	GO:0005654~nucleoplasm	4.77E-04
GOTERM_CC_DIRECT	GO:0005765~lysosomal membrane	5.43E-04
GOTERM_CC_DIRECT	GO:0005739~mitochondrion	9.44E-04
GOTERM_CC_DIRECT	GO:0016323~basolateral plasma membrane	0.001268715
GOTERM_CC_DIRECT	GO:0016324~apical plasma membrane	0.00231449
GOTERM_CC_DIRECT	GO:0031410~cytoplasmic vesicle	0.003762669
GOTERM_CC_DIRECT	GO:0030425~dendrite	0.007580201
GOTERM_CC_DIRECT	GO:0098978~glutamatergic synapse	0.008383178
GOTERM_CC_DIRECT	GO:0031931~TORC1 complex	0.008566815
GOTERM_CC_DIRECT	GO:0005764~lysosome	0.017225109
GOTERM_CC_DIRECT	GO:1990877~FNIP-folliculin RagC/D GAP	0.018580757
GOTERM_CC_DIRECT	GO:0030687~preribosome, large subunit precursor	0.019568893
GOTERM_CC_DIRECT	GO:0005669~transcription factor TFIID complex	0.020832943
GOTERM_CC_DIRECT	GO:0009986~cell surface	0.021091445
GOTERM_CC_DIRECT	GO:0012505~endomembrane system	0.024733476
GOTERM_CC_DIRECT	GO:1990531~phospholipid-translocating ATPase complex	0.025241082
GOTERM_CC_DIRECT	GO:0005622~intracellular anatomical structure	0.025914576
GOTERM_CC_DIRECT	GO:0043229~intracellular organelle	0.028840625
GOTERM_CC_DIRECT	GO:0005794~Golgi apparatus	0.029414537
GOTERM_CC_DIRECT	GO:0005666~RNA polymerase III complex	0.031505236
GOTERM_CC_DIRECT	GO:0032991~protein-containing complex	0.036703739
GOTERM_CC_DIRECT	GO:0098793~presynapse	0.036877632
GOTERM_CC_DIRECT	GO:0000139~Golgi membrane	0.037674209
GOTERM_CC_DIRECT	GO:0005783~endoplasmic reticulum	0.038441988
GOTERM_CC_DIRECT	GO:0045202~synapse	0.039588486
GOTERM_CC_DIRECT	GO:0045211~postsynaptic membrane	0.04354798
GOTERM_CC_DIRECT	GO:0030027~lamellipodium	0.04354798
GOTERM_CC_DIRECT	GO:0031966~mitochondrial membrane	0.043781
GOTERM_CC_DIRECT	GO:0043025~neuronal cell body	0.048483593
GOTERM_CC_DIRECT	GO:0048471~perinuclear region of cytoplasm	0.048678715
GOTERM_CC_DIRECT	GO:0016604~nuclear body	0.049354415
GOTERM_MF_DIRECT	GO:0005515~protein binding	1.38E-08
GOTERM_MF_DIRECT	GO:0005524~ATP binding	6.24E-05
GOTERM_MF_DIRECT	GO:0005225~volume-sensitive anion channel activity	1.17E-04
GOTERM_MF_DIRECT	GO:0046872~metal ion binding	2.31E-04
GOTERM_MF_DIRECT	GO:0000049~tRNA binding	5.15E-04
GOTERM_MF_DIRECT	GO:0044877~protein-containing complex binding	8.53E-04
GOTERM_MF_DIRECT	GO:0004386~helicase activity	0.002649562

GOTERM_MF_DIRECT	GO:0042802~identical protein binding	0.004354179
GOTERM_MF_DIRECT	GO:0016279~protein-lysine N-methyltransferase activity	0.004355282
GOTERM_MF_DIRECT	GO:0140036~ubiquitin-modified protein reader activity	0.007738283
GOTERM_MF_DIRECT	GO:0030971~receptor tyrosine kinase binding	0.00781574
GOTERM_MF_DIRECT	GO:0000182~rDNA binding	0.009908068
GOTERM_MF_DIRECT	GO:0009982~pseudouridine synthase activity	0.010560756
GOTERM_MF_DIRECT	GO:0001786~phosphatidylserine binding	0.011053422
GOTERM_MF_DIRECT	GO:0140297~DNA-binding transcription factor binding	0.012380622
GOTERM_MF_DIRECT	GO:0097110~scaffold protein binding	0.018203981
GOTERM_MF_DIRECT	GO:0004197~cysteine-type endopeptidase activity	0.018577119
GOTERM_MF_DIRECT	GO:0004672~protein kinase activity	0.019043753
GOTERM_MF_DIRECT	GO:0003724~RNA helicase activity	0.024115176
GOTERM_MF_DIRECT	GO:0005254~chloride channel activity	0.025524651
GOTERM_MF_DIRECT	GO:0140359~ABC-type transporter activity	0.026579246
GOTERM_MF_DIRECT	GO:0004016~adenylate cyclase activity	0.028059008
GOTERM_MF_DIRECT	GO:0004143~ATP-dependent diacylglycerol kinase activity	0.037766078
GOTERM_MF_DIRECT	GO:1990782~protein tyrosine kinase binding	0.03853601
GOTERM_MF_DIRECT	GO:0140326~ATPase-coupled intramembrane lipid transporter activity	0.040866666
GOTERM_MF_DIRECT	GO:0016428~tRNA (cytidine-5-)-methyltransferase activity	0.043161873
GOTERM_MF_DIRECT	GO:0140318~protein transporter activity	0.043161873
GOTERM_MF_DIRECT	GO:0005042~netrin receptor activity	0.043161873
GOTERM_MF_DIRECT	GO:0048156~tau protein binding	0.047775343
GOTERM_MF_DIRECT	GO:0008173~RNA methyltransferase activity	0.047775343
GOTERM_MF_DIRECT	GO:0005324~long-chain fatty acid transmembrane transporter activity	0.047775343
GOTERM_MF_DIRECT	GO:0016251~RNA polymerase II general transcription initiation factor activity	0.048526386
KEGG_PATHWAY	ssc04070:Phosphatidylinositol signaling system	2.11E-04
KEGG_PATHWAY	ssc01100:Metabolic pathways	2.15E-04
KEGG_PATHWAY	ssc00562:Inositol phosphate metabolism	9.18E-04
KEGG_PATHWAY	ssc04148:Efferocytosis	0.001374754
KEGG_PATHWAY	ssc03008:Ribosome biogenesis in eukaryotes	0.001988995
KEGG_PATHWAY	ssc00564:Glycerophospholipid metabolism	0.0023012
KEGG_PATHWAY	ssc05132:Salmonella infection	0.00368283
KEGG_PATHWAY	ssc05225:Hepatocellular carcinoma	0.007651053
KEGG_PATHWAY	ssc04150:mTOR signaling pathway	0.008110999
KEGG_PATHWAY	ssc05214:Glioma	0.021857155
KEGG_PATHWAY	ssc05417:Lipid and atherosclerosis	0.023553596

KEGG_PATHWAY	ssc04360:Axon guidance	0.02363362
KEGG_PATHWAY	ssc04310:Wnt signaling pathway	0.024327485
KEGG_PATHWAY	ssc04935:Growth hormone synthesis, secretion and action	0.030129802
KEGG_PATHWAY	ssc03250:Viral life cycle - HIV-1	0.032198627
KEGG_PATHWAY	ssc04062:Chemokine signaling pathway	0.042400917
KEGG_PATHWAY	ssc04072:Phospholipase D signaling pathway	0.047819728
KEGG_PATHWAY	ssc04668:TNF signaling pathway	0.049872006

cluster3&8

Category	Term	PValue
GOTERM_BP_DIRECT	GO:0006357~regulation of transcription by RNA polymerase II	1.07E-09
GOTERM_BP_DIRECT	GO:0000122~negative regulation of transcription by RNA polymerase II	5.86E-06
GOTERM_BP_DIRECT	GO:0006355~regulation of DNA-templated transcription	1.93E-05
GOTERM_BP_DIRECT	GO:0007099~centriole replication	3.26E-04
GOTERM_BP_DIRECT	GO:0045944~positive regulation of transcription by RNA polymerase II	4.99E-04
GOTERM_BP_DIRECT	GO:0060271~cilium assembly	9.39E-04
GOTERM_BP_DIRECT	GO:0018105~peptidyl-serine phosphorylation	9.76E-04
GOTERM_BP_DIRECT	GO:0007030~Golgi organization	0.001069765
GOTERM_BP_DIRECT	GO:0016310~phosphorylation	0.002160193
GOTERM_BP_DIRECT	GO:0060412~ventricular septum morphogenesis	0.00302861
GOTERM_BP_DIRECT	GO:0000381~regulation of alternative mRNA splicing, via spliceosome	0.003080999
GOTERM_BP_DIRECT	GO:0006397~mRNA processing	0.003175145
GOTERM_BP_DIRECT	GO:0007049~cell cycle	0.003641644
GOTERM_BP_DIRECT	GO:0034504~protein localization to nucleus	0.003930602
GOTERM_BP_DIRECT	GO:0071539~protein localization to centrosome	0.004453883
GOTERM_BP_DIRECT	GO:0045893~positive regulation of DNA-templated transcription	0.004630916
GOTERM_BP_DIRECT	GO:0019827~stem cell population maintenance	0.00502642
GOTERM_BP_DIRECT	GO:0006888~endoplasmic reticulum to Golgi vesicle-mediated transport	0.00507025
GOTERM_BP_DIRECT	GO:0043161~proteasome-mediated ubiquitin-dependent protein catabolic process	0.005171623
GOTERM_BP_DIRECT	GO:0007018~microtubule-based movement	0.006110503
GOTERM_BP_DIRECT	GO:0044782~cilium organization	0.006501758
GOTERM_BP_DIRECT	GO:0042274~ribosomal small subunit biogenesis	0.00742734
GOTERM_BP_DIRECT	GO:0001501~skeletal system development	0.007697339
GOTERM_BP_DIRECT	GO:0003151~outflow tract morphogenesis	0.007793115

GOTERM_BP_DIRECT	GO:0007507~heart development	0.00798066
GOTERM_BP_DIRECT	GO:0010457~centriole-centriole cohesion	0.008421479
GOTERM_BP_DIRECT	GO:0048681~negative regulation of axon regeneration	0.008421479
GOTERM_BP_DIRECT	GO:0051149~positive regulation of muscle cell differentiation	0.008637545
GOTERM_BP_DIRECT	GO:0060045~positive regulation of cardiac muscle cell proliferation	0.008708019
GOTERM_BP_DIRECT	GO:0031468~nuclear membrane reassembly	0.009342999
GOTERM_BP_DIRECT	GO:0008045~motor neuron axon guidance	0.009611492
GOTERM_BP_DIRECT	GO:0002682~regulation of immune system process	0.010088568
GOTERM_BP_DIRECT	GO:0008283~cell population proliferation	0.010241862
GOTERM_BP_DIRECT	GO:0008344~adult locomotory behavior	0.010420537
GOTERM_BP_DIRECT	GO:0006974~DNA damage response	0.013463216
GOTERM_BP_DIRECT	GO:0048015~phosphatidylinositol-mediated signaling	0.014873921
GOTERM_BP_DIRECT	GO:0015031~protein transport	0.017675511
GOTERM_BP_DIRECT	GO:0006906~vesicle fusion	0.018156421
GOTERM_BP_DIRECT	GO:0048706~embryonic skeletal system development	0.018452989
GOTERM_BP_DIRECT	GO:0006470~protein dephosphorylation	0.018588838
GOTERM_BP_DIRECT	GO:0032495~response to muramyl dipeptide	0.018729769
GOTERM_BP_DIRECT	GO:0006325~chromatin organization	0.020963321
GOTERM_BP_DIRECT	GO:0007179~transforming growth factor beta receptor signaling pathway	0.022266074
GOTERM_BP_DIRECT	GO:0045292~mRNA cis splicing, via spliceosome	0.022405145
GOTERM_BP_DIRECT	GO:0000398~mRNA splicing, via spliceosome	0.022603582
GOTERM_BP_DIRECT	GO:1904263~positive regulation of TORC1 signaling	0.02492461
GOTERM_BP_DIRECT	GO:0061025~membrane fusion	0.025902931
GOTERM_BP_DIRECT	GO:0008156~negative regulation of DNA replication	0.025902931
GOTERM_BP_DIRECT	GO:0030865~cortical cytoskeleton organization	0.025902931
GOTERM_BP_DIRECT	GO:0090161~Golgi ribbon formation	0.025914885
GOTERM_BP_DIRECT	GO:0051148~negative regulation of muscle cell differentiation	0.025914885
GOTERM_BP_DIRECT	GO:0031509~subtelomeric heterochromatin formation	0.025914885
GOTERM_BP_DIRECT	GO:1901798~positive regulation of signal transduction by p53 class mediator	0.025914885
GOTERM_BP_DIRECT	GO:0051726~regulation of cell cycle	0.026185856
GOTERM_BP_DIRECT	GO:0034446~substrate adhesion-dependent cell spreading	0.027733058
GOTERM_BP_DIRECT	GO:0038127~ERBB signaling pathway	0.028183373
GOTERM_BP_DIRECT	GO:0032915~positive regulation of transforming growth factor beta2 production	0.028183373
GOTERM_BP_DIRECT	GO:0007015~actin filament organization	0.030326278
GOTERM_BP_DIRECT	GO:0046856~phosphatidylinositol dephosphorylation	0.030978888
GOTERM_BP_DIRECT	GO:0045995~regulation of embryonic development	0.032262516
GOTERM_BP_DIRECT	GO:0016925~protein sumoylation	0.032262516

GOTERM_BP_DIRECT	GO:0007605~sensory perception of sound	0.032498275
GOTERM_BP_DIRECT	GO:0006468~protein phosphorylation	0.033065169
GOTERM_BP_DIRECT	GO:0001958~endochondral ossification	0.033821221
GOTERM_BP_DIRECT	GO:0007566~embryo implantation	0.033821221
GOTERM_BP_DIRECT	GO:0070102~interleukin-6-mediated signaling pathway	0.034508678
GOTERM_BP_DIRECT	GO:0097178~ruffle assembly	0.034508678
GOTERM_BP_DIRECT	GO:0045947~negative regulation of translational initiation	0.034508678
GOTERM_BP_DIRECT	GO:0033690~positive regulation of osteoblast proliferation	0.034508678
GOTERM_BP_DIRECT	GO:0000289~nuclear-transcribed mRNA poly(A) tail shortening	0.034508678
GOTERM_BP_DIRECT	GO:0045669~positive regulation of osteoblast differentiation	0.034758986
GOTERM_BP_DIRECT	GO:0007213~G protein-coupled acetylcholine receptor signaling pathway	0.038416015
GOTERM_BP_DIRECT	GO:1900242~regulation of synaptic vesicle endocytosis	0.038416015
GOTERM_BP_DIRECT	GO:0042118~endothelial cell activation	0.038416015
GOTERM_BP_DIRECT	GO:0001657~ureteric bud development	0.040356558
GOTERM_BP_DIRECT	GO:1905515~non-motile cilium assembly	0.040390505
GOTERM_BP_DIRECT	GO:0001503~ossification	0.041736671
GOTERM_BP_DIRECT	GO:0016192~vesicle-mediated transport	0.042040401
GOTERM_BP_DIRECT	GO:0051225~spindle assembly	0.042261009
GOTERM_BP_DIRECT	GO:0001934~positive regulation of protein phosphorylation	0.042635939
GOTERM_BP_DIRECT	GO:0043491~phosphatidylinositol 3-kinase/protein kinase B signal transduction	0.042951666
GOTERM_BP_DIRECT	GO:0043488~regulation of mRNA stability	0.043663469
GOTERM_BP_DIRECT	GO:1901673~regulation of mitotic spindle assembly	0.043663469
GOTERM_BP_DIRECT	GO:0034113~heterotypic cell-cell adhesion	0.043663469
GOTERM_BP_DIRECT	GO:0005977~glycogen metabolic process	0.043663469
GOTERM_BP_DIRECT	GO:0072520~seminiferous tubule development	0.04457495
GOTERM_BP_DIRECT	GO:0001525~angiogenesis	0.044780287
GOTERM_CC_DIRECT	GO:0005634~nucleus	1.97E-16
GOTERM_CC_DIRECT	GO:0005654~nucleoplasm	7.61E-08
GOTERM_CC_DIRECT	GO:0005813~centrosome	1.14E-07
GOTERM_CC_DIRECT	GO:0005814~centriole	5.28E-06
GOTERM_CC_DIRECT	GO:0005802~trans-Golgi network	7.81E-06
GOTERM_CC_DIRECT	GO:0005829~cytosol	1.09E-05
GOTERM_CC_DIRECT	GO:0005737~cytoplasm	1.41E-05
GOTERM_CC_DIRECT	GO:0005730~nucleolus	1.13E-04
GOTERM_CC_DIRECT	GO:0034451~centriolar satellite	2.60E-04

GOTERM_CC_DIRECT	GO:0098556~cytoplasmic side of rough endoplasmic reticulum membrane	2.88E-04
GOTERM_CC_DIRECT	GO:0016604~nuclear body	3.27E-04
GOTERM_CC_DIRECT	GO:0005794~Golgi apparatus	6.40E-04
GOTERM_CC_DIRECT	GO:0005793~endoplasmic reticulum-Golgi intermediate compartment	7.98E-04
GOTERM_CC_DIRECT	GO:0098978~glutamatergic synapse	8.00E-04
GOTERM_CC_DIRECT	GO:0042995~cell projection	0.001231489
GOTERM_CC_DIRECT	GO:0016459~myosin complex	0.00157961
GOTERM_CC_DIRECT	GO:0045202~synapse	0.003475315
GOTERM_CC_DIRECT	GO:0030134~COPII-coated ER to Golgi transport vesicle	0.003674363
GOTERM_CC_DIRECT	GO:0005856~cytoskeleton	0.004647091
GOTERM_CC_DIRECT	GO:0036064~ciliary basal body	0.006099982
GOTERM_CC_DIRECT	GO:0016607~nuclear speck	0.006791288
GOTERM_CC_DIRECT	GO:0030016~myofibril	0.007031938
GOTERM_CC_DIRECT	GO:0014069~postsynaptic density	0.007593636
GOTERM_CC_DIRECT	GO:0009898~cytoplasmic side of plasma membrane	0.008565873
GOTERM_CC_DIRECT	GO:0005840~ribosome	0.009747411
GOTERM_CC_DIRECT	GO:0016592~mediator complex	0.011829281
GOTERM_CC_DIRECT	GO:0009925~basal plasma membrane	0.015334628
GOTERM_CC_DIRECT	GO:0030027~lamellipodium	0.015875702
GOTERM_CC_DIRECT	GO:0031201~SNARE complex	0.016798962
GOTERM_CC_DIRECT	GO:0030018~Z disc	0.017084724
GOTERM_CC_DIRECT	GO:0030054~cell junction	0.018808974
GOTERM_CC_DIRECT	GO:0016363~nuclear matrix	0.020939888
GOTERM_CC_DIRECT	GO:0005834~heterotrimeric G-protein complex	0.021449281
GOTERM_CC_DIRECT	GO:0005911~cell-cell junction	0.02290509
GOTERM_CC_DIRECT	GO:0001725~stress fiber	0.023455598
GOTERM_CC_DIRECT	GO:0032587~ruffle membrane	0.023455598
GOTERM_CC_DIRECT	GO:0000775~chromosome, centromeric region	0.025076679
GOTERM_CC_DIRECT	GO:0031901~early endosome membrane	0.032077685
GOTERM_CC_DIRECT	GO:0000781~chromosome, telomeric region	0.032081056
GOTERM_CC_DIRECT	GO:0000242~pericentriolar material	0.033045244
GOTERM_CC_DIRECT	GO:0005881~cytoplasmic microtubule	0.033094662
GOTERM_CC_DIRECT	GO:0001650~fibrillar center	0.035666628
GOTERM_CC_DIRECT	GO:0031965~nuclear membrane	0.036887226
GOTERM_CC_DIRECT	GO:0031514~motile cilium	0.037405395
GOTERM_CC_DIRECT	GO:0005669~transcription factor TFIID complex	0.038572185
GOTERM_CC_DIRECT	GO:0005694~chromosome	0.038860466
GOTERM_CC_DIRECT	GO:0005930~axoneme	0.040547889
GOTERM_CC_DIRECT	GO:0001518~voltage-gated sodium channel complex	0.040597053
GOTERM_CC_DIRECT	GO:0015934~large ribosomal subunit	0.040597053
GOTERM_CC_DIRECT	GO:0005925~focal adhesion	0.041465253

GOTERM_CC_DIRECT	GO:0005783~endoplasmic reticulum	0.042388592
GOTERM_CC_DIRECT	GO:0005874~microtubule	0.043080982
GOTERM_CC_DIRECT	GO:0010494~cytoplasmic stress granule	0.044084812
GOTERM_CC_DIRECT	GO:0005789~endoplasmic reticulum membrane	0.046891806
GOTERM_CC_DIRECT	GO:0044295~axonal growth cone	0.049109266
GOTERM_MF_DIRECT	GO:0001227~DNA-binding transcription repressor activity, RNA polymerase II-specific	1.38E-10
GOTERM_MF_DIRECT	GO:0000978~RNA polymerase II cis-regulatory region sequence-specific DNA binding	5.61E-10
GOTERM_MF_DIRECT	GO:0000981~DNA-binding transcription factor activity, RNA polymerase II-specific	1.37E-09
GOTERM_MF_DIRECT	GO:0003677~DNA binding	5.18E-09
GOTERM_MF_DIRECT	GO:0046872~metal ion binding	8.89E-09
GOTERM_MF_DIRECT	GO:0005515~protein binding	2.00E-06
GOTERM_MF_DIRECT	GO:0000977~RNA polymerase II transcription regulatory region sequence-specific DNA binding	9.58E-05
GOTERM_MF_DIRECT	GO:0034237~protein kinase A regulatory subunit binding	1.09E-04
GOTERM_MF_DIRECT	GO:0031625~ubiquitin protein ligase binding	0.001022985
GOTERM_MF_DIRECT	GO:0045505~dynein intermediate chain binding	0.00163854
GOTERM_MF_DIRECT	GO:0003924~GTPase activity	0.001712009
GOTERM_MF_DIRECT	GO:0005524~ATP binding	0.002929223
GOTERM_MF_DIRECT	GO:0008017~microtubule binding	0.003047032
GOTERM_MF_DIRECT	GO:0005096~GTPase activator activity	0.003216934
GOTERM_MF_DIRECT	GO:0005525~GTP binding	0.003746341
GOTERM_MF_DIRECT	GO:0042802~identical protein binding	0.004540109
GOTERM_MF_DIRECT	GO:0030506~ankyrin binding	0.006183914
GOTERM_MF_DIRECT	GO:0016853~isomerase activity	0.007155435
GOTERM_MF_DIRECT	GO:0035925~mRNA 3'-UTR AU-rich region binding	0.008289792
GOTERM_MF_DIRECT	GO:0046332~SMAD binding	0.008811017
GOTERM_MF_DIRECT	GO:0004620~phospholipase activity	0.008950511
GOTERM_MF_DIRECT	GO:0003729~mRNA binding	0.009732327
GOTERM_MF_DIRECT	GO:0043130~ubiquitin binding	0.010191293
GOTERM_MF_DIRECT	GO:0061629~RNA polymerase II-specific DNA-binding transcription factor binding	0.013774829
GOTERM_MF_DIRECT	GO:0003682~chromatin binding	0.014324378
GOTERM_MF_DIRECT	GO:0042803~protein homodimerization activity	0.015930602
GOTERM_MF_DIRECT	GO:0060090~molecular adaptor activity	0.021421439
GOTERM_MF_DIRECT	GO:0046982~protein heterodimerization activity	0.022209986
GOTERM_MF_DIRECT	GO:0035064~methylated histone binding	0.023956902
GOTERM_MF_DIRECT	GO:1990817~poly(A) RNA polymerase activity	0.025038593
GOTERM_MF_DIRECT	GO:0008143~poly(A) binding	0.026783817
GOTERM_MF_DIRECT	GO:0017134~fibroblast growth factor binding	0.027418462

GOTERM_MF_DIRECT	GO:0016423~tRNA (guanine) methyltransferase activity	0.027634672
GOTERM_MF_DIRECT	GO:0019901~protein kinase binding	0.028455779
GOTERM_MF_DIRECT	GO:0003700~DNA-binding transcription factor activity	0.02940322
GOTERM_MF_DIRECT	GO:0001228~DNA-binding transcription activator activity, RNA polymerase II-specific	0.029892849
GOTERM_MF_DIRECT	GO:0000976~transcription cis-regulatory region binding	0.031294214
GOTERM_MF_DIRECT	GO:0005509~calcium ion binding	0.032073242
GOTERM_MF_DIRECT	GO:0045295~gamma-catenin binding	0.033384751
GOTERM_MF_DIRECT	GO:0015095~magnesium ion transmembrane transporter activity	0.033384751
GOTERM_MF_DIRECT	GO:0004707~MAP kinase activity	0.033384751
GOTERM_MF_DIRECT	GO:0005484~SNAP receptor activity	0.035243819
GOTERM_MF_DIRECT	GO:0016887~ATP hydrolysis activity	0.040441637
GOTERM_MF_DIRECT	GO:0031683~G-protein beta/gamma-subunit complex binding	0.042045348
GOTERM_MF_DIRECT	GO:0050839~cell adhesion molecule binding	0.044466763
GOTERM_MF_DIRECT	GO:0042169~SH2 domain binding	0.04656414
GOTERM_MF_DIRECT	GO:0004674~protein serine/threonine kinase activity	0.047584185
GOTERM_MF_DIRECT	GO:0003779~actin binding	0.049423728
KEGG_PATHWAY	ssc05168:Herpes simplex virus 1 infection	3.64E-09
KEGG_PATHWAY	ssc03010:Ribosome	3.32E-08
KEGG_PATHWAY	ssc05132:Salmonella infection	4.21E-06
KEGG_PATHWAY	ssc05171:Coronavirus disease - COVID-19	4.21E-06
KEGG_PATHWAY	ssc04520:Adherens junction	1.41E-05
KEGG_PATHWAY	ssc04611:Platelet activation	6.56E-05
KEGG_PATHWAY	ssc04728:Dopaminergic synapse	7.65E-05
KEGG_PATHWAY	ssc04814:Motor proteins	1.45E-04
KEGG_PATHWAY	ssc04713:Circadian entrainment	3.45E-04
KEGG_PATHWAY	ssc04010:MAPK signaling pathway	8.38E-04
KEGG_PATHWAY	ssc04926:Relaxin signaling pathway	0.001019365
KEGG_PATHWAY	ssc04510:Focal adhesion	0.001094539
KEGG_PATHWAY	ssc04020:Calcium signaling pathway	0.001144198
KEGG_PATHWAY	ssc04371:Apelin signaling pathway	0.001203482
KEGG_PATHWAY	ssc04820:Cytoskeleton in muscle cells	0.002233899
KEGG_PATHWAY	ssc04911:Insulin secretion	0.00240538
KEGG_PATHWAY	ssc04014:Ras signaling pathway	0.003000866
KEGG_PATHWAY	ssc03040:Spliceosome	0.003022114
KEGG_PATHWAY	ssc04151:PI3K-Akt signaling pathway	0.003907624
KEGG_PATHWAY	ssc04071:Sphingolipid signaling pathway	0.00438802
KEGG_PATHWAY	ssc04120:Ubiquitin mediated proteolysis	0.0046381
KEGG_PATHWAY	ssc04724:Glutamatergic synapse	0.005204199

KEGG_PATHWAY	ssc04140:Autophagy - animal	0.007035328
KEGG_PATHWAY	ssc04261:Adrenergic signaling in cardiomyocytes	0.007157157
KEGG_PATHWAY	ssc05163:Human cytomegalovirus infection	0.009685935
KEGG_PATHWAY	ssc04924:Renin secretion	0.012364202
KEGG_PATHWAY	ssc05030:Cocaine addiction	0.013529451
KEGG_PATHWAY	ssc03018:RNA degradation	0.014626812
KEGG_PATHWAY	ssc05202:Transcriptional misregulation in cancer	0.016355322
KEGG_PATHWAY	ssc05167:Kaposi sarcoma-associated herpesvirus infection	0.019136302
KEGG_PATHWAY	ssc03420:Nucleotide excision repair	0.019550545
KEGG_PATHWAY	ssc05034:Alcoholism	0.021291896
KEGG_PATHWAY	ssc04512:ECM-receptor interaction	0.022307777
KEGG_PATHWAY	ssc04810:Regulation of actin cytoskeleton	0.026894732
KEGG_PATHWAY	ssc04350:TGF-beta signaling pathway	0.028468938
KEGG_PATHWAY	ssc04144:Endocytosis	0.031713616
KEGG_PATHWAY	ssc05200:Pathways in cancer	0.031935322
KEGG_PATHWAY	ssc04130:SNARE interactions in vesicular transport	0.033045098
KEGG_PATHWAY	ssc00562:Inositol phosphate metabolism	0.034904992
KEGG_PATHWAY	ssc04270:Vascular smooth muscle contraction	0.035189677
KEGG_PATHWAY	ssc05414:Dilated cardiomyopathy	0.036755566
KEGG_PATHWAY	ssc05203:Viral carcinogenesis	0.041872734
KEGG_PATHWAY	ssc03022:Basal transcription factors	0.04216549
KEGG_PATHWAY	ssc04141:Protein processing in endoplasmic reticulum	0.042784496
KEGG_PATHWAY	ssc04621:NOD-like receptor signaling pathway	0.043368316
KEGG_PATHWAY	ssc04921:Oxytocin signaling pathway	0.043848143
KEGG_PATHWAY	ssc04670:Leukocyte transendothelial migration	0.044642285
KEGG_PATHWAY	ssc04392:Hippo signaling pathway - multiple species	0.044950634
KEGG_PATHWAY	ssc05213:Endometrial cancer	0.045731972
KEGG_PATHWAY	ssc05205:Proteoglycans in cancer	0.049295022
KEGG_PATHWAY	ssc04660:T cell receptor signaling pathway	0.049386972
KEGG_PATHWAY	ssc04620:Toll-like receptor signaling pathway	0.049500223

cluster9

Category	Term	PValue
GOTERM_BP_DIRECT	GO:0032981~mitochondrial respiratory chain complex I assembly	3.76E-22
GOTERM_BP_DIRECT	GO:0006099~tricarboxylic acid cycle	6.15E-10
GOTERM_BP_DIRECT	GO:0006635~fatty acid beta-oxidation	1.25E-09
GOTERM_BP_DIRECT	GO:0006120~mitochondrial electron transport, NADH to ubiquinone	9.86E-09
GOTERM_BP_DIRECT	GO:0033539~fatty acid beta-oxidation using acyl-CoA dehydrogenase	2.05E-07
GOTERM_BP_DIRECT	GO:0015986~proton motive force-driven ATP synthesis	4.56E-07

GOTERM_BP_DIRECT	GO:0032496~response to lipopolysaccharide	3.31E-06
GOTERM_BP_DIRECT	GO:0010906~regulation of glucose metabolic process	5.26E-05
GOTERM_BP_DIRECT	GO:0120162~positive regulation of cold-induced thermogenesis	7.70E-05
GOTERM_BP_DIRECT	GO:0006122~mitochondrial electron transport, ubiquinol to cytochrome c	7.71E-05
GOTERM_BP_DIRECT	GO:0045766~positive regulation of angiogenesis	1.31E-04
GOTERM_BP_DIRECT	GO:0034551~mitochondrial respiratory chain complex III assembly	1.50E-04
GOTERM_BP_DIRECT	GO:0006086~acetyl-CoA biosynthetic process from pyruvate	1.85E-04
GOTERM_BP_DIRECT	GO:0042776~proton motive force-driven mitochondrial ATP synthesis	1.86E-04
GOTERM_BP_DIRECT	GO:0043123~positive regulation of canonical NF-kappaB signal transduction	2.12E-04
GOTERM_BP_DIRECT	GO:0006123~mitochondrial electron transport, cytochrome c to oxygen	2.14E-04
GOTERM_BP_DIRECT	GO:0010508~positive regulation of autophagy	4.06E-04
GOTERM_BP_DIRECT	GO:0006869~lipid transport	4.21E-04
GOTERM_BP_DIRECT	GO:0006979~response to oxidative stress	5.21E-04
GOTERM_BP_DIRECT	GO:0006631~fatty acid metabolic process	6.00E-04
GOTERM_BP_DIRECT	GO:0042407~cristae formation	6.60E-04
GOTERM_BP_DIRECT	GO:0009060~aerobic respiration	6.60E-04
GOTERM_BP_DIRECT	GO:0033617~mitochondrial cytochrome c oxidase assembly	8.71E-04
GOTERM_BP_DIRECT	GO:0006744~ubiquinone biosynthetic process	9.86E-04
GOTERM_BP_DIRECT	GO:1903589~positive regulation of blood vessel endothelial cell proliferation involved in sprouting angiogenesis	0.001136188
GOTERM_BP_DIRECT	GO:0033615~mitochondrial proton-transporting ATP synthase complex assembly	0.001136188
GOTERM_BP_DIRECT	GO:0008285~negative regulation of cell population proliferation	0.001299878
GOTERM_BP_DIRECT	GO:0050728~negative regulation of inflammatory response	0.001394393
GOTERM_BP_DIRECT	GO:0007005~mitochondrion organization	0.001395194
GOTERM_BP_DIRECT	GO:0016226~iron-sulfur cluster assembly	0.001538934
GOTERM_BP_DIRECT	GO:0045725~positive regulation of glycogen biosynthetic process	0.001539994
GOTERM_BP_DIRECT	GO:0045071~negative regulation of viral genome replication	0.001604244
GOTERM_BP_DIRECT	GO:0001525~angiogenesis	0.001689238
GOTERM_BP_DIRECT	GO:0007599~hemostasis	0.001875561
GOTERM_BP_DIRECT	GO:0006550~isoleucine catabolic process	0.001875561

GOTERM_BP_DIRECT	GO:0061028~establishment of endothelial barrier	0.001991508
GOTERM_BP_DIRECT	GO:0039536~negative regulation of RIG-I signaling pathway	0.002129711
GOTERM_BP_DIRECT	GO:0006103~2-oxoglutarate metabolic process	0.002339915
GOTERM_BP_DIRECT	GO:0003180~aortic valve morphogenesis	0.002622818
GOTERM_BP_DIRECT	GO:0005978~glycogen biosynthetic process	0.002717201
GOTERM_BP_DIRECT	GO:0001570~vasculogenesis	0.00300848
GOTERM_BP_DIRECT	GO:0051897~positive regulation of phosphatidylinositol 3-kinase/protein kinase B signal transduction	0.003144514
GOTERM_BP_DIRECT	GO:0045820~negative regulation of glycolytic process	0.003403925
GOTERM_BP_DIRECT	GO:0045647~negative regulation of erythrocyte differentiation	0.003593507
GOTERM_BP_DIRECT	GO:0043536~positive regulation of blood vessel endothelial cell migration	0.004213301
GOTERM_BP_DIRECT	GO:0001666~response to hypoxia	0.004367211
GOTERM_BP_DIRECT	GO:0033108~mitochondrial respiratory chain complex assembly	0.004411561
GOTERM_BP_DIRECT	GO:0031581~hemidesmosome assembly	0.004411561
GOTERM_BP_DIRECT	GO:0022904~respiratory electron transport chain	0.004737779
GOTERM_BP_DIRECT	GO:0006515~protein quality control for misfolded or incompletely synthesized proteins	0.004775657
GOTERM_BP_DIRECT	GO:0032543~mitochondrial translation	0.005229761
GOTERM_BP_DIRECT	GO:0072593~reactive oxygen species metabolic process	0.005239401
GOTERM_BP_DIRECT	GO:0051289~protein homotetramerization	0.005255145
GOTERM_BP_DIRECT	GO:0006094~gluconeogenesis	0.005255145
GOTERM_BP_DIRECT	GO:0045944~positive regulation of transcription by RNA polymerase II	0.006001945
GOTERM_BP_DIRECT	GO:0009725~response to hormone	0.00608377
GOTERM_BP_DIRECT	GO:0006006~glucose metabolic process	0.007928472
GOTERM_BP_DIRECT	GO:0042116~macrophage activation	0.00827508
GOTERM_BP_DIRECT	GO:0006520~amino acid metabolic process	0.00827508
GOTERM_BP_DIRECT	GO:1901098~positive regulation of autophagosome maturation	0.008303465
GOTERM_BP_DIRECT	GO:0006102~isocitrate metabolic process	0.008303465
GOTERM_BP_DIRECT	GO:0032471~negative regulation of endoplasmic reticulum calcium ion concentration	0.008303465
GOTERM_BP_DIRECT	GO:0050852~T cell receptor signaling pathway	0.008320111
GOTERM_BP_DIRECT	GO:0043410~positive regulation of MAPK cascade	0.009177186
GOTERM_BP_DIRECT	GO:0034097~response to cytokine	0.009446024
GOTERM_BP_DIRECT	GO:0003007~heart morphogenesis	0.009446024
GOTERM_BP_DIRECT	GO:0002931~response to ischemia	0.009575294
GOTERM_BP_DIRECT	GO:0035458~cellular response to interferon-beta	0.009575294
GOTERM_BP_DIRECT	GO:0050821~protein stabilization	0.01026031
GOTERM_BP_DIRECT	GO:0042593~glucose homeostasis	0.011150118

GOTERM_BP_DIRECT	GO:0006956~complement activation	0.011159837
GOTERM_BP_DIRECT	GO:0071456~cellular response to hypoxia	0.011311908
GOTERM_BP_DIRECT	GO:0008637~apoptotic mitochondrial changes	0.011642623
GOTERM_BP_DIRECT	GO:0030168~platelet activation	0.011770813
GOTERM_BP_DIRECT	GO:0045746~negative regulation of Notch signaling pathway	0.013362179
GOTERM_BP_DIRECT	GO:0035912~dorsal aorta morphogenesis	0.013678923
GOTERM_BP_DIRECT	GO:0071260~cellular response to mechanical stimulus	0.013919798
GOTERM_BP_DIRECT	GO:0051216~cartilage development	0.013919798
GOTERM_BP_DIRECT	GO:0006730~one-carbon metabolic process	0.014298051
GOTERM_BP_DIRECT	GO:0000422~autophagy of mitochondrion	0.014298051
GOTERM_BP_DIRECT	GO:0043065~positive regulation of apoptotic process	0.014857897
GOTERM_BP_DIRECT	GO:0030837~negative regulation of actin filament polymerization	0.01577716
GOTERM_BP_DIRECT	GO:0019216~regulation of lipid metabolic process	0.017179408
GOTERM_BP_DIRECT	GO:0008053~mitochondrial fusion	0.017692031
GOTERM_BP_DIRECT	GO:0003231~cardiac ventricle development	0.017875404
GOTERM_BP_DIRECT	GO:0019254~carnitine metabolic process, CoA-linked	0.017875404
GOTERM_BP_DIRECT	GO:0006096~glycolytic process	0.018322402
GOTERM_BP_DIRECT	GO:1905719~protein localization to perinuclear region of cytoplasm	0.02060845
GOTERM_BP_DIRECT	GO:0002467~germinal center formation	0.02060845
GOTERM_BP_DIRECT	GO:0034375~high-density lipoprotein particle remodeling	0.020726454
GOTERM_BP_DIRECT	GO:0048844~artery morphogenesis	0.020726454
GOTERM_BP_DIRECT	GO:0051145~smooth muscle cell differentiation	0.020726454
GOTERM_BP_DIRECT	GO:0006626~protein targeting to mitochondrion	0.020726454
GOTERM_BP_DIRECT	GO:0061436~establishment of skin barrier	0.021232659
GOTERM_BP_DIRECT	GO:0010875~positive regulation of cholesterol efflux	0.021740235
GOTERM_BP_DIRECT	GO:0009058~biosynthetic process	0.021740235
GOTERM_BP_DIRECT	GO:0010629~negative regulation of gene expression	0.023428363
GOTERM_BP_DIRECT	GO:0140374~antiviral innate immune response	0.023746981
GOTERM_BP_DIRECT	GO:0033077~T cell differentiation in thymus	0.024086307
GOTERM_BP_DIRECT	GO:0120163~negative regulation of cold-induced thermogenesis	0.024448649
GOTERM_BP_DIRECT	GO:0009968~negative regulation of signal transduction	0.024448649
GOTERM_BP_DIRECT	GO:0035556~intracellular signal transduction	0.025475592
GOTERM_BP_DIRECT	GO:0042632~cholesterol homeostasis	0.025858206
GOTERM_BP_DIRECT	GO:0030150~protein import into mitochondrial matrix	0.026345078
GOTERM_BP_DIRECT	GO:0070328~triglyceride homeostasis	0.026345078
GOTERM_BP_DIRECT	GO:0071360~cellular response to exogenous dsRNA	0.026526743
GOTERM_BP_DIRECT	GO:0006809~nitric oxide biosynthetic process	0.026526743
GOTERM_BP_DIRECT	GO:0007266~Rho protein signal transduction	0.02681375
GOTERM_BP_DIRECT	GO:0071356~cellular response to tumor necrosis factor	0.027854503
GOTERM_BP_DIRECT	GO:0030032~lamellipodium assembly	0.028148254

GOTERM_BP_DIRECT	GO:0014850~response to muscle activity	0.029115921
GOTERM_BP_DIRECT	GO:0060426~lung vasculature development	0.029115921
GOTERM_BP_DIRECT	GO:2000048~negative regulation of cell-cell adhesion mediated by cadherin	0.029115921
GOTERM_BP_DIRECT	GO:0070830~bicellular tight junction assembly	0.031132734
GOTERM_BP_DIRECT	GO:0045600~positive regulation of fat cell differentiation	0.031848698
GOTERM_BP_DIRECT	GO:0002040~sprouting angiogenesis	0.031848698
GOTERM_BP_DIRECT	GO:0051085~chaperone cofactor-dependent protein refolding	0.032636789
GOTERM_BP_DIRECT	GO:0001676~long-chain fatty acid metabolic process	0.033202935
GOTERM_BP_DIRECT	GO:0035924~cellular response to vascular endothelial growth factor stimulus	0.033202935
GOTERM_BP_DIRECT	GO:0060546~negative regulation of necroptotic process	0.033202935
GOTERM_BP_DIRECT	GO:0042060~wound healing	0.033749135
GOTERM_BP_DIRECT	GO:1902895~positive regulation of miRNA transcription	0.03381537
GOTERM_BP_DIRECT	GO:0051127~positive regulation of actin nucleation	0.033875247
GOTERM_BP_DIRECT	GO:0006533~aspartate catabolic process	0.033875247
GOTERM_BP_DIRECT	GO:0002317~plasma cell differentiation	0.033875247
GOTERM_BP_DIRECT	GO:0006104~succinyl-CoA metabolic process	0.033875247
GOTERM_BP_DIRECT	GO:0010510~regulation of acetyl-CoA biosynthetic process from pyruvate	0.033875247
GOTERM_BP_DIRECT	GO:0090324~negative regulation of oxidative phosphorylation	0.033875247
GOTERM_BP_DIRECT	GO:0019218~regulation of steroid metabolic process	0.033875247
GOTERM_BP_DIRECT	GO:0035344~hypoxanthine transport	0.033875247
GOTERM_BP_DIRECT	GO:0001974~blood vessel remodeling	0.037564896
GOTERM_BP_DIRECT	GO:0030042~actin filament depolymerization	0.039187584
GOTERM_BP_DIRECT	GO:0051561~positive regulation of mitochondrial calcium ion concentration	0.039187584
GOTERM_BP_DIRECT	GO:0061635~regulation of protein complex stability	0.039187584
GOTERM_BP_DIRECT	GO:0022900~electron transport chain	0.039187584
GOTERM_BP_DIRECT	GO:0043951~negative regulation of cAMP-mediated signaling	0.039187584
GOTERM_BP_DIRECT	GO:0035456~response to interferon-beta	0.039187584
GOTERM_BP_DIRECT	GO:0001938~positive regulation of endothelial cell proliferation	0.039995129
GOTERM_BP_DIRECT	GO:0055013~cardiac muscle cell development	0.040769045
GOTERM_BP_DIRECT	GO:0008154~actin polymerization or depolymerization	0.040769045
GOTERM_BP_DIRECT	GO:0006914~autophagy	0.041151853
GOTERM_BP_DIRECT	GO:0030308~negative regulation of cell growth	0.041625946
GOTERM_BP_DIRECT	GO:0006816~calcium ion transport	0.042943277
GOTERM_BP_DIRECT	GO:0019915~lipid storage	0.042943277
GOTERM_BP_DIRECT	GO:0030501~positive regulation of bone mineralization	0.048780293
GOTERM_BP_DIRECT	GO:0000902~cell morphogenesis	0.049077912

GOTERM_BP_DIRECT	GO:0043129~surfactant homeostasis	0.049228819
GOTERM_BP_DIRECT	GO:0097746~blood vessel diameter maintenance	0.049228819
GOTERM_CC_DIRECT	GO:0005739~mitochondrion	2.08E-57
GOTERM_CC_DIRECT	GO:0005759~mitochondrial matrix	4.16E-27
GOTERM_CC_DIRECT	GO:0005743~mitochondrial inner membrane	4.77E-23
GOTERM_CC_DIRECT	GO:0005829~cytosol	1.16E-08
GOTERM_CC_DIRECT	GO:0005751~mitochondrial respiratory chain complex IV	3.24E-08
GOTERM_CC_DIRECT	GO:0005750~mitochondrial respiratory chain complex III	1.49E-07
GOTERM_CC_DIRECT	GO:0005654~nucleoplasm	4.48E-07
GOTERM_CC_DIRECT	GO:0042645~mitochondrial nucleoid	1.34E-06
GOTERM_CC_DIRECT	GO:0005741~mitochondrial outer membrane	5.44E-06
GOTERM_CC_DIRECT	GO:0005737~cytoplasm	3.96E-05
GOTERM_CC_DIRECT	GO:0005758~mitochondrial intermembrane space	2.70E-04
GOTERM_CC_DIRECT	GO:0031966~mitochondrial membrane	3.25E-04
GOTERM_CC_DIRECT	GO:0000276~mitochondrial proton-transporting ATP synthase complex, coupling factor F(o)	3.29E-04
GOTERM_CC_DIRECT	GO:0005753~mitochondrial proton-transporting ATP synthase complex	4.80E-04
GOTERM_CC_DIRECT	GO:0005763~mitochondrial small ribosomal subunit	0.002002906
GOTERM_CC_DIRECT	GO:0030056~hemidesmosome	0.003852136
GOTERM_CC_DIRECT	GO:0062023~collagen-containing extracellular matrix	0.004772642
GOTERM_CC_DIRECT	GO:0090575~RNA polymerase II transcription regulator complex	0.0068217
GOTERM_CC_DIRECT	GO:0005925~focal adhesion	0.007694418
GOTERM_CC_DIRECT	GO:0042383~sarcolemma	0.00832708
GOTERM_CC_DIRECT	GO:0033017~sarcoplasmic reticulum membrane	0.009186916
GOTERM_CC_DIRECT	GO:0061617~MICOS complex	0.009880058
GOTERM_CC_DIRECT	GO:0005777~peroxisome	0.010225561
GOTERM_CC_DIRECT	GO:0005757~mitochondrial permeability transition pore complex	0.012012263
GOTERM_CC_DIRECT	GO:0045261~proton-transporting ATP synthase complex, catalytic core F(1)	0.012012263
GOTERM_CC_DIRECT	GO:0045252~oxoglutarate dehydrogenase complex	0.012012263
GOTERM_CC_DIRECT	GO:0110142~ubiquinone biosynthesis complex	0.012012263
GOTERM_CC_DIRECT	GO:0042612~MHC class I protein complex	0.014652944
GOTERM_CC_DIRECT	GO:0032991~protein-containing complex	0.015092404
GOTERM_CC_DIRECT	GO:0045254~pyruvate dehydrogenase complex	0.016308962
GOTERM_CC_DIRECT	GO:0030027~lamellipodium	0.016396133
GOTERM_CC_DIRECT	GO:0005604~basement membrane	0.016810336
GOTERM_CC_DIRECT	GO:0035770~ribonucleoprotein granule	0.017692382
GOTERM_CC_DIRECT	GO:0030018~Z disc	0.020927585
GOTERM_CC_DIRECT	GO:0030016~myofibril	0.022912273
GOTERM_CC_DIRECT	GO:0031314~extrinsic component of mitochondrial inner membrane	0.025711134

GOTERM_CC_DIRECT	GO:0031410~cytoplasmic vesicle	0.026835447
GOTERM_CC_DIRECT	GO:0008076~voltage-gated potassium channel complex	0.02850885
GOTERM_CC_DIRECT	GO:0005776~autophagosome	0.029318636
GOTERM_CC_DIRECT	GO:0000275~mitochondrial proton-transporting ATP synthase complex, catalytic sector F(1)	0.030984902
GOTERM_CC_DIRECT	GO:1902554~serine/threonine protein kinase complex	0.030984902
GOTERM_CC_DIRECT	GO:0033093~Weibel-Palade body	0.030984902
GOTERM_CC_DIRECT	GO:0005744~TIM23 mitochondrial import inner membrane translocase complex	0.034700068
GOTERM_CC_DIRECT	GO:0045121~membrane raft	0.034716992
GOTERM_CC_DIRECT	GO:0005911~cell-cell junction	0.036738237
GOTERM_CC_DIRECT	GO:0005811~lipid droplet	0.038454194
GOTERM_CC_DIRECT	GO:0030175~filopodium	0.040466538
GOTERM_CC_DIRECT	GO:0048471~perinuclear region of cytoplasm	0.04073569
GOTERM_CC_DIRECT	GO:0005901~caveola	0.042766709
GOTERM_CC_DIRECT	GO:0005912~adherens junction	0.047367984
GOTERM_CC_DIRECT	GO:0098553~lumenal side of endoplasmic reticulum membrane	0.049294588
GOTERM_MF_DIRECT	GO:0050660~flavin adenine dinucleotide binding	8.55E-07
GOTERM_MF_DIRECT	GO:0042803~protein homodimerization activity	1.48E-06
GOTERM_MF_DIRECT	GO:0009055~electron transfer activity	1.12E-05
GOTERM_MF_DIRECT	GO:0051539~4 iron, 4 sulfur cluster binding	1.57E-05
GOTERM_MF_DIRECT	GO:0046933~proton-transporting ATP synthase activity, rotational mechanism	1.65E-05
GOTERM_MF_DIRECT	GO:0015078~proton transmembrane transporter activity	4.00E-05
GOTERM_MF_DIRECT	GO:0016491~oxidoreductase activity	8.98E-05
GOTERM_MF_DIRECT	GO:0008137~NADH dehydrogenase (ubiquinone) activity	1.47E-04
GOTERM_MF_DIRECT	GO:0034604~pyruvate dehydrogenase (NAD+) activity	1.59E-04
GOTERM_MF_DIRECT	GO:0050661~NADP binding	2.83E-04
GOTERM_MF_DIRECT	GO:0042802~identical protein binding	5.62E-04
GOTERM_MF_DIRECT	GO:0031625~ubiquitin protein ligase binding	6.16E-04
GOTERM_MF_DIRECT	GO:0046872~metal ion binding	8.67E-04
GOTERM_MF_DIRECT	GO:0003995~acyl-CoA dehydrogenase activity	9.84E-04
GOTERM_MF_DIRECT	GO:0051287~NAD binding	0.001514842
GOTERM_MF_DIRECT	GO:0004300~enoyl-CoA hydratase activity	0.001848197
GOTERM_MF_DIRECT	GO:0071949~FAD binding	0.002105725
GOTERM_MF_DIRECT	GO:0004740~pyruvate dehydrogenase (acetyl-transferring) kinase activity	0.003955638
GOTERM_MF_DIRECT	GO:0050833~pyruvate transmembrane transporter activity	0.003955638
GOTERM_MF_DIRECT	GO:0016614~oxidoreductase activity, acting on CH-OH group of donors	0.004897051
GOTERM_MF_DIRECT	GO:0001228~DNA-binding transcription activator activity, RNA polymerase II-specific	0.006249583
GOTERM_MF_DIRECT	GO:0051087~protein-folding chaperone binding	0.006585629

GOTERM_MF_DIRECT	GO:0061629~RNA polymerase II-specific DNA-binding transcription factor binding	0.006976942
GOTERM_MF_DIRECT	GO:0005355~glucose transmembrane transporter activity	0.007234053
GOTERM_MF_DIRECT	GO:0008270~zinc ion binding	0.00797477
GOTERM_MF_DIRECT	GO:0000287~magnesium ion binding	0.008890134
GOTERM_MF_DIRECT	GO:0031072~heat shock protein binding	0.009672748
GOTERM_MF_DIRECT	GO:0005044~scavenger receptor activity	0.009852122
GOTERM_MF_DIRECT	GO:0048039~ubiquinone binding	0.012321776
GOTERM_MF_DIRECT	GO:0008140~cAMP response element binding protein binding	0.012321776
GOTERM_MF_DIRECT	GO:0030170~pyridoxal phosphate binding	0.014781872
GOTERM_MF_DIRECT	GO:0005319~lipid transporter activity	0.015203695
GOTERM_MF_DIRECT	GO:0005229~intracellularly calcium-gated chloride channel activity	0.015203695
GOTERM_MF_DIRECT	GO:0015207~adenine transmembrane transporter activity	0.016604781
GOTERM_MF_DIRECT	GO:0047101~branched-chain alpha-keto acid dehydrogenase activity	0.016604781
GOTERM_MF_DIRECT	GO:0004449~isocitrate dehydrogenase (NAD+) activity	0.016604781
GOTERM_MF_DIRECT	GO:0008138~protein tyrosine/serine/threonine phosphatase activity	0.017233829
GOTERM_MF_DIRECT	GO:0005518~collagen binding	0.018015754
GOTERM_MF_DIRECT	GO:0031404~chloride ion binding	0.018605923
GOTERM_MF_DIRECT	GO:0051537~2 iron, 2 sulfur cluster binding	0.023855759
GOTERM_MF_DIRECT	GO:0004722~protein serine/threonine phosphatase activity	0.024701907
GOTERM_MF_DIRECT	GO:0005524~ATP binding	0.025364529
GOTERM_MF_DIRECT	GO:0005515~protein binding	0.026020341
GOTERM_MF_DIRECT	GO:0030247~polysaccharide binding	0.026345796
GOTERM_MF_DIRECT	GO:2001069~glycogen binding	0.026345796
GOTERM_MF_DIRECT	GO:0004879~nuclear receptor activity	0.027889969
GOTERM_MF_DIRECT	GO:0004806~triglyceride lipase activity	0.029372664
GOTERM_MF_DIRECT	GO:0008177~succinate dehydrogenase (quinone) activity	0.031531752
GOTERM_MF_DIRECT	GO:0004095~carnitine O-palmitoyltransferase activity	0.031531752
GOTERM_MF_DIRECT	GO:0016401~palmitoyl-CoA oxidase activity	0.031531752
GOTERM_MF_DIRECT	GO:0015208~guanine transmembrane transporter activity	0.031531752
GOTERM_MF_DIRECT	GO:0004466~long-chain fatty acyl-CoA dehydrogenase activity	0.031531752
GOTERM_MF_DIRECT	GO:0035877~death effector domain binding	0.031531752
GOTERM_MF_DIRECT	GO:0102545~phosphatidyl phospholipase B activity	0.031531752
GOTERM_MF_DIRECT	GO:0050840~extracellular matrix binding	0.031992811
GOTERM_MF_DIRECT	GO:0000166~nucleotide binding	0.034335415
GOTERM_MF_DIRECT	GO:0048038~quinone binding	0.035538115
GOTERM_MF_DIRECT	GO:0004176~ATP-dependent peptidase activity	0.035538115
GOTERM_MF_DIRECT	GO:0005509~calcium ion binding	0.039933304

GOTERM_MF_DIRECT	GO:0051020~GTPase binding	0.044189684
GOTERM_MF_DIRECT	GO:0070700~BMP receptor binding	0.046152139
GOTERM_MF_DIRECT	GO:0005080~protein kinase C binding	0.047252827
GOTERM_MF_DIRECT	GO:0141039~phosphatidylinositol 3-kinase inhibitor activity	0.049915827
GOTERM_MF_DIRECT	GO:0003954~NADH dehydrogenase activity	0.049915827
GOTERM_MF_DIRECT	GO:0003985~acetyl-CoA C-acetyltransferase activity	0.049915827
KEGG_PATHWAY	ssc05415:Diabetic cardiomyopathy	2.63E-45
KEGG_PATHWAY	ssc04714:Thermogenesis	1.42E-41
KEGG_PATHWAY	ssc00190:Oxidative phosphorylation	1.21E-40
KEGG_PATHWAY	ssc04932:Non-alcoholic fatty liver disease	2.60E-35
KEGG_PATHWAY	ssc05208:Chemical carcinogenesis - reactive oxygen species	1.39E-34
KEGG_PATHWAY	ssc01100:Metabolic pathways	6.91E-28
KEGG_PATHWAY	ssc05020:Prion disease	8.36E-26
KEGG_PATHWAY	ssc05012:Parkinson disease	3.34E-24
KEGG_PATHWAY	ssc05016:Huntington disease	9.35E-24
KEGG_PATHWAY	ssc05010:Alzheimer disease	9.49E-24
KEGG_PATHWAY	ssc05022:Pathways of neurodegeneration - multiple diseases	6.86E-22
KEGG_PATHWAY	ssc05014:Amyotrophic lateral sclerosis	3.42E-20
KEGG_PATHWAY	ssc01200:Carbon metabolism	2.09E-16
KEGG_PATHWAY	ssc04723:Retrograde endocannabinoid signaling	1.28E-15
KEGG_PATHWAY	ssc00020:Citrate cycle (TCA cycle)	5.33E-14
KEGG_PATHWAY	ssc01210:2-Oxocarboxylic acid metabolism	3.17E-13
KEGG_PATHWAY	ssc00280:Valine, leucine and isoleucine degradation	6.28E-10
KEGG_PATHWAY	ssc00071:Fatty acid degradation	5.36E-09
KEGG_PATHWAY	ssc00640:Propanoate metabolism	1.57E-08
KEGG_PATHWAY	ssc00785:Lipoic acid metabolism	3.76E-08
KEGG_PATHWAY	ssc04260:Cardiac muscle contraction	9.27E-07
KEGG_PATHWAY	ssc03320:PPAR signaling pathway	7.84E-06
KEGG_PATHWAY	ssc04022:cGMP-PKG signaling pathway	1.40E-05
KEGG_PATHWAY	ssc01212:Fatty acid metabolism	1.85E-05
KEGG_PATHWAY	ssc04931:Insulin resistance	2.01E-05
KEGG_PATHWAY	ssc01230:Biosynthesis of amino acids	1.15E-04
KEGG_PATHWAY	ssc04152:AMPK signaling pathway	1.17E-04
KEGG_PATHWAY	ssc04920:Adipocytokine signaling pathway	1.37E-04
KEGG_PATHWAY	ssc04211:Longevity regulating pathway	1.95E-04
KEGG_PATHWAY	ssc00630:Glyoxylate and dicarboxylate metabolism	1.97E-04
KEGG_PATHWAY	ssc04922:Glucagon signaling pathway	3.86E-04
KEGG_PATHWAY	ssc04146:Peroxisome	0.001205804
KEGG_PATHWAY	ssc04213:Longevity regulating pathway - multiple species	0.00167831
KEGG_PATHWAY	ssc04068:FoxO signaling pathway	0.00185121
KEGG_PATHWAY	ssc04066:HIF-1 signaling pathway	0.0054502

KEGG_PATHWAY	ssc04137:Mitophagy - animal	0.006155567
KEGG_PATHWAY	ssc04820:Cytoskeleton in muscle cells	0.006163859
KEGG_PATHWAY	ssc05167:Kaposi sarcoma-associated herpesvirus infection	0.006240228
KEGG_PATHWAY	ssc04218:Cellular senescence	0.007384607
KEGG_PATHWAY	ssc04926:Relaxin signaling pathway	0.008001285
KEGG_PATHWAY	ssc00620:Pyruvate metabolism	0.010578366
KEGG_PATHWAY	ssc05166:Human T-cell leukemia virus 1 infection	0.012447887
KEGG_PATHWAY	ssc05163:Human cytomegalovirus infection	0.014817615
KEGG_PATHWAY	ssc04371:Apelin signaling pathway	0.016337614
KEGG_PATHWAY	ssc04140:Autophagy - animal	0.016365868
KEGG_PATHWAY	ssc04621:NOD-like receptor signaling pathway	0.017336058
KEGG_PATHWAY	ssc05200:Pathways in cancer	0.017598314
KEGG_PATHWAY	ssc04923:Regulation of lipolysis in adipocytes	0.021635747
KEGG_PATHWAY	ssc04148:Efferocytosis	0.022899141
KEGG_PATHWAY	ssc01240:Biosynthesis of cofactors	0.023285887
KEGG_PATHWAY	ssc00740:Riboflavin metabolism	0.026560292
KEGG_PATHWAY	ssc00590:Arachidonic acid metabolism	0.02819477
KEGG_PATHWAY	ssc04925:Aldosterone synthesis and secretion	0.030217092
KEGG_PATHWAY	ssc04919:Thyroid hormone signaling pathway	0.031031352
KEGG_PATHWAY	ssc04151:PI3K-Akt signaling pathway	0.032120604
KEGG_PATHWAY	ssc05134:Legionellosis	0.033505098
KEGG_PATHWAY	ssc04261:Adrenergic signaling in cardiomyocytes	0.034379168
KEGG_PATHWAY	ssc04936:Alcoholic liver disease	0.03491928
KEGG_PATHWAY	ssc04725:Cholinergic synapse	0.035826588
KEGG_PATHWAY	ssc04964:Proximal tubule bicarbonate reclamation	0.037705625
KEGG_PATHWAY	ssc04960:Aldosterone-regulated sodium reabsorption	0.038334171
KEGG_PATHWAY	ssc00010:Glycolysis / Gluconeogenesis	0.04088772
KEGG_PATHWAY	ssc04668:TNF signaling pathway	0.041202438
KEGG_PATHWAY	ssc04610:Complement and coagulation cascades	0.043778986
KEGG_PATHWAY	ssc00310:Lysine degradation	0.044966895
