# Supplementary material for: Dynamic changes in chromatin accessibility and gene expression involved in fetal myogenesis of Min pigs
Source: Anim Biosci. 2025 May 12;38(11):2525–36. doi: 10.5713/ab.25.0034 (PMC12580940; doi:10.5713/ab.25.0034)
Supplement: Supplementary file 8 [file ab-25-0034-supplementary-8.pdf]

Supplement 8. GO and KEGG enrichment analysis of overlapping genes.

E45＞E100

Category	Term	PValue
GOTERM_BP_DIRECT	GO:0010976~positive regulation of neuron projection development	1.37E-05
GOTERM_BP_DIRECT	GO:0051965~positive regulation of synapse assembly	4.39E-05
GOTERM_BP_DIRECT	GO:0007157~heterophilic cell-cell adhesion via plasma membrane cell adhesion molecules	7.45E-04
GOTERM_BP_DIRECT	GO:0007156~homophilic cell adhesion via plasma membrane adhesion molecules	0.001910959
GOTERM_BP_DIRECT	GO:0048013~ephrin receptor signaling pathway	0.002018842
GOTERM_BP_DIRECT	GO:0030513~positive regulation of BMP signaling pathway	0.002938525
GOTERM_BP_DIRECT	GO:0060563~neuroepithelial cell differentiation	0.003253739
GOTERM_BP_DIRECT	GO:0007043~cell-cell junction assembly	0.003480425
GOTERM_BP_DIRECT	GO:0098609~cell-cell adhesion	0.006649969
GOTERM_BP_DIRECT	GO:0030336~negative regulation of cell migration	0.006821199
GOTERM_BP_DIRECT	GO:0007267~cell-cell signaling	0.007081289
GOTERM_BP_DIRECT	GO:0007275~multicellular organism development	0.007696213
GOTERM_BP_DIRECT	GO:0007205~protein kinase C-activating G-protein coupled receptor signaling pathway	0.008383748
GOTERM_BP_DIRECT	GO:0044331~cell-cell adhesion mediated by cadherin	0.014259245
GOTERM_BP_DIRECT	GO:0051966~regulation of synaptic transmission, glutamatergic	0.014259245
GOTERM_BP_DIRECT	GO:0099560~synaptic membrane adhesion	0.015596191
GOTERM_BP_DIRECT	GO:0048856~anatomical structure development	0.016951751
GOTERM_BP_DIRECT	GO:0045197~establishment or maintenance of epithelial cell apical/basal polarity	0.019912526
GOTERM_BP_DIRECT	GO:0071880~adenylate cyclase-activating adrenergic receptor signaling pathway	0.019912526
GOTERM_BP_DIRECT	GO:0050804~modulation of synaptic transmission	0.024210177
GOTERM_BP_DIRECT	GO:0070507~regulation of microtubule cytoskeleton organization	0.024666717
GOTERM_BP_DIRECT	GO:0007268~chemical synaptic transmission	0.025274082
GOTERM_BP_DIRECT	GO:0009887~animal organ morphogenesis	0.02601742
GOTERM_BP_DIRECT	GO:0016339~calcium-dependent cell-cell adhesion via plasma membrane cell adhesion molecules	0.026344337
GOTERM_BP_DIRECT	GO:0034332~adherens junction organization	0.029833412
GOTERM_BP_DIRECT	GO:0007411~axon guidance	0.037388405
GOTERM_BP_DIRECT	GO:0003151~outflow tract morphogenesis	0.039295564
GOTERM_BP_DIRECT	GO:0071805~potassium ion transmembrane transport	0.04062863
GOTERM_BP_DIRECT	GO:0060513~prostatic bud formation	0.042886344
GOTERM_BP_DIRECT	GO:0007154~cell communication	0.04335828
GOTERM_BP_DIRECT	GO:0006644~phospholipid metabolic process	0.049729565

GOTERM_CC_DIRECT	GO:0005886~plasma membrane	1.31E-06
GOTERM_CC_DIRECT	GO:0005912~adherens junction	1.22E-05
GOTERM_CC_DIRECT	GO:0098978~glutamatergic synapse	1.96E-05
GOTERM_CC_DIRECT	GO:0098839~postsynaptic density membrane	1.51E-04
GOTERM_CC_DIRECT	GO:0043005~neuron projection	8.33E-04
GOTERM_CC_DIRECT	GO:0098982~GABA-ergic synapse	9.14E-04
GOTERM_CC_DIRECT	GO:0009986~cell surface	0.002446858
GOTERM_CC_DIRECT	GO:0030425~dendrite	0.002922431
GOTERM_CC_DIRECT	GO:0016342~catenin complex	0.003117232
GOTERM_CC_DIRECT	GO:0005769~early endosome	0.006481122
GOTERM_CC_DIRECT	GO:0048787~presynaptic active zone membrane	0.007351742
GOTERM_CC_DIRECT	GO:0030424~axon	0.009050891
GOTERM_CC_DIRECT	GO:0005922~connexin complex	0.013706915
GOTERM_CC_DIRECT	GO:0016323~basolateral plasma membrane	0.01477384
GOTERM_CC_DIRECT	GO:0042734~presynaptic membrane	0.019548152
GOTERM_CC_DIRECT	GO:0045202~synapse	0.020244666
GOTERM_CC_DIRECT	GO:0015629~actin cytoskeleton	0.020308621
GOTERM_CC_DIRECT	GO:0098793~presynapse	0.021903663
GOTERM_CC_DIRECT	GO:0014069~postsynaptic density	0.024156568
GOTERM_CC_DIRECT	GO:0045211~postsynaptic membrane	0.024740905
GOTERM_CC_DIRECT	GO:0098685~Schaffer collateral - CA1 synapse	0.026789485
GOTERM_CC_DIRECT	GO:0098552~side of membrane	0.036504092
GOTERM_CC_DIRECT	GO:0005615~extracellular space	0.038214876
GOTERM_CC_DIRECT	GO:0036477~somatodendritic compartment	0.040096642
GOTERM_CC_DIRECT	GO:0001917~photoreceptor inner segment	0.045968624
GOTERM_CC_DIRECT	GO:0001750~photoreceptor outer segment	0.049980074
GOTERM_MF_DIRECT	GO:0005509~calcium ion binding	6.23E-05
GOTERM_MF_DIRECT	GO:0051015~actin filament binding	3.17E-04
GOTERM_MF_DIRECT	GO:0045296~cadherin binding	0.002816914
GOTERM_MF_DIRECT	GO:0042803~protein homodimerization activity	0.00810529
GOTERM_MF_DIRECT	GO:0004993~G-protein coupled serotonin receptor activity	0.018788499
GOTERM_MF_DIRECT	GO:0008013~beta-catenin binding	0.024065597
GOTERM_MF_DIRECT	GO:0046923~ER retention sequence binding	0.032133422
GOTERM_MF_DIRECT	GO:0005201~extracellular matrix structural constituent	0.032804497
GOTERM_MF_DIRECT	GO:0030020~extracellular matrix structural constituent conferring tensile strength	0.032804497
GOTERM_MF_DIRECT	GO:0003924~GTPase activity	0.038189528
GOTERM_MF_DIRECT	GO:0046790~virion binding	0.040005165
KEGG_PATHWAY	ssc04810:Regulation of actin cytoskeleton	0.002278815
KEGG_PATHWAY	ssc04072:Phospholipase D signaling pathway	0.006800345
KEGG_PATHWAY	ssc05100:Bacterial invasion of epithelial cells	0.023794895
KEGG_PATHWAY	ssc04015:Rap1 signaling pathway	0.027982083
KEGG_PATHWAY	ssc05226:Gastric cancer	0.031575324
KEGG_PATHWAY	ssc04514:Cell adhesion molecules	0.035027458

KEGG_PATHWAY	ssc04390:Hippo signaling pathway	0.035743287
KEGG_PATHWAY	ssc04014:Ras signaling pathway	0.040802705
KEGG_PATHWAY	ssc04144:Endocytosis	0.043344539

E70＞E45

Category	Term	PValue
GOTERM_BP_DIRECT	GO:0042771~intrinsic apoptotic signaling pathway in response to DNA damage by p53 class mediator	0.043369932
GOTERM_CC_DIRECT	GO:0009898~cytoplasmic side of plasma membrane	0.004421676
GOTERM_MF_DIRECT	GO:0030280~structural constituent of epidermis	0.043448797

E70＞E100

Category	Term	PValue
GOTERM_BP_DIRECT	GO:0007043~cell-cell junction assembly	0.002736449
GOTERM_BP_DIRECT	GO:0060019~radial glial cell differentiation	0.015449799
GOTERM_BP_DIRECT	GO:0021819~layer formation in cerebral cortex	0.028506826
GOTERM_BP_DIRECT	GO:0048514~blood vessel morphogenesis	0.0306665
GOTERM_BP_DIRECT	GO:0007420~brain development	0.039154816
GOTERM_BP_DIRECT	GO:0030198~extracellular matrix organization	0.039154816
GOTERM_BP_DIRECT	GO:0007156~homophilic cell adhesion via plasma membrane adhesion molecules	0.04273784
GOTERM_BP_DIRECT	GO:0044331~cell-cell adhesion mediated by cadherin	0.045653533
GOTERM_CC_DIRECT	GO:0030425~dendrite	0.011945913
GOTERM_CC_DIRECT	GO:0005912~adherens junction	0.029889764
GOTERM_CC_DIRECT	GO:0099634~postsynaptic specialization membrane	0.040451647
GOTERM_CC_DIRECT	GO:0031012~extracellular matrix	0.048971629
GOTERM_MF_DIRECT	GO:0005201~extracellular matrix structural constituent	5.45E-05
GOTERM_MF_DIRECT	GO:0045296~cadherin binding	0.010126982
GOTERM_MF_DIRECT	GO:0035255~ionotropic glutamate receptor binding	0.027119799
KEGG_PATHWAY	ssc04514:Cell adhesion molecules	0.00364282
KEGG_PATHWAY	ssc04974:Protein digestion and absorption	0.01903338

E100＞E45

Category	Term	PValue
GOTERM_BP_DIRECT	GO:0007155~cell adhesion	3.71E-05
GOTERM_BP_DIRECT	GO:0001516~prostaglandin biosynthetic process	6.09E-05
GOTERM_BP_DIRECT	GO:0010628~positive regulation of gene expression	1.31E-04
GOTERM_BP_DIRECT	GO:0050731~positive regulation of peptidyl-tyrosine phosphorylation	2.75E-04
GOTERM_BP_DIRECT	GO:0032922~circadian regulation of gene expression	5.64E-04
GOTERM_BP_DIRECT	GO:0008285~negative regulation of cell proliferation	5.83E-04

GOTERM_BP_DIRECT	GO:0070588~calcium ion transmembrane transport	6.27E-04
GOTERM_BP_DIRECT	GO:0032496~response to lipopolysaccharide	7.68E-04
GOTERM_BP_DIRECT	GO:0045214~sarcomere organization	7.92E-04
GOTERM_BP_DIRECT	GO:0001525~angiogenesis	8.07E-04
GOTERM_BP_DIRECT	GO:0034614~cellular response to reactive oxygen species	0.001630196
GOTERM_BP_DIRECT	GO:0006357~regulation of transcription from RNA polymerase II promoter	0.001659507
GOTERM_BP_DIRECT	GO:0045840~positive regulation of mitotic nuclear division	0.002294086
GOTERM_BP_DIRECT	GO:0030335~positive regulation of cell migration	0.002980198
GOTERM_BP_DIRECT	GO:0006631~fatty acid metabolic process	0.00310238
GOTERM_BP_DIRECT	GO:0007599~hemostasis	0.00357936
GOTERM_BP_DIRECT	GO:0045821~positive regulation of glycolytic process	0.00362562
GOTERM_BP_DIRECT	GO:0006635~fatty acid beta-oxidation	0.005008983
GOTERM_BP_DIRECT	GO:0030032~lamellipodium assembly	0.005471879
GOTERM_BP_DIRECT	GO:0006885~regulation of pH	0.005559001
GOTERM_BP_DIRECT	GO:0072011~glomerular endothelium development	0.005867548
GOTERM_BP_DIRECT	GO:0072659~protein localization to plasma membrane	0.006054946
GOTERM_BP_DIRECT	GO:0070059~intrinsic apoptotic signaling pathway in response to endoplasmic reticulum stress	0.0061918
GOTERM_BP_DIRECT	GO:0030036~actin cytoskeleton organization	0.006799435
GOTERM_BP_DIRECT	GO:0000902~cell morphogenesis	0.007125623
GOTERM_BP_DIRECT	GO:0006470~protein dephosphorylation	0.007627177
GOTERM_BP_DIRECT	GO:0055013~cardiac muscle cell development	0.008007652
GOTERM_BP_DIRECT	GO:0045944~positive regulation of transcription from RNA polymerase II promoter	0.008010867
GOTERM_BP_DIRECT	GO:0045893~positive regulation of transcription, DNA-templated	0.008245295
GOTERM_BP_DIRECT	GO:0007275~multicellular organism development	0.008381351
GOTERM_BP_DIRECT	GO:0006198~cAMP catabolic process	0.008656961
GOTERM_BP_DIRECT	GO:0000122~negative regulation of transcription from RNA polymerase II promoter	0.009069158
GOTERM_BP_DIRECT	GO:0051091~positive regulation of sequence-specific DNA binding transcription factor activity	0.009434257
GOTERM_BP_DIRECT	GO:0031589~cell-substrate adhesion	0.009434257
GOTERM_BP_DIRECT	GO:0005978~glycogen biosynthetic process	0.009434257
GOTERM_BP_DIRECT	GO:0051897~positive regulation of protein kinase B signaling	0.009563941
GOTERM_BP_DIRECT	GO:0086091~regulation of heart rate by cardiac conduction	0.00969368
GOTERM_BP_DIRECT	GO:0006936~muscle contraction	0.00969368
GOTERM_BP_DIRECT	GO:0045785~positive regulation of cell adhesion	0.00969368
GOTERM_BP_DIRECT	GO:0016311~dephosphorylation	0.009772672
GOTERM_BP_DIRECT	GO:0043066~negative regulation of apoptotic process	0.009875867

GOTERM_BP_DIRECT	GO:0042127~regulation of cell proliferation	0.010578467
GOTERM_BP_DIRECT	GO:0043065~positive regulation of apoptotic process	0.010584421
GOTERM_BP_DIRECT	GO:0032755~positive regulation of interleukin-6 production	0.011551506
GOTERM_BP_DIRECT	GO:0040011~locomotion	0.011921371
GOTERM_BP_DIRECT	GO:0032471~negative regulation of endoplasmic reticulum calcium ion concentration	0.011921371
GOTERM_BP_DIRECT	GO:0055119~relaxation of cardiac muscle	0.011921371
GOTERM_BP_DIRECT	GO:0045165~cell fate commitment	0.012549017
GOTERM_BP_DIRECT	GO:0045600~positive regulation of fat cell differentiation	0.014278256
GOTERM_BP_DIRECT	GO:0008217~regulation of blood pressure	0.015605425
GOTERM_BP_DIRECT	GO:0043117~positive regulation of vascular permeability	0.01563554
GOTERM_BP_DIRECT	GO:2001214~positive regulation of vasculogenesis	0.01563554
GOTERM_BP_DIRECT	GO:0005979~regulation of glycogen biosynthetic process	0.01563554
GOTERM_BP_DIRECT	GO:0019227~neuronal action potential propagation	0.01563554
GOTERM_BP_DIRECT	GO:0038134~ERBB2-EGFR signaling pathway	0.01563554
GOTERM_BP_DIRECT	GO:0048661~positive regulation of smooth muscle cell proliferation	0.016547401
GOTERM_BP_DIRECT	GO:0071456~cellular response to hypoxia	0.017682626
GOTERM_BP_DIRECT	GO:0007165~signal transduction	0.01901569
GOTERM_BP_DIRECT	GO:0071805~potassium ion transmembrane transport	0.021296525
GOTERM_BP_DIRECT	GO:1990573~potassium ion import across plasma membrane	0.021676125
GOTERM_BP_DIRECT	GO:0006941~striated muscle contraction	0.024316947
GOTERM_BP_DIRECT	GO:0043951~negative regulation of cAMP-mediated signaling	0.024316947
GOTERM_BP_DIRECT	GO:0072112~glomerular visceral epithelial cell differentiation	0.024316947
GOTERM_BP_DIRECT	GO:0006366~transcription from RNA polymerase II promoter	0.024769842
GOTERM_BP_DIRECT	GO:0007417~central nervous system development	0.025907682
GOTERM_BP_DIRECT	GO:0007200~phospholipase C-activating G-protein coupled receptor signaling pathway	0.02694875
GOTERM_BP_DIRECT	GO:0060070~canonical Wnt signaling pathway	0.027156256
GOTERM_BP_DIRECT	GO:0010629~negative regulation of gene expression	0.028604658
GOTERM_BP_DIRECT	GO:0007162~negative regulation of cell adhesion	0.02869573
GOTERM_BP_DIRECT	GO:0015693~magnesium ion transport	0.029238365
GOTERM_BP_DIRECT	GO:0008637~apoptotic mitochondrial changes	0.03451784
GOTERM_BP_DIRECT	GO:0002437~inflammatory response to antigenic stimulus	0.03451784
GOTERM_BP_DIRECT	GO:0002040~sprouting angiogenesis	0.034572405
GOTERM_BP_DIRECT	GO:0048008~platelet-derived growth factor receptor signaling pathway	0.034572405
GOTERM_BP_DIRECT	GO:0006813~potassium ion transport	0.035368252
GOTERM_BP_DIRECT	GO:0006816~calcium ion transport	0.035368252

GOTERM_BP_DIRECT	GO:0046326~positive regulation of glucose import	0.037725806
GOTERM_BP_DIRECT	GO:0045746~negative regulation of Notch signaling pathway	0.037725806
GOTERM_BP_DIRECT	GO:0014898~cardiac muscle hypertrophy in response to stress	0.040134607
GOTERM_BP_DIRECT	GO:0007173~epidermal growth factor receptor signaling pathway	0.044456012
GOTERM_BP_DIRECT	GO:0060021~palate development	0.04500044
GOTERM_BP_DIRECT	GO:0006809~nitric oxide biosynthetic process	0.04606871
GOTERM_BP_DIRECT	GO:0019229~regulation of vasoconstriction	0.04606871
GOTERM_BP_DIRECT	GO:0046579~positive regulation of Ras protein signal transduction	0.04606871
GOTERM_BP_DIRECT	GO:0001974~blood vessel remodeling	0.048029511
GOTERM_BP_DIRECT	GO:0032743~positive regulation of interleukin-2 production	0.048029511
GOTERM_BP_DIRECT	GO:0006874~cellular calcium ion homeostasis	0.04901446
GOTERM_BP_DIRECT	GO:0072126~positive regulation of glomerular mesangial cell proliferation	0.049102937
GOTERM_BP_DIRECT	GO:0097533~cellular stress response to acid chemical	0.049102937
GOTERM_BP_DIRECT	GO:0070172~positive regulation of tooth mineralization	0.049102937
GOTERM_BP_DIRECT	GO:0055096~low-density lipoprotein particle mediated signaling	0.049102937
GOTERM_BP_DIRECT	GO:0016310~phosphorylation	0.049248289
GOTERM_CC_DIRECT	GO:0005737~cytoplasm	1.48E-07
GOTERM_CC_DIRECT	GO:0030018~Z disc	7.25E-06
GOTERM_CC_DIRECT	GO:0005829~cytosol	3.68E-05
GOTERM_CC_DIRECT	GO:0005604~basement membrane	3.62E-04
GOTERM_CC_DIRECT	GO:0005615~extracellular space	7.24E-04
GOTERM_CC_DIRECT	GO:0008076~voltage-gated potassium channel complex	8.70E-04
GOTERM_CC_DIRECT	GO:0042383~sarcolemma	0.001040121
GOTERM_CC_DIRECT	GO:0031430~M band	0.001351155
GOTERM_CC_DIRECT	GO:0090575~RNA polymerase II transcription factor complex	0.004027975
GOTERM_CC_DIRECT	GO:0005739~mitochondrion	0.004094107
GOTERM_CC_DIRECT	GO:0030315~T-tubule	0.004420445
GOTERM_CC_DIRECT	GO:0005634~nucleus	0.005223739
GOTERM_CC_DIRECT	GO:0016324~apical plasma membrane	0.005284476
GOTERM_CC_DIRECT	GO:0005654~nucleoplasm	0.007312053
GOTERM_CC_DIRECT	GO:0016323~basolateral plasma membrane	0.00865491
GOTERM_CC_DIRECT	GO:0030424~axon	0.013983743
GOTERM_CC_DIRECT	GO:0005576~extracellular region	0.016405812
GOTERM_CC_DIRECT	GO:0031012~extracellular matrix	0.020196863
GOTERM_CC_DIRECT	GO:0030054~cell junction	0.024109536
GOTERM_CC_DIRECT	GO:0000164~protein phosphatase type 1 complex	0.02612586

GOTERM_CC_DIRECT	GO:0009897~external side of plasma membrane	0.027899633
GOTERM_CC_DIRECT	GO:0005581~collagen trimer	0.029243643
GOTERM_CC_DIRECT	GO:0016605~PML body	0.031147726
GOTERM_CC_DIRECT	GO:0043197~dendritic spine	0.031147726
GOTERM_CC_DIRECT	GO:0005856~cytoskeleton	0.035527991
GOTERM_CC_DIRECT	GO:0031982~vesicle	0.039526599
GOTERM_CC_DIRECT	GO:0009986~cell surface	0.040168864
GOTERM_CC_DIRECT	GO:0043235~receptor complex	0.048349932
GOTERM_MF_DIRECT	GO:0001228~transcriptional activator activity, RNA polymerase II transcription regulatory region sequence-specific binding	9.75E-06
GOTERM_MF_DIRECT	GO:0046872~metal ion binding	2.77E-05
GOTERM_MF_DIRECT	GO:0061629~RNA polymerase II sequence-specific DNA binding transcription factor binding	1.74E-04
GOTERM_MF_DIRECT	GO:0003700~transcription factor activity, sequence-specific DNA binding	4.73E-04
GOTERM_MF_DIRECT	GO:0000978~RNA polymerase II core promoter proximal region sequence-specific DNA binding	5.31E-04
GOTERM_MF_DIRECT	GO:0000981~RNA polymerase II transcription factor activity, sequence-specific DNA binding	5.32E-04
GOTERM_MF_DIRECT	GO:0042802~identical protein binding	6.62E-04
GOTERM_MF_DIRECT	GO:0005249~voltage-gated potassium channel activity	8.39E-04
GOTERM_MF_DIRECT	GO:0004114~3',5'-cyclic-nucleotide phosphodiesterase activity	0.002423692
GOTERM_MF_DIRECT	GO:0051015~actin filament binding	0.002580768
GOTERM_MF_DIRECT	GO:0016616~oxidoreductase activity, acting on the CH-OH group of donors, NAD or NADP as acceptor	0.003643048
GOTERM_MF_DIRECT	GO:0004115~3',5'-cyclic-AMP phosphodiesterase activity	0.003783572
GOTERM_MF_DIRECT	GO:0030297~transmembrane receptor protein tyrosine kinase activator activity	0.003783572
GOTERM_MF_DIRECT	GO:0005516~calmodulin binding	0.00423067
GOTERM_MF_DIRECT	GO:0005112~Notch binding	0.004436488
GOTERM_MF_DIRECT	GO:0044325~ion channel binding	0.004833968
GOTERM_MF_DIRECT	GO:0016491~oxidoreductase activity	0.005387806
GOTERM_MF_DIRECT	GO:0003779~actin binding	0.006043642
GOTERM_MF_DIRECT	GO:0030552~cAMP binding	0.007003682
GOTERM_MF_DIRECT	GO:0005518~collagen binding	0.009837508
GOTERM_MF_DIRECT	GO:0008270~zinc ion binding	0.010717606
GOTERM_MF_DIRECT	GO:0005201~extracellular matrix structural constituent	0.01130036
GOTERM_MF_DIRECT	GO:0048018~receptor agonist activity	0.012465158
GOTERM_MF_DIRECT	GO:0042803~protein homodimerization activity	0.013214967
GOTERM_MF_DIRECT	GO:0046983~protein dimerization activity	0.01341467
GOTERM_MF_DIRECT	GO:0031432~titin binding	0.020347432
GOTERM_MF_DIRECT	GO:0005388~calcium-transporting ATPase activity	0.020347432

GOTERM_MF_DIRECT	GO:2001069~glycogen binding	0.020347432
GOTERM_MF_DIRECT	GO:0008970~phosphatidylcholine 1-acylhydrolase activity	0.020347432
GOTERM_MF_DIRECT	GO:0000976~transcription regulatory region sequence-specific DNA binding	0.024483356
GOTERM_MF_DIRECT	GO:0035259~glucocorticoid receptor binding	0.025014385
GOTERM_MF_DIRECT	GO:0032794~GTPase activating protein binding	0.025014385
GOTERM_MF_DIRECT	GO:0031625~ubiquitin protein ligase binding	0.026842129
GOTERM_MF_DIRECT	GO:0005154~epidermal growth factor receptor binding	0.027008418
GOTERM_MF_DIRECT	GO:0005515~protein binding	0.031166702
GOTERM_MF_DIRECT	GO:0070888~E-box binding	0.032563854
GOTERM_MF_DIRECT	GO:0045296~cadherin binding	0.035312718
GOTERM_MF_DIRECT	GO:0051371~muscle alpha-actinin binding	0.035490269
GOTERM_MF_DIRECT	GO:0008092~cytoskeletal protein binding	0.039185131
GOTERM_MF_DIRECT	GO:0043565~sequence-specific DNA binding	0.044016491
GOTERM_MF_DIRECT	GO:0004879~RNA polymerase II transcription factor activity, ligand-activated sequence-specific DNA binding	0.049841544
GOTERM_MF_DIRECT	GO:0052740~1-acyl-2-lysophosphatidylserine acylhydrolase activity	0.049842146
GOTERM_MF_DIRECT	GO:0052739~phosphatidylserine 1-acylhydrolase activity	0.049842146
KEGG_PATHWAY	ssc04020:Calcium signaling pathway	2.09E-06
KEGG_PATHWAY	ssc04931:Insulin resistance	2.15E-05
KEGG_PATHWAY	ssc05415:Diabetic cardiomyopathy	1.25E-04
KEGG_PATHWAY	ssc04261:Adrenergic signaling in cardiomyocytes	1.35E-04
KEGG_PATHWAY	ssc05200:Pathways in cancer	1.94E-04
KEGG_PATHWAY	ssc04024:cAMP signaling pathway	3.79E-04
KEGG_PATHWAY	ssc04933:AGE-RAGE signaling pathway in diabetic complications	7.50E-04
KEGG_PATHWAY	ssc04922:Glucagon signaling pathway	8.11E-04
KEGG_PATHWAY	ssc04919:Thyroid hormone signaling pathway	8.63E-04
KEGG_PATHWAY	ssc04152:AMPK signaling pathway	9.90E-04
KEGG_PATHWAY	ssc04260:Cardiac muscle contraction	0.001073821
KEGG_PATHWAY	ssc04010:MAPK signaling pathway	0.001272145
KEGG_PATHWAY	ssc05410:Hypertrophic cardiomyopathy	0.002312738
KEGG_PATHWAY	ssc05418:Fluid shear stress and atherosclerosis	0.002829582
KEGG_PATHWAY	ssc05202:Transcriptional misregulation in cancer	0.003469345
KEGG_PATHWAY	ssc04974:Protein digestion and absorption	0.003714524
KEGG_PATHWAY	ssc04066:HIF-1 signaling pathway	0.00507065
KEGG_PATHWAY	ssc04068:FoxO signaling pathway	0.005305112
KEGG_PATHWAY	ssc04920:Adipocytokine signaling pathway	0.005810092
KEGG_PATHWAY	ssc03320:PPAR signaling pathway	0.008313759
KEGG_PATHWAY	ssc04148:Efferocytosis	0.008514035
KEGG_PATHWAY	ssc04022:cGMP-PKG signaling pathway	0.010585216

KEGG_PATHWAY	ssc05412:Arrhythmogenic right ventricular cardiomyopathy	0.011533963
KEGG_PATHWAY	ssc00410:beta-Alanine metabolism	0.015875964
KEGG_PATHWAY	ssc04380:Osteoclast differentiation	0.016039094
KEGG_PATHWAY	ssc04658:Th1 and Th2 cell differentiation	0.020529196
KEGG_PATHWAY	ssc04964:Proximal tubule bicarbonate reclamation	0.026356645
KEGG_PATHWAY	ssc01212:Fatty acid metabolism	0.026666365
KEGG_PATHWAY	ssc04916:Melanogenesis	0.027811527
KEGG_PATHWAY	ssc05414:Dilated cardiomyopathy	0.032038744
KEGG_PATHWAY	ssc05165:Human papillomavirus infection	0.035548505
KEGG_PATHWAY	ssc05010:Alzheimer disease	0.037405296
KEGG_PATHWAY	ssc00071:Fatty acid degradation	0.038073067
KEGG_PATHWAY	ssc05208:Chemical carcinogenesis - reactive oxygen species	0.03822563
KEGG_PATHWAY	ssc04926:Relaxin signaling pathway	0.038317903
KEGG_PATHWAY	ssc05205:Proteoglycans in cancer	0.043345296
KEGG_PATHWAY	ssc05022:Pathways of neurodegeneration - multiple diseases	0.044239349
KEGG_PATHWAY	ssc04932:Non-alcoholic fatty liver disease	0.045973424
KEGG_PATHWAY	ssc04640:Hematopoietic cell lineage	0.048919484
KEGG_PATHWAY	ssc04910:Insulin signaling pathway	0.049706603

E100＞E70

Category	Term	PValue
GOTERM_BP_DIRECT	GO:1990573~potassium ion import across plasma membrane	0.006708465
GOTERM_BP_DIRECT	GO:0032922~circadian regulation of gene expression	0.011949833
GOTERM_BP_DIRECT	GO:2001171~positive regulation of ATP biosynthetic process	0.017017745
GOTERM_BP_DIRECT	GO:0043065~positive regulation of apoptotic process	0.018728955
GOTERM_BP_DIRECT	GO:0030007~cellular potassium ion homeostasis	0.042012703
GOTERM_BP_DIRECT	GO:0036376~sodium ion export from cell	0.044751113
GOTERM_CC_DIRECT	GO:0005737~cytoplasm	0.020129034
GOTERM_CC_DIRECT	GO:0005829~cytosol	0.021336162
GOTERM_CC_DIRECT	GO:0005890~sodium:potassium-exchanging ATPase complex	0.032871395
GOTERM_MF_DIRECT	GO:0005391~sodium:potassium-exchanging ATPase activity	0.024243141
KEGG_PATHWAY	ssc05415:Diabetic cardiomyopathy	1.47E-04
KEGG_PATHWAY	ssc04931:Insulin resistance	9.37E-04
KEGG_PATHWAY	ssc04964:Proximal tubule bicarbonate reclamation	0.003444327
KEGG_PATHWAY	ssc04922:Glucagon signaling pathway	0.007577455
KEGG_PATHWAY	ssc04910:Insulin signaling pathway	0.016941111

KEGG_PATHWAY	ssc04261:Adrenergic signaling in cardiomyocytes	0.021543838
KEGG_PATHWAY	ssc04920:Adipocytokine signaling pathway	0.034480285
KEGG_PATHWAY	ssc04971:Gastric acid secretion	0.035349893
KEGG_PATHWAY	ssc05202:Transcriptional misregulation in cancer	0.037537784
KEGG_PATHWAY	ssc04911:Insulin secretion	0.04546201
